# Germline-targeting HIV immunogen induces cross-neutralizing antibodies in outbred macaques

**DOI:** 10.1016/j.immuni.2026.03.012

**Published:** 2026-04-14

**Authors:** Nitesh Mishra, Bo Liang, Ryan S. Roark, Amrit Ghosh, Sean Callaghan, Wen-Hsin Lee, Xuduo Li, Anh L. Vo, Gabriel Avillion, Rohan Roy Chowdhury, Rumi Habib, Frederic Bibollet-Ruche, Gabriella Giese, Prabhgun Oberoi, Khaled Amereh, Anjali Somanathan, Yuxin Zhu, Yuexiu Zhang, Muzaffer Kassab, Lifei Tjio, Sharaf Andrabi, Raphael A. Reyes, Joel D. Allen, Nicole E. James, Kipchoge N. Randall, Lara van der Maas, Elana Ben-Akiva, Kasia Kacmarek-Michaels, Samantha Plante, Christian L. Martella, Ashwin N. Skelly, Ajay Singh, Jonathan Hurtado, Katharina Dueker, Tazio Capozzola, Rebecca Nedellec, Gabriel Ozorowski, Mark M. Lewis, Samantha Falcone, Andrea Carfi, Sunny Himansu, Lawrence Shapiro, Max Crispin, Beatrice H. Hahn, Bryan Briney, Darrell J. Irvine, Dennis R. Burton, Andrew B. Ward, Facundo D. Batista, Peter D. Kwong, George M. Shaw, Raiees Andrabi

**Affiliations:** 1 Department of Immunology and Microbiology, The Scripps Research Institute, La Jolla, CA 92037, USA; 2 Consortium for HIV/AIDS Vaccine Development (CHAVD), The Scripps Research Institute, La Jolla, CA 92037, USA; 3 Department of Medicine, Perelman School of Medicine, University of Pennsylvania, Philadelphia, PA 19104, USA; 4 Aaron Diamond AIDS Research Center, Columbia University Vagelos College of Physicians and Surgeons, New York, NY 10032, USA; 5 Department of Biochemistry and Molecular Biophysics, Columbia University, New York, NY 10027, USA; 6 Zuckerman Mind Brain Behavior Institute, Columbia University, New York, NY 10027, USA; 7 Ragon Institute of Massachusetts General Hospital, Massachusetts Institute of Technology, and Harvard University, Cambridge, MA 02114, USA; 8 Department of Integrative Structural and Computational Biology, The Scripps Research Institute, La Jolla, CA 28 92037, USA; 9 Department of Microbiology, Perelman School of Medicine, University of Pennsylvania, Philadelphia, PA 19104, USA; 10 School of Biological Sciences, University of Southampton, Southampton SO17 1BJ, UK; 11 Koch Institute for Integrative Cancer Research, Massachusetts Institute of Technology, Cambridge, MA 02139; 12 Bioqual, Inc., Rockville, Maryland, USA; 13 Moderna, Inc., Cambridge, Ma 02139, USA; 14 Howard Hughes Medical Institute, Chevy Chase, MD 20815; 15 IAVI Neutralizing Antibody Center, The Scripps Research Institute, La Jolla, CA 92037, USA; 16 Department of Biology, Massachusetts Institute of Technology, Cambridge, MA 02139, USA; 17 Lead Contact

## Abstract

Germline-targeting-(GT) is a promising strategy to activate rare broadly neutralizing antibody (bnAb)-producing B cells against HIV, but induction of such responses in outbred animals has not been achieved. Using antibody-guided structure-based design, we engineered a germlinetargeting HIV trimer immunogen, Q23-APEX-GT2, that primes diverse V2-apex bnAb precursors. Q23-APEX-GT2 efficiently activated rare V2-apex-specific B cells in humanized knock-in mice and consistently elicited immunofocused antibody responses in outbred rhesus macaques, priming multiple long CDRH3-loop bnAb-B cell lineages. Monoclonal antibodies isolated from immunized macaques showed broad heterologous HIV trimer recognition and modest cross-neutralization of diverse tier-2 viruses. Cryo-EM structural studies confirmed precise epitope targeting and revealed CDRH3-mediated binding modes that mirrored those of human V2-apex bnAbs. Together, these findings establish proof-of-principle for priming and early maturation of authentic V2-apex bnAb precursors in outbred macaques and highlight the promise of V2-apex–targeted HIV vaccines.

## eTOC blurb

Inducing broadly neutralizing antibodies (bnAbs) remains a challenge in HIV vaccine development. Mishra et al. show that an engineered germline-targeting HIV trimer, Q23-APEX-GT2, primes rare V2-apex bnAb precursors in humanized mice and outbred macaques, eliciting antibodies with modest neutralization breadth. Structural analyses confirm V2-apex bnAb-site targeting and diverse CDRH3-mediated binding modes resembling human bnAbs, providing proof-of-principle for targeted vaccine strategies.

## Graphical Abstract

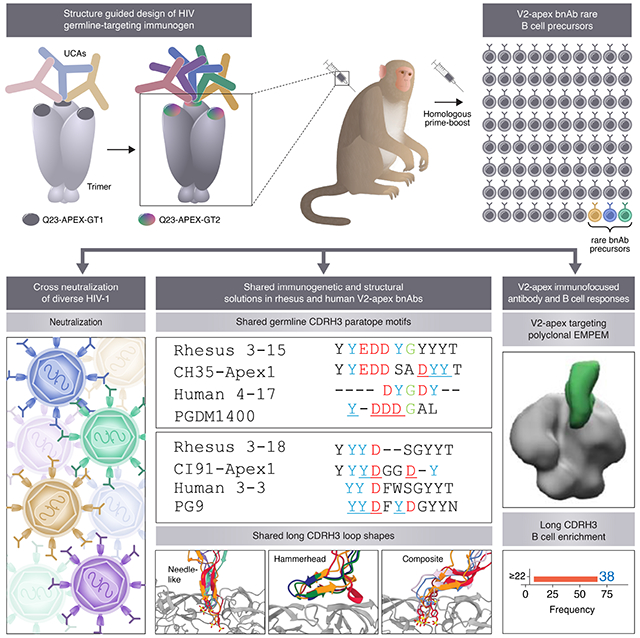

## INTRODUCTION

A major goal of HIV vaccine research is to elicit broadly neutralizing antibodies (bnAbs), which have been shown to be protective both in non-human primate and human studies ^1–4^. Although some HIV-infected individuals eventually develop bnAbs, this occurs infrequently ^5–11^ due, at least in part, to bnAbs being encoded by rare B cell precursors and requiring complex affinity maturation pathways ^7,12–19^, making their induction through vaccination especially challenging. Accordingly, many recent HIV vaccine strategies have focused on activating rare bnAb-producing B cell precursors via germline-targeting (GT) immunogens, followed by rationally designed boosting strategies ^18,20–29^. This approach has demonstrated promise in priming bnAb B cell precursors in both preclinical animal models and human trials ^30–39^, although no studies to date have induced bnAbs, either in B memory cells or secreted antibody, in stringent outbred animal models.

One of the most promising targets for HIV vaccines is the V2-apex bnAb site ^13,27,28,40–45^. Antibodies targeting this epitope are typically potent and broad, occur early in infection, require relatively low levels of somatic mutations, and are among the most common to occur in natural HIV infection ^5,6,8,45–52^. These V2-apex bnAbs target a lysine-rich patch on the V2 C-strand and neighboring glycans near the three-fold axis of the Env trimer, making these antibodies generally trimer-specific ^28,41,53–59^. A critical feature of these bnAbs is a long anionic heavy-chain complementarity-determining region 3 (CDRH3), which enables them to penetrate the glycan shield and reach the underlying cationic protein surface ^41,55,56,60^. However, a significant barrier to eliciting V2-apex bnAbs is the rarity of human B cell precursors with long CDRH3 loops ^13,61,62^. Nonetheless, these rare bnAb precursors possess germline D-gene-encoded CDRH3 paratope features that could be exploited by targeted vaccines, making a germline-targeting approach potentially very effective ^13,28,41,43,63^. We hypothesized that the key obstacle to the elicitation of V2-apex bnAbs is the development of trimer immunogens that closely mirror the native Env of wildtype infectious virions but also contain select engineered mutations that substantially enhance their affinity for diverse germline B cell bnAb precursors, or unmutated common ancestors (UCAs). This strategy ensures that germline B cell precursors that have the potential for development into actual bnAbs are preferentially primed, and once activated, affinity-mature to bind native-like Env structures including those on heterologous viruses.

In this study, by using antibody-guided structure-based vaccine design, we generated a germline-targeting trimer immunogen (Q23.APEX-GT2) to activate rare V2-apex bnAb B cell precursors. Immunization in outbred rhesus macaques elicited a consistent antibody response highly focused to the HIV Env V2-apex bnAb site. Isolation of monoclonal antibodies (mAbs) from antigen specific B cells showed the expansion of diverse V2-apex bnAb site-targeting long CDRH3 B cells across multiple vaccinated macaques. These mAbs bound a variety of heterologous Env trimers representing the global HIV-1 diversity. A subset of these mAbs exhibited modest neutralization breadth against tier 2 heterologous viruses, providing evidence of vaccine-induced heterologous neutralization in outbred macaques. Structural studies of the isolated antibodies confirmed targeting of the V2-apex bnAb site in diverse binding modes mirroring those observed in human and rhesus V2-apex bnAbs elicited by HIV and SHIV infection ^41,53,55–58^. Thus, our study provides proof-of-principle that a germline-targeting immunogen can induce authentic V2-apex bnAb precursors and drive their partial maturation into cross-neutralizing antibodies in an outbred nonhuman primate model. Notably, this occurred after a homologous prime and boost vaccination with a single germline-targeting trimer immunogen. Overall, the results suggest that V2-apex bnAb elicitation may be relatively straightforward than other HIV Env bnAb sites, requiring limited boosting and relatively few somatic hypermutations, provided that the appropriate rare B cells with long CDRH3 loops can be effectively primed.

## RESULTS

### Rational design of Q23-APEX-GT2 with enhanced binding to V2-apex bnAb UCAs

Germline-targeting (GT) has emerged as a promising approach in HIV-1 vaccine design, aiming to activate rare broadly neutralizing antibody (bnAb) precursors by engineering high-affinity immunogens. However, many GT strategies focus narrowly on engaging only certain antibody lineages, which may limit the breadth of response against diverse virus strains. In contrast, we employed a alternate GT-approach designed to target a wider array of HIV Env V2-apex bnAb precursors, irrespective of their paratope structural diversity. To this end, we previously showed that V2-apex bnAbs possess germline D-gene-encoded anionic residues (YYD) that enable them to interact readily with the positively charged V2-apex core bnAb epitope ^28,41^. We also demonstrated that a unique set of HIV Envs naturally have a unique glycan hole that facilitates binding of V2-apex bnAb unmutated common ancestors (UCAs) in the native-like trimer configurations ^43^. When these unique Envs were delivered into rhesus macaques as SHIVs in an infection model, one of them, Q23.17, reproducibly induced V2-apex bnAbs, indicating an inherent propensity of this Env to elicit V2-apex site-targeted bnAb responses ^58,64^

Using an antibody-guided structure-based design approach, we recently developed a Q23.17-based, prefusion-stabilized, V2-apex germline-targeted trimer (Q23-APEX-GT1) that exhibited native-like trimer properties and primed rhesus V2-apex bnAb UCA knock-in B cells, resulting in moderate neutralization breadth and potency ^65,66^. Here, we utilized a large panel of *bona fide* V2-apex bnAb UCAs from SHIV-infected rhesus macaques ^64^, along with molecular information on how V2-apex bnAbs bind to HIV trimers (Figure S1) ^58^, to improve the broad UCA binding properties of Q23-APEX-GT1. Our goal was to design a germline-targeting trimer version of Q23-APEX-GT1 capable of priming a broader range of divers V2-apex bnAb precursors, thereby expanding the pool of B cells that can be targeted for HIV bnAb development.

**Figure 1.**
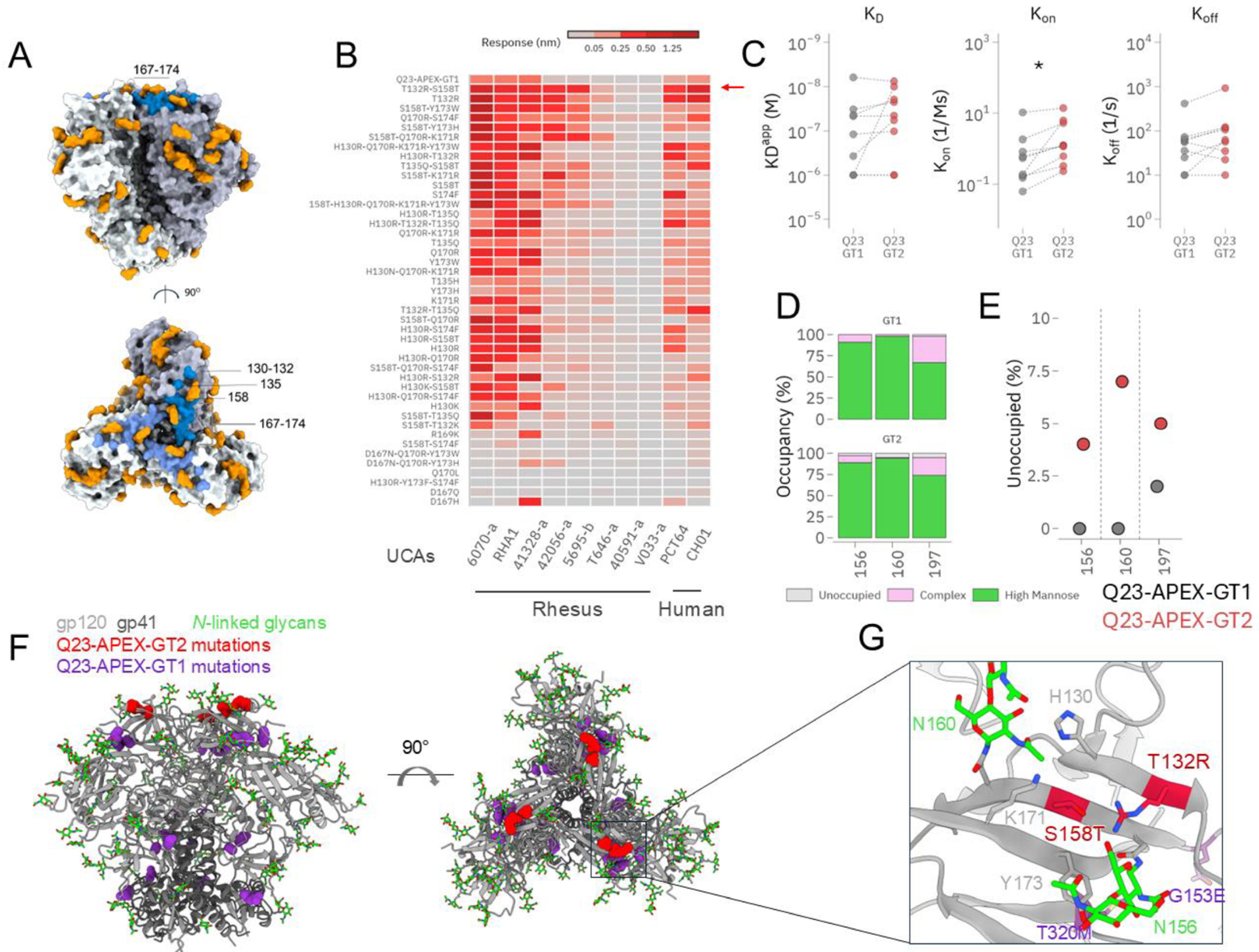
Antibody-guided structure-based design of Q23-APEX-GT2 for enhanced binding to diverse prototype V2-apex bnAb precursors. See also Figures S1, S2, S3 and S4. A. V1V2 residues (130, 132, 158, 167-174) that potentially interact with V2-apex bnAbs, and/or their precursors are shown on crystal structure of Q23 DS-SOSIP (PDB: 7LLK). Residues are colored blue while glycans are shown in orange. B. Heat map representing the BLI binding response of a broad V2-apex UCA panel against V1V2 mutants generated on Q23-SCT27 or Q23-APEX-GT1 backbone. First row in the heat map shows the binding against the base construct (Q23-APEX-GT1 or GT1). Mutants are ordered based on average binding across the UCA panel. 8 rhesus (6070, RHA1, 41328, 42056, RHA2, T646, 40591 and V033) and 4 human (PCT64, CH01, PG9 and CAP256) UCAs were included in the BLI binding screen. PG9 and CAP256 UCA showed no binding. Based on broad binding to UCAs, Q23-APEX-GT1-T132R-S158T variant, designated as Q23-APEX-GT2 (or GT2 – indicated by red arrow) was down-selected. C. Apparent binding affinity constants (KD), on-rate constants (Kon) and off-rate constants (K_off_) of base construct (GT1, gray) compared to germline-targeting lead candidate (GT2, red). A significant increase in K_on_ was observed (p < 0.05). D. Glycan occupancy assessed by proteomics-based site-specific glycan analysis (SSGA) at key V2-apex glycans (position 156, 160 and 197) in GT1 and GT2. High mannose in faded green; complex glycan is shown in light pink; unoccupied fraction is shown in gray. E. Increased unoccupied fractions at position 156, 160 and 197 seen for GT2 (red). F. Cryo-EM structure of germline-targeting immunogen Q23-APEX-GT2 showing a compact assembly and trimeric apex. gp120 is colored light gray, gp41 is colored dark gray, glycans are colored green. GT1 mutations are shown in violet. GT2 mutations are at the apex and shown in red. G. Close-up view of potential interactions between key GT2 mutations 132R with the N156 glycans at the apex of GT2.

Starting with the recently stabilized Q23-APEX-GT1 trimer as a base construct, we generated a large number of variants by introducing additional antibody-trimer structure-based amino acid mutations alone and in combination within the V1V2 region at residues 130, 132, 135, 158, 167, 169, 170, 171, 173 (HXB2 reference numbering) (Figure 1A–B, S1). By testing the binding of these mutants to diverse V2-apex bnAb UCAs, we identified Q23-APEX-GT1-T132R-S158T (designated Q23-APEX-GT2) as the most improved construct, since it bound 10 of 12 rhesus and human V2-apex bnAb UCAs with discernable affinity (Figure 1B–C). The optimized Q23-APEX-GT2 trimer showed substantial K_D_ affinity improvements (Figure 1C, S2), driven primarily by enhanced on-rate constants (K_on_) (p < 0.05), highlighting its improved germline precursor engagement—a key feature for germline-targeting immunogens. Since the S158T mutation changes the N156 glycan sequon from NxS to NxT, we performed site-specific glycan analysis of Q23-APEX-GT2 using mass spectrometry to determine the glycan composition and occupancy. Overall, the glycan profile of Q23-APEX-GT2 was similar to that of the base construct Q23-APEX-GT1, except for heterogeneity in glycan maturation (predominantly displaying mannose-rich glycans) and slightly reduced occupancy (up to 7%) at glycan positions N156, N160 and N197 (Figure 1D–E, S2D). The abundance of oligomannose-type glycans further indicates the assembly of a well folded native-like trimer ^67^. The antigenicity of Q23-APEX-GT2 was similar to that of Q23-APEX-GT1 as demonstrated by the binding profiles of a panel of HIV bnAbs and non-neutralizing antibodies (nnAbs) targeting various Env epitopes (Figure S2E). This conservation of glycan presentation and native-like trimer antigenicity supports the structural integrity of the engineered Q23-APEX-GT2 trimer. Finally, we tested a membrane-bound version of Q23-APEX-GT2 as a potential mRNA-based immunogen and found that it exhibited a similar binding profile to V2-apex bnAb precursors and mature bnAbs compared to the soluble protein, with no negative impact on its overall antigenic profile (Figure S2C, S2F). Q23-APEX-GT2 maintained an HIV tier 2 Env antigenic and neutralization sensitivity profile, demonstrating binding and neutralization by bnAbs targeting canonical Env bnAb sites while showing minimal to no binding or neutralization by non-neutralizing antibodies (nnAbs) that recognize “open” Env structures (Figure S2G–J, Supplementary Table S1).

To confirm a native-like trimer conformation, we resolved the structure of Q23-APEX-GT2 at 2.9 Å resolution using single-particle cryogenic electron microscopy (cryo-EM). This analysis revealed a compact trimeric apex consistent with the prefusion-closed conformation of Env (Figure 1F, S3). Further examination of the local environment of engineered residues T132R and S158T revealed the introduction of an interactive network spanning residues 132, 156, 158, 171, and the first *N*-acetylglucosamine residue of N156-glycan that is absent in Q23-APEX-GT1, suggesting that fortification of interactions across the A-, B-, and C-strands of the trimer apex contributes to the observed enhanced antibody binding (Figure 1G and S3A – D). Trimer apex-stabilization was also achieved through the initial Q23-APEX-GT1 modifications, including the T320M mutation which fills a hydrophobic pocket lined by V2 residues 154, 175, and 177, and the G153E mutation, which is known to suppress exposure of the V3 loop (Figure S3C – D). Thus, the structural data suggest a synergy between the apex-stabilizing mutations of GT1 and the breadth-enhancing mutations of GT2, which together are likely responsible for the improved binding of the Q23-APEX-GT2 trimer to a broad spectrum of human and rhesus V2-apex bnAb precursors, regardless of their structural class ^58^.

### The engineered Q23-APEX-GT2 trimer efficiently primes rare human V2-apex bnAb UCA knock-in B cell precursors

**Figure 2.**
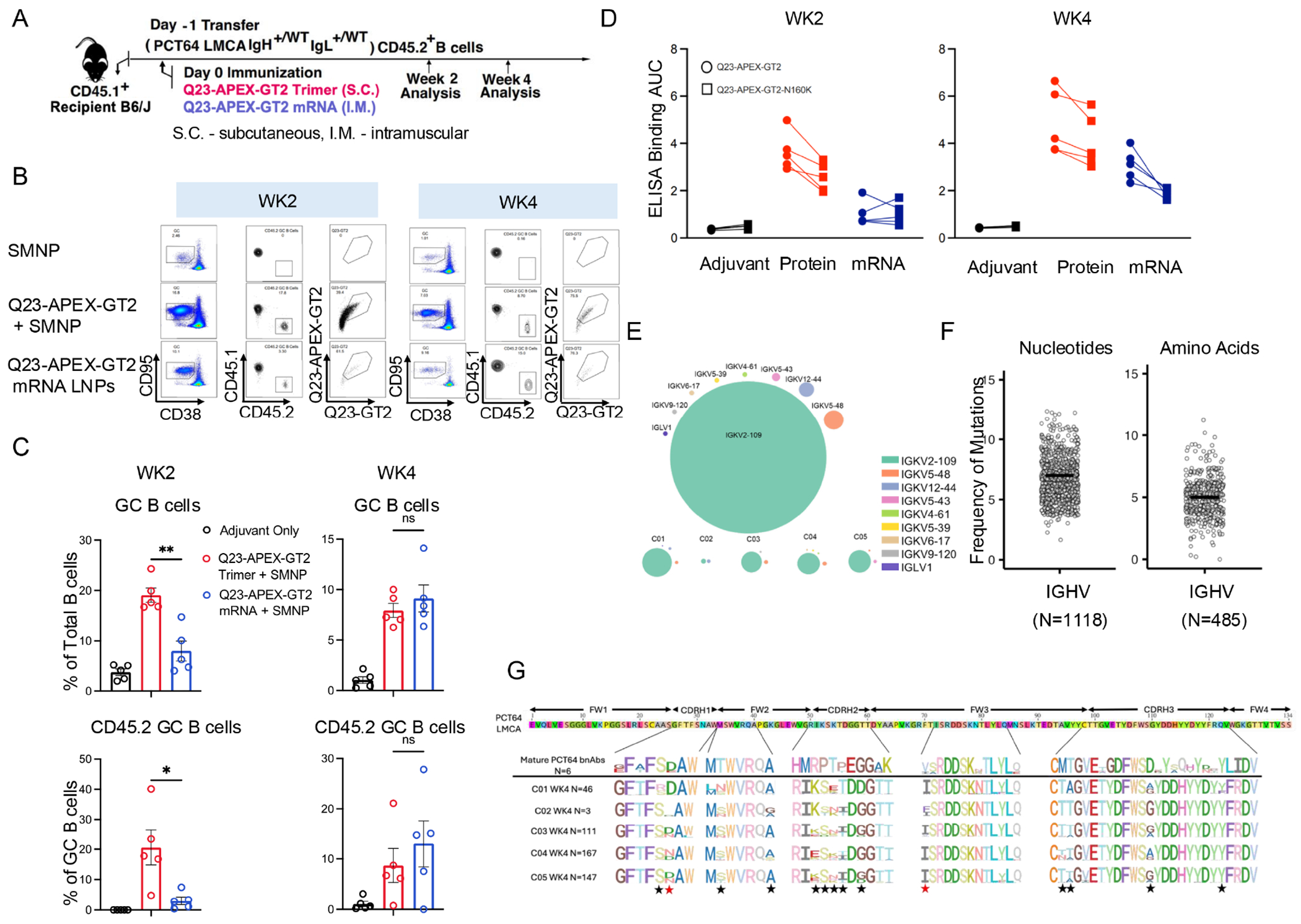
Q23-APEX-GT2 trimer successfully primes human V2-apex bnAb precursor PCT64^LMCA^ encoding rare B cells. A. Schematic representation of PCT64^LMCA^ mouse immunization studies. 1 x 10^5^ B cells of CD45.2-encoded PCT64^LMCA^ precursors were injected intravenously into the tail vein of CD45.1 WT mice recipients (B6.SJL-Ptprca Pepcb/BoyJ). Post adoptive transfer mice were immunized with SMNP adjuvant [(5μg) (Arm 1 - control)], Q23-APEX-GT2 protein + adjuvant [(20μg protein + 5μg adjuvant) (Arm 2)] and membrane-anchored Q23-APEX-GT2 mRNA lipid nanoparticle (LNPs) [(1μg mRNA LNPs) (Arm 3)]. The adjuvant or protein + adjuvant was administered subcutaneously and the mRNA LNPs were injected by intramuscular route. Lymph nodes and immune sera were collected at week 2 and 4 time points. B. Flow cytometry plots of B cells from draining LN at 2- and 4-weeks post immunization from a representative animal. Germinal Center (GC) B cell and CD45.2 GC B cell responses to Q23-APEX-GT2 trimer in weeks 2 (left) and 4 (right) post immunization are shown. C. Quantification of GC B cells and CD45.2 GC B cells in week 2 (left) and week 4 (right) post immunization with SMNP adjuvant only, Q23-APEX-GT2 trimer protein with SMNP and Q23-APEX-GT2 mRNA LNPs (n=5). Each dot in the scatter bar plot represents an individual animal, and data are shown as mean ± SEM. D. The ELISA binding AUC titers of serum binding to Q23-APEX-GT2 and the V2-apex epitope knock-out (N160K) at wk2 (left) and WK4 (right) post-immunization. E. Distribution of IG Kappa/Lambda V gene usage in Q23-APEX-GT2 protein vaccinated individual mice (C01–C05). The antibody sequences were derived from LN B cells harvested at week 4 post immunization. Data are shown as bubble plots, where the circle size represents its proportion of the total V gene count. Light chain enrichment breakdown by individual mice in sublots. F. Somatic hypermutation (SHM) percentages in the Q23-APEX-GT2 vaccination derived PCT64^LMCA^ heavy chains. SHM percentages were calculated using both nucleotide and amino acid sequences of the v-gene. Each dot represents a sequence compared to the PCT64 germline, with the black line indicating the mean number of mutations from all animals in the group. G. Sequence logograms across individual mice obtained from week 4 (bottom 5 rows) of highly mutated residues, compared with the least mutated common ancestor (LMCA, top line) of PCT64 and six mature PCT64 bnAbs. SHMs specific to mouse immunization (black) and shared with PCT64 mature bnAbs (red). p values were calculated by a Mann-Whitney test (A and H). *p < 0.05; ns, not significant. Error bars are SEM

To evaluate the *in vivo* priming efficacy of engineered Q23-APEX-GT2, we tested its ability to engage and expand precursors of a defined V2-apex bnAb lineage using a knock-in mouse model. We selected a mouse model with B cells bearing the least mutated common ancestor (LMCA) heavy chain (IGH) and light chain (IGK) of bnAb PCT64, which was isolated from a human donor, as the PCT64 class is among the most prevalent in the human naïve B cell repertoire ^13,42,46^. Given the high frequency of PCT64^LMCA^ B cells in our KI mouse model, we used an adoptive transfer system where (1x10^5^) CD45.2^+^ PCT64^LMCA^ B cells were transferred into congenic CD45.1^+^ C57BL/6J recipient mice to decrease precursor frequency to try to mimic the rare precursor frequency observed in humans. This adoptive transfer resulted in approximately 20 antigen-specific CD45.2 B cells per 1x10^6^ B cells in spleen^42^. Cohorts of these adoptively transferred mice were immunized with: SMNP (saponin/MPLA nanoparticles) adjuvant alone (Arm 1 – control), Q23-APEX-GT2 trimer protein + SMNP (Arm 2) and membrane-anchored Q23-APEX-GT2 trimer mRNA lipid nanoparticles (Arm 3) (Figure 2A). To assess the priming efficiency of Q23-APEX-GT2, we harvested draining lymph nodes (LNs) at weeks 2 and 4 post-vaccination and analyzed antigen-specific B cell responses via flow cytometry (Figure 2B–C). Enhanced LN germinal center (GC: CD95^hi^CD38^lo^) B cell responses were observed in both Q23-APEX-GT2 protein and mRNA groups compared to the adjuvant-only control. At week 2, the protein group showed substantially higher GC B cell response compared with the mRNA group (20% vs 8%), but by week 4 both groups were comparable (7% vs 9%). A substantial proportion of LN GC B cells were PCT64^LMCA^ CD45.2 B cells (20% vs 2%, protein vs mRNA) at both time points, indicating efficient priming, expansion and retention of the rare human V2-apex bnAb precursors in the GCs (Figure 2B–C). Consistent with these findings, in a separate study using a rhesus macaque V2-apex bnAb UCA knock-in model (V033 UCA), we show that a precursor version of the immunogen, Q23-APEX-GT1, elicited higher frequency of V2-apex epitope-specific GC B cell responses at week 4 when delivered either as a SMNP-adjuvanted protein or as self-amplifying RNA (saRNA) formulated in lipid nanoparticles (LNPs)^65^. The responses were comparable between the protein and the saRNA groups. These data collectively suggest that both protein and mRNA formats are capable of priming V2-apex bnAb precursors, although the kinetics and magnitude of the early response may differ depending on the delivery platform.

Consistent with GC formation, we observed that polyclonal sera collected at weeks 2 and 4 post-prime exhibited Q23-APEX-GT2-specific antibody binding (Figure 2D). At week 4, a substantial fraction targeted the V2-apex bnAb site in both cohorts, as confirmed by ELISA binding to Q23-APEX-GT2 trimer and its N160K variant lacking the critical N160 glycan though mRNA immunized mice showed slightly higher binding to GT2-N160K mutant at week 2; N160K substitution abrogates PCT64 and other V2-apex antibody binding ^28,42,46^.

To assess whether PCT64^LMCA^ B cells underwent somatic hypermutation (SHM) and acquired PCT64-like mutations following Q23-APEX-GT2 immunization, we isolated class-switched Q23 APEX-GT2+ PCT64^LMCA^ B cells at week 4 post immunization for 10X B cell receptor (BCR) sequencing from five protein-vaccinated mice (C01–C05). Sequence analysis revealed a strong selection for the IGKV2-109 mouse light chain paired with the PCT64^LMCA^ heavy chain in all five mice (Figure 2E). Analysis of PCT64^LMCA^ IGH lineage evolution revealed extensive diversification by week 4 (Figure 2F, G). By week 4 post-immunization, substantial SHM were observed in the IGH V region (~5% at the amino acid sequence level). Mutations accumulated in recurrent positions within CDRH1, CDRH2, and CDRH3 over time. Further analysis showed antigen-driven convergent SHM patterns across mice, with some mutations occurring at positions shared with mature PCT64 bnAbs (Figure 2G). Overall, Q23-APEX-GT2 effectively primed and expanded rare human PCT64^LMCA^ B cell precursors, facilitating germinal center formation, inducing V2-apex-targeted antibody titers, and promoting B cell maturation along favorable evolutionary pathways.

### Q23-APEX-GT2 trimer elicits V2-apex immunofocused antibody responses in outbred rhesus macaques

**Figure 3.**
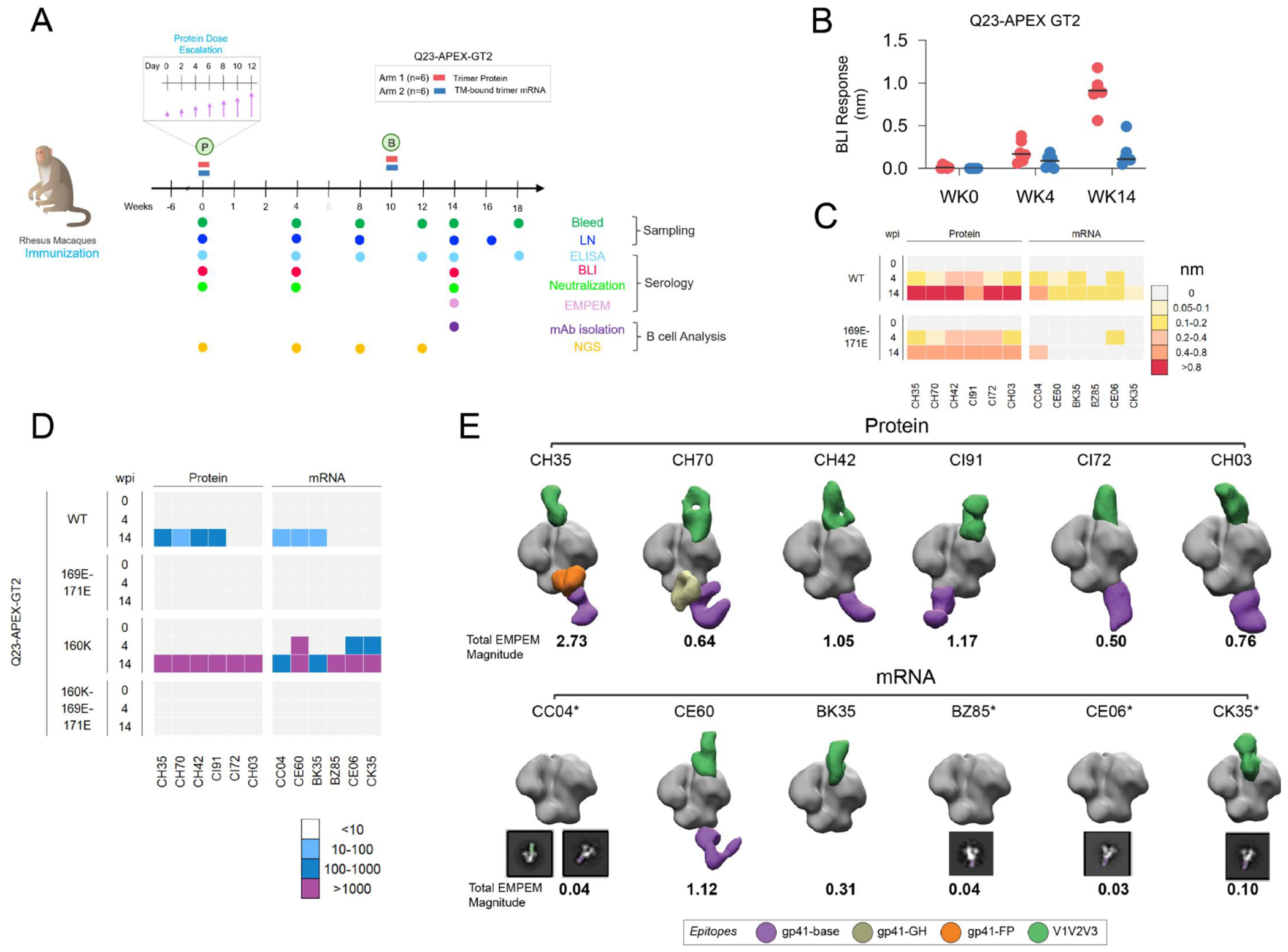
Q23-APEX-GT2 trimer immunization in rhesus macaques and analysis of serum antibody responses. See also Figures S5, S6 and S7. A. Schematic showing rhesus macaque immunization with Q23-APEX-GT2 trimer protein + SMNP (Arm 1) and membrane-anchored mRNA LNPs (Arm 2). Rhesus macaques were primed at week 0 with 100 μg Q23-APEX-GT2 protein + SMNP adjuvant (Arm 1) or 100 μg Q23-APEX-GT2 mRNA LNPs (Arm 2) administered subcutaneously and distributed into four injection sites (25 μg each). The Arm 1 protein priming immunization was given as escalating dose (DE) over 2 weeks and a single bolus dose for mRNA arm. Both groups were bolus boosted with 100 μg of protein + SMNP (Arm 1) or mRNA LNPs (Arm 2). Serum, LN, and PBMC sampling longitudinally, as well as functional analyses, indicated by colored circles. B. Binding of polyclonal serum IgGs to Q23-APEX-GT2 trimer antigen by BioLayer Interferometry (BLI). Protein group is in red, mRNA group is in blue. The antigen specific antibody titers were detectable at week 4 post-prime in both groups and were boosted after week 1o boost. Stronger antibody titers were detected in the protein compared to the mRNA group. C. Heatmap showing binding of longitudinal polyclonal serum IgGs (weeks, 0, 4 and 14) to Q23-APEX-GT2 and its V2-apex epitope knockout (R169E-K171E) showing binding dependence on strand C. D. Heatmap showing serum neutralization (Inhibitory Dilution 50, ID50) of pseudovirus bearing the Q23-APEX-GT2 mutations (132R-158T) and several V2-apex bnAb epitope knockout virus variants. Neutralization is dependent on strand C residues as evident by loss of neutralization with 169E-171E variant. Increased neutralization by immune sera was observed upon160K glycan knockout and this was completely abrogated on strand C epitope knockout in the N160K backbone (160K-169E-171E), further highlighting strand C-dependent neutralization. E. Electron microscopy polyclonal epitope mapping (EMPEM) imaging of immune serum Fabs from Q23-APEX-GT2-immunized RMs highlights immunofocused response in all protein immunized animals and 3/6 mRNA immunized animals to Q23-APEXGT2 trimer. Composites of Three-dimensional (3D) reconstructions generated from the negative stain electron microscopy images of polyclonal serum antibody Fabs bound to Q23-APEXGT2 trimer are segmented. Fabs are colored based on targeted epitope region. Colored 2D classes are shown when too few particles were present for 3D reconstruction. Total EMPEM magnitude for each dataset is listed and described in more details in Methods. An asterisk denotes animals in which the observed EMPEM binding responses, represented as total EMPEM magnitude, were visible in 2D classes only (≤0.1).

The rhesus macaque model provides a more competitive B cell environment in an outbred setting, making it a more physiologically relevant system to evaluate the ability of Q23-APEX-GT2 to prime and mature rare V2-apex-targeting B cell precursors. To assess the immunogenicity of the Q23-APEX-GT2 trimer in RMs, two groups (6 animals per group) were subcutaneously immunized with either 100 μg of trimer protein plus saponin/MPLA nanoparticle adjuvant (SMNP) ^68^ using a slow-delivery escalating-dose protocol (Arm-1) ^69,70^, or 100 μg of trimer mRNA lipid nanoparticles (LNPs) via bolus injection (Arm-2) (Figure 3A). Both groups also received a bolus boost of 100 ug of the same immunogen at week 10. Plasma samples were collected at various time points, and excisional lymph node biopsies were performed at weeks, −6, 4, 8, 12, and 14 or 16 to monitor antibody and B cell responses.

Post-prime immune sera showed Q23-APEX-GT2-specific antibody responses, as measured by ELISA and BLI, with substantial increases after the boost at week 10 (Figure 3B, S4A–D). Higher antibody titers were observed in the adjuvanted protein group compared to the mRNA group (~5-fold difference at WK14) (Figure 3B, S4A). Longitudinal serum antibody responses were further assessed for V2-apex epitope targeting by testing binding to strand-C residue substitutions (R169E-K171E (double knock-out – dKO)), which abolish binding of most V2-apex bnAbs ^28^, and binding to a glycan knockout at position 160 (N160K) on the Q23-APEX-GT2 trimer (Figure 3C, S4A–D). Positively charged V2-strand-C residues and the N160 glycan form the core binding region of the V2-apex bnAb site ^28^. Substitution of the V2-strand-C positively charged residues with negatively charged residues substantially reduced binding by polyclonal sera (1.1 - 1.7-fold reduction for protein, complete abrogation for mRNA) in both protein- and mRNA-immunized groups, while responses against the N160 glycan KO variant were slightly enhanced (1.1 - 1.9-fold enhancement for mRNA) (Figure 3C, S4). Overall, the protein group induced stronger responses than mRNA, with evidence of targeting the strand C of the V2-apex bnAb site.

We also tested longitudinal serum neutralization against the immunogen-matched Q23-APEX-GT2 pseudovirus and its strand C dKO variant. 4 of 6 animals in the protein group and 3 of 6 in the mRNA group neutralized the autologous virus (40 - 430 ID50 range for protein, 10 - 75 ID50 for mRNA) in a V2-apex epitope-dependent manner (Figure 3D, S4E). Consistent with binding data, mRNA-immunized animals had weaker nAb titers than the protein group (~4.5-fold higher in protein group) (Figure 3D, S4E). We also tested neutralization of immune sera using the non-germline-targeting wild-type (WT) Q23.17 tier-2 virus and its strand C- and N160 glycan-KO variants. Immune sera from one of the protein-group animals, CH35 showed weak but detectable neutralization of the WT Q23 virus (Figure S4E). Notably, all animals in both groups developed high nAb titers (ID50 range: Protein, >21870 and mRNA, 260 - >21870) against the N160-deficient Q23.17 virus, with substantial increases following the week-10 boost, as detected in week-14 sera (Figure S4E). These responses targeted the V2-apex strand C residues, as shown by the complete loss of neutralization against the N160-K169-K171 variant (Figure S4E). No heterologous virus neutralization was observed (Figure S4E). The results indicate that the Q23-APEX-GT2 trimer induces immunofocused polyclonal neutralizing antibody responses targeting the V2-apex bnAb site in outbred rhesus macaques.

To gain more insight into the epitopes targeted by polyclonal antibodies at week 14, we used electron microscopy polyclonal epitope mapping (EMPEM) ^71^. Antigen-binding fragments (Fabs) from the immune plasma of all 12 animals (6 from each group) were complexed with the Q23-APEX-GT2 trimer (Figure 3E, S5). Consistent with serum binding and neutralization data, EMPEM revealed that polyclonal antibody responses predominantly targeted the canonical V2-apex bnAb site in 6 of 6 protein-immunized animals and 3 of 6 mRNA-immunized animals (Figure 3E, S5). While the polyclonal antibodies overlap with the V2-apex epitope, negative stain resolution suggests that they primarily contact a single gp120 subunit due to their binding angles, with fewer interpretable quaternary interactions compared to canonical bnAbs. As expected, targeting of the trimer base was observed in the protein group but was largely absent in the mRNA group (Figure 3E, S5). The total EMPEM magnitude (average number of Fabs per trimer) was higher in the protein group compared to mRNA group (mean value of 1.14 for protein group and 0.27 for mRNA group; see also Methods and Figure 3E), which is consistent with the antibody titer results (Figure 3B and S4A–D). These results support the above data to suggest that the Q23-APEX-GT2 trimer elicits V2-apex-focused antibody responses in outbred macaques.

### Q23-APEX-GT2 enriches long CDRH3 V2-apex bnAb site specific B cells

**Figure 4.**
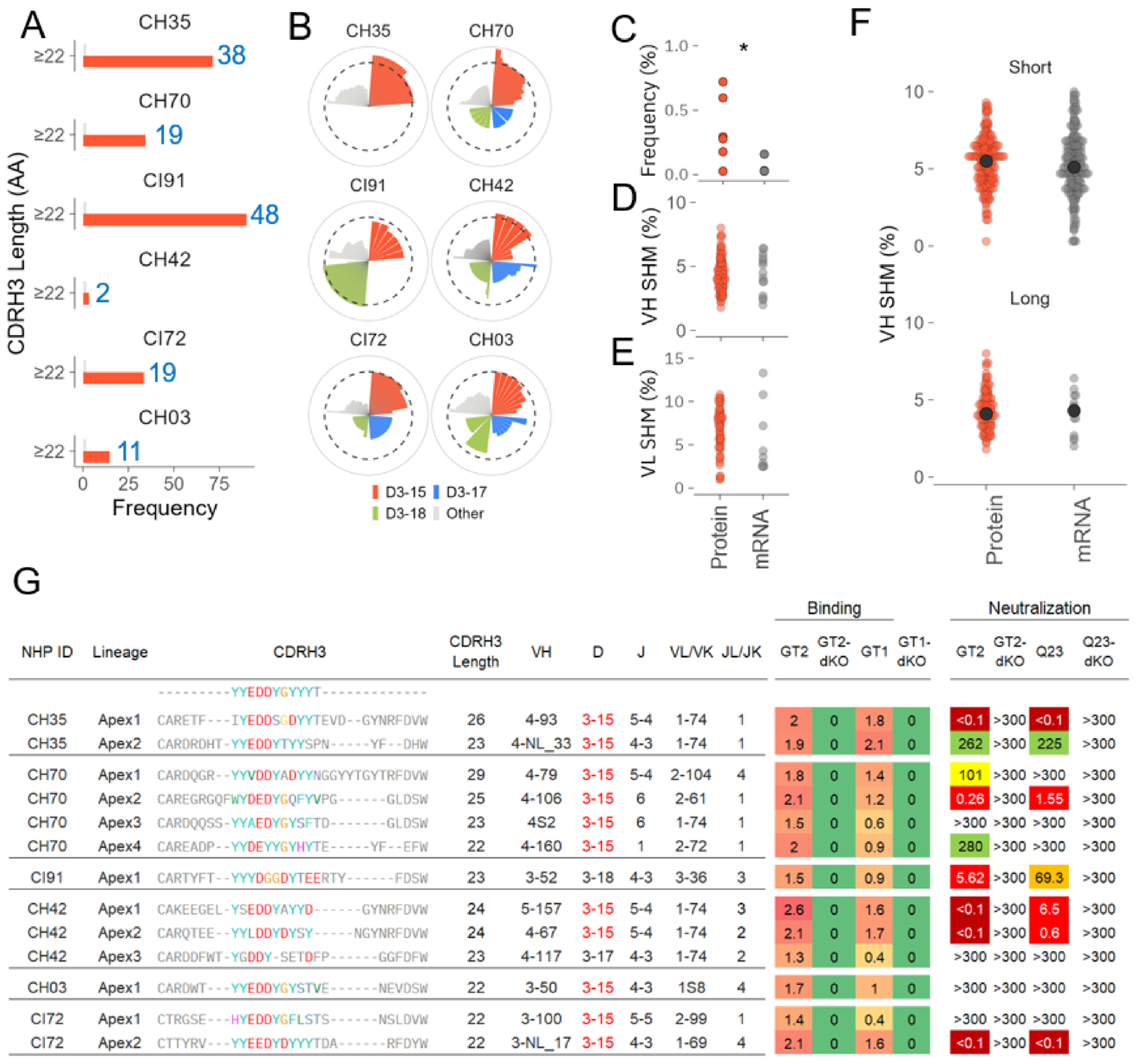
Isolation and functional characterization of Q23-APEX-GT2 trimer elicited rhesus V2-apex mAbs: immunogenetic features and epitope properties. See also Figures S8 and S9. A. Enrichment of Q23-APEX-GT2 V2-apex epitope sorted B cells from week 14 or 16 LNs of Q23-APEX-GT2 trimer protein immunized RMs. Bar plots show enrichment of long CDRH3s (length ≥22 AA) in protein-immunized animals in red; gray bar shows the frequency in naïve RMs (as a baseline reference). B. Circular bar plots showing the germline-D-gene usage in Q23-APEX-GT2 V2-apex epitope sorted B cells from panel A. IGHD3-15, IGHD3-17 and IGHD3-18 are colored while all other D-genes are combined as “others”. The dashed inner circle represents the CDRH3 length cutoff of ≥22 amino acids. C. Distribution of long CDRH3 B-cells (≥22AA) from Q23-APEX-GT2 V2-apex epitope sorted B cells in protein and mRNA immunized rhesus macaques. A higher fraction of long CDRH3 B cells was observed in protein-immunized rhesus macaques. D. Somatic hypermutation in the heavy chain (HC) variable region of site-specific monoclonal antibodies (mAbs) from Q23-APEX-GT2 protein and mRNA immunized rhesus macaques, regardless of CDRH3 length. E. Somatic hypermutation percentages in light chains (LC) of long CDRH3 (≥22 AA) mAbs from Q23-APEX-GT2 V2-apex epitope sorted B cells. F. Somatic hypermutation percentages in short (<22 AA) and long (≥22AA) CDRH3 mAbs from Q23-APEX-GT2 V2-apex epitope sorted B cells in protein and mRNA immunized animals. G. CDRH3 sequence, length and gene assignments for V, D, J for heavy chain and V, J for light chains from representative long CDRH3 mAbs lineages from Q23-APEX-GT2 protein immunized rhesus macaques. Germline IGHD3-15 D-gene is shown in red and the germline encoded anionic CDRH3 residues are highlighted in color. From each animal, representative sequences from each expanded lineage are shown. For each mAb, maximum BLI binding responses and IC50 neutralizations are shown with Q23-APEX-GT1 and Q23-APEX-GT2 trimer proteins and their corresponding virus and the strand C dKO variants and the WT Q23.17 (for Q23-APEX-GT1). All mAbs are dependent on V2-apex stand C core epitope.

To determine whether the Q23-APEX-GT2 trimer can prime V2-apex-targeting rare bnAb B cell precursors with long CDRH3 loops in outbred monkeys. To assess this, we used flow cytometry to isolate V2-apex-specific, IgG class-switched B cells from the lymph nodes (LNs) of six protein-vaccinated monkeys at week 14 or 16 (four or six weeks after the second homologous boost immunization). To isolate epitope-specific B cells, we used Q23-APEX-GT2 trimer and dKO probes (Q23-APEX-GT2+/Q23-APEX-GT2-dKO-ve) as baits (Figure S6A), with antibody sequences obtained by sequencing the amplified heavy and light chains from antigen-sorted single B cells (Supplementary Table S2 and Supplementary Table S3). Analysis of the week 14/16 lymph nodes showed that all 6 Q23-APEX-GT2 protein-vaccinated animals developed antigen-specific B cell responses (median = ~3%), with a sizable fraction being V2-apex specific (median = ~2.5%) (Figure S8B). We observed a strong enrichment of ≥22 amino acids CDRH3 loops (2- to 48-fold enrichment) in the V2-apex-specific B cells compared to the baseline naive B cell repertoire in RMs (Figure 4A), in all 6 vaccinated monkeys. These results demonstrate that Q23-APEX-GT2 immunization substantially shifted the distribution of B cells with long CDRH3s (a signature feature for V2-apex bnAbs) in outbred RMs. The immunogenetic analysis of the isolated antibodies revealed that three major germline D genes, IGHD3-15, IGHD3-17, and IGHD3-18, contributed to the long CDRH3 mAbs, with IGHD3-15 being used in 5 of the 6 protein immunized monkeys (Figure 4B). This preferential usage of the IGHD3-15 germline D gene in long CDRH3 V2-apex bnAbs is consistent with previous observations in rhesus bnAbs isolated from SHIV-infected monkeys ^58^.

To compare the B cell responses of the protein and mRNA group animals, we used the same workflow to isolate V2-apex site-specific single B cells from week 14/16 LNs of six Q23-APEX-GT2 trimer mRNA-vaccinated group animals (Supplementary Table S2 and Supplementary Table S3). Consistent with the serological data, the percentage of antigen- and epitope-specific B cells was lower in the mRNA group compared to the protein group (Figure S6B). Somatic hypermutation (SHM) analysis showed modest nucleotide divergence from the respective germline V genes in both the heavy (median = ~4.5%) and light (median = 3.5%) chains for protein group and relatively lower for the mRNA group (Figure 4D), which is consistent with the early stages of affinity maturation of these B cell responses. Immunogenetic analysis of the isolated antibodies showed IGHD3-15 solely contributed to the long CDRH3 mAbs in mRNA immunized animals (Figure S6C). Overall, protein immunized animals had superior antibody responses compared to the mRNA immunized group, which could have been due to the dose escalation prime regimen, the adjuvant and/or antigen display or suboptimal responses to the mRNA/LNPs when given subcutaneously rather than intramuscularly. We therefore focused on characterizing B cell and antibody responses in the protein immunized animals.

### Q23-APEX-GT2 induces long CDRH3 V2-apex bnAbs

We identified one to four long CDRH3 loop (22AA or longer) prototypic V2-apex bnAb-like lineages in week 14 or 16 LNs from each of the six Q23-APEX-GT2 trimer protein-vaccinated monkeys (Figure 4F). Since we only focused on week 14/16 LNs, it is conceivable that additional long CDRH3 lineages were present at the same or different time points. Each of the isolated lineages possessed D-gene (IGHD3-15, IGHD3-17 or IGHD3-18)-encoded anionic residues and predicted sulfated tyrosine motifs (e.g., EDDY, DDY, DDYDY, YDED, YEDD, DYY, YYD) in their CDRH3 paratopes (Figure 4G) known to enable interaction with the positively charged V2-apex core bnAb epitope ^28,56–58^. These long CDRH3 B cells utilized a diverse set of heavy and light chain V and J genes, suggesting that the germline D-gene encoded motifs were likely the primary drivers of antigen-specific B cell selection.

Epitope mapping using BLI and virus neutralization showed that the long CDRH3 lineages bound to the Q23-APEX-GT2 trimer in an epitope-specific manner (Figure 4G and S6D, Supplementary Table S4). These mAbs also bound the WT Q23.17 trimer (Q23-APEX-GT1), demonstrating their ability to recognize native-like Env trimer configurations associated with the virus. Recognizing and maturing bnAb UCA precursors toward native-like trimer configurations is considered a key step in guiding bnAb precursors along the bnAb maturation pathway ^37,72^. The mAbs exhibited trimer-dependent binding (with no binding to monomeric Q23-APEX-GT2 gp120 (Figure S6D) and showed variable dependence on the N160 glycan (Figure S6D). While binding decreased for some mAbs with the N160-deficient Q23-APEX-GT2 trimer, others showed enhanced or unchanged binding (Figure S6D). Of note, many of these mAbs neutralized not only the autologous Q23-APEX-GT2-matched pseudovirus but also the WT Q23.17 virus in an epitope-dependent manner (Figure 4G).

**Figure 5.**
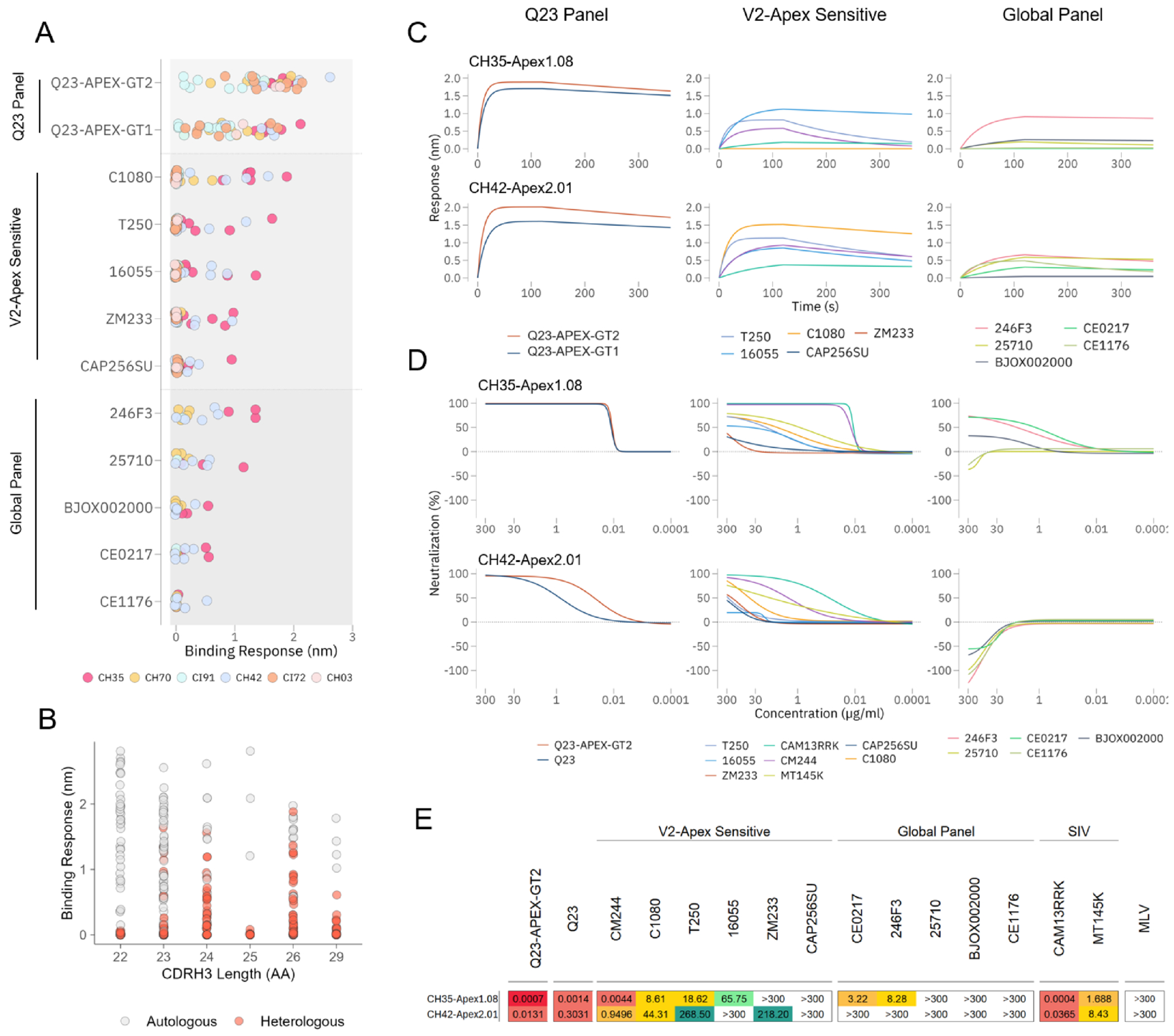
Q23-APEX-GT2 trimer elicited rhesus V2-apex mAbs exhibit cross-reactive binding with heterologous HIV trimers and modest neutralization breadth. See also Figure S8. A. Scatter plot illustrating BLI binding responses of all isolated mAbs to autologous (Q23-APEX-GT2 and Q23-APEX-GT1) HIV Envelope trimers, V2-apex neutralization-sensitive heterologous trimers (C1080, T250, 16055, ZM233, and CAP256.SU), and trimers from the global HIV-1 reference panel (246F3, 25710, BJOX002000, CE0217, and CE1176). Many Q23-APEX-GT2-elicited long CDRH3 rhesus V2-apex mAbs exhibited cross-reactive binding to diverse heterologous HIV trimers. B. BLI binding of isolated mAbs against autologous Q23-APEX-GT2 and heterologous HIV trimers from figure 5A. Each dot represents an antigen with heterologous binding only seen with mAbs of CDRH3 length 23AA and longer. C-D. BLI binding curves (C) and neutralization curves (D) of two representative mAbs, CH35-Apex1.08 and CH42-Apex2.01, isolated from protein-immunized rhesus macaques CH35 and CH42. These mAbs demonstrated binding and moderate neutralization breadth against the autologous Q23 panel, heterologous V2-apex-sensitive trimers, and the global HIV panel. Notably, both mAbs also neutralized chimpanzee Env-derived viruses, CAM13RRK and MT145K, which share the V2-apex bnAb site with HIV-1. E. Summary of neutralization IC50 values for V2-apex mAbs tested in panel B. Notably, CH35-Apex1.08 neutralized eight heterologous viruses, some with potent activity.

We next investigated the BLI binding and neutralization of the long CDRH3 loop mAbs against multi-subtype tier-2 globally representative HIV-1 virus panels ^43,73^. Multiple mAbs from four monkeys exhibited cross-reactive binding while CH35-Apex1.08 and CH42-Apex2.01 also neutralized up to 8 heterologous tier-2 viruses including two chimpanzee SIV viruses that share V2-apex bnAb site with HIV-1 but are evolutionarily more distant than group M viruses (Figure 5A–D, Supplementary Table S4) ^40,74^. These results demonstrate induction of authentic V2-apex bnAb precursors and their partial maturation into cross-neutralizing antibodies in an outbred macaque model using a germline-targeting immunogen. Thus, Q23-APEX-GT2 trimer immunization not only primed the authentic V2-apex bnAb precursors but also partially matured them along desired pathways for bnAb development.

An important question for targeted B cell approaches is what CDRH3 length and paratope features qualify as a bona fide V2-apex bnAb precursor. We observed that all B cells with CDRH3s of 22AA or longer bound as well as neutralized WT Q23 in a V2-apex bnAb epitope specific manner. However, while nearly all 23AA CDRH3 antibodies bound heterologous HIV trimers, the 22AA CDRH3-bearing V2-apex-targeted antibodies did not (Figure 5B, Supplementary Table S4). This pattern extended to neutralization, with only V2-apex-targeted antibodies with CDRH3s of 23AA or longer showing cross-neutralization of heterologous viruses. These findings suggest that a 23AA or longer CDRH3 length may be optimal for a *bona-fide* V2-apex bnAb precursor, but a larger dataset is required to draw a firmer conclusion ^57,58^. Whether 21AA or shorter CDRH3s can affinity mature into canonical V2-apex bnAbs remains to be investigated.

Based on these observations, we conclude that a *bona fide* V2-apex bnAb B cell precursor must meet the following criteria: i) a 22AA long CDRH3 loop with appropriate paratope features, ii) strand C epitope-dependent binding, iii) binding to both autologous WT and V2-apex-sensitive heterologous trimers, and iv) potential for autologous or heterologous virus neutralization. The last criterion (neutralization) may be a high bar, as these B cells are still early in their development with limited somatic mutations. Nevertheless, we believe that as *bona fide* bnAb precursors accumulate SHMs through vaccination, they should exhibit detectable neutralization of V2-apex-sensitive heterologous trimers, which would help differentiate them from strainspecific or non-bnAb B cell precursors.

Overall, the B cell analysis was consistent with polyclonal plasma analysis, with both showing reproducible targeting of the V2-apex bnAb site. Thus, our findings provide proof-of-concept that authentic bnAb precursors can be primed using a germline-targeting vaccination approach and that some breadth can be gained without a complex prime-boost immunization protocol

### Structural analysis reveals shared features of elicited rhesus and human V2-apex bnAbs

**Figure 6.**
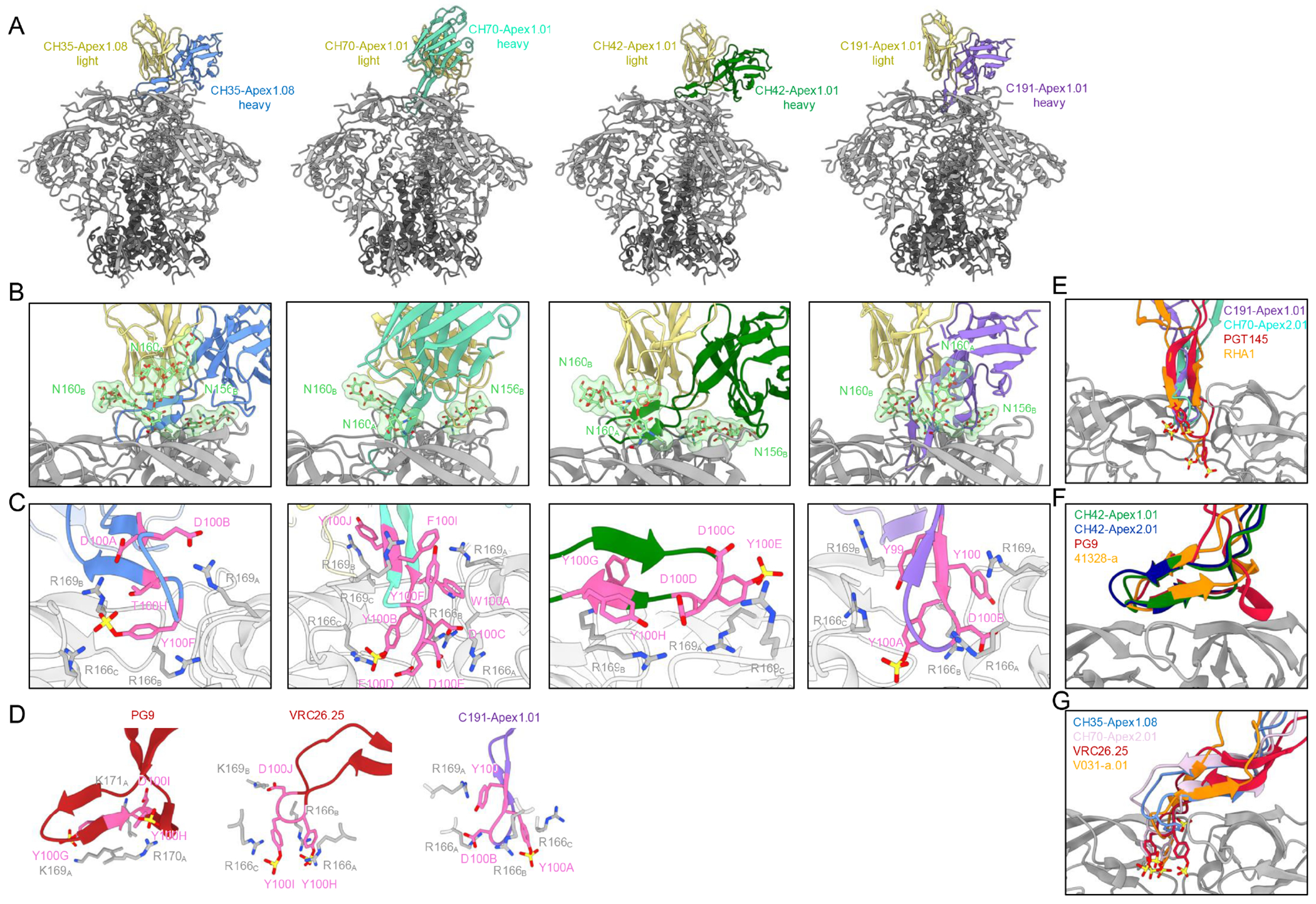
Cryo-EM structures of Q23-APEX-GT2-elicited antibodies reveal mimicry of canonical V2-apex bnAb recognition modes. See also Figures S9, S10, S11, and Dataset S1. A. Overall structure of Fab:envelope trimer complexes of V2-apex-targeted antibody lineages from four different Q23-APEX-GT2 immunized macaques determined by single-particle cryo-EM. All light chains are colored yellow while each heavy chain is colored by lineage. Envelope gp120 is shown in light gray and gp41 in dark gray. B. Expanded interface view of panel (A) to highlight Fab binding orientation and interactions with apical envelope glycans. Recognized glycans are shown in stick representation and colored green with transparent surfaces. Fab heavy and light chains are colored similarly to panel (A). C. Critical V2-apex gp120 contacts by HCDR3 germline-encoded D-gene residues. Fab heavy chains are colored by lineage similarly to panel (A), with paratope residues that are germline-encoded or conservatively mutated—somatic mutations that preserve the biochemical properties of the original germline residue—colored pink, shown in stick representation, and labeled by Kabat numbering. Sulfur atoms from sulfated tyrosine residues are colored yellow; nitrogen atoms are colored blue; and oxygen atoms are colored red. D. Structure and function of the D-gene germline-encoded three-residue YYD motif in human (PG9 and VRC26.25) and rhesus (CI91-Apex1.01) lineages. Fab HCDR3s from their respective complex structures are colored red (human) or purple (rhesus CI91), with the YYD motif colored pink, shown in stick representation, and labeled by Kabat numbering. Envelope V2 residues contacted by each respective YYD motif are shown with stick representation in isolation from the remainder of the gp120 structure. E-G. Structural superimpositions of Q23-APEX-GT2 vaccine-elicited V2-apex antibodies with mature human and rhesus V2-apex bnAbs using “combined-mode” VRC26-like (E), “needle” PGT145-like (F), and “axe” PG9-like (G) modes of recognition. Each structure is aligned by gp120 to compare V2-apex-bound Fab HCDR3 conformations and orientations. Mature human bnAb lineages are colored red and mature rhesus bnAb lineages are colored orange, with the vaccine-elicited antibodies colored similarly to panel (A).

Previous studies have shown three reproducible modes of CDRH3-dominated V2-apex epitope recognition: insertion of an extended “needle-like” CDRH3 directly into the trimer apex hole along the 3-fold axis ^41,55–58,60^; parallel or antiparallel main-chain hydrogen-bonding with the C-strand from a single protomer via an “axe-like” CDRH3 ^41,53,54^; and a “combined-mode” CDRH3 that simultaneously engages the C-strand through main-chain bonding and inserts the loop tip into the trimer hole ^56,75^. We have previously defined lineages utilizing these modes of V2-apex recognition as members of an “extended-class,” which are antibodies that utilize structurally similar paratopes derived from unique immunogenetic origins ^41,58^. Thus, we would propose that the one important criterion to define a *bona fide* V2-apex bnAb B cell precursor is: belonging to an extended-class characterized by one of three canonical modes of V2-apex bnAb recognition. To investigate whether the immunogenic and phenotypic properties of the Q23-APEX-GT2-elicited antibodies in fact recapitulated V2-apex bnAb recognition at the molecular level, we thus used single-particle cryo-EM to determine the structures of Fabs from six lineages (from four macaques: CH35, CH70, CH42 and CI91) in complex with Q23-APEX-GT2 trimer (Figure 6, S6E–G, Dataset S1).

The cryo-EM structure of the CH35 macaque mAb CH35-Apex1.08 in complex with the Q23-APEX-GT2 trimer was resolved at 3.2 Å resolution. The structure revealed a 1:1 Fab:trimer stoichiometry with asymmetric binding along the 3-fold trimer axis (Figure 6A). CH35-Apex1.08 inserted between the N156 and N160 glycans on a single protomer while also engaging the N160 glycan from an adjacent protomer, leading to 52% of the total binding surface being mediated by apical glycans (Figure 6B, S6E). CH35-Apex1.08 utilized a combined-mode CDRH3 topology, similar to bnAbs VRC26.25 (human) and V031-a.01 (macaque) ^56,58^. It engaged the C-strand through three parallel main-chain hydrogen bonds while inserting an anionic loop tip—containing a sulfated tyrosine—into the trimer hole (Figure 6C, G, S6F) ^13,28,53–55,57^. This tyrosine sulfation, a common posttranslational modification in V2-apex bnAbs, facilitated interactions with core V2-apex residues R166, R168, and R169 from one or more Env protomers. Additionally, K171 on protomer B (K171_B_) was positioned between CDRL1 and CDRL3. Notably, IGHD3-15 D-gene germline-encoded residues played a key role in stabilizing interactions across all three Env protomers. D100A and D100B formed electrostatic interactions with residue R169 from promoter A (R169_A_) and residue R169 from promotor B (R169_B_), while sulfated Y100F engaged R169_A_ and residue R166 from promotor C (R166_C_) via electrostatic interactions and R166_B_ through cation-π interactions. The methyl group of T100H further stabilized binding by engaging the aliphatic chain of R169_B_. Unlike human bnAb VRC26.25, which inserts a second sulfated tyrosine into the trimer hole, rhesus bnAbs often substitute it with an anionic residue ^58^. In line with this, CH35-Apex1.08 acquired a D100G somatic mutation (Tyr to Asp), allowing electrostatic contacts with R166_A_ and R169_C_, effectively completing recognition of the cationic lining of the trimer hole (Figure S6G).

The cryo-EM structures of the CH70 macaque mAbs CH70-Apex2.01 and CH70-Apex1.01, resolved at 3.8 Å and 3.0 Å respectively, revealed distinct modes of V2-apex recognition (Figure 6A, S6E–G). CH70-Apex2.01 bound near the trimer 3-fold axis with a 1:1 Fab:trimer stoichiometry, engaging three apical glycans, though these interactions accounted for only 18% of the total binding surface (Figure 6B, S6H). The antibody primarily engaged Env through an extended, needle-like CDRH3 that inserted directly into the trimer hole, resembling the recognition mode of bnAbs PGT145 (human) and RHA1 (macaque) (Figure 6C, E) ^55,57^. Extensive contacts with core V2-apex residues were mediated by paratope residues that were either IGHD3-15 germline-encoded or conservatively mutated—somatic mutations that preserve the biochemical properties of the original germline residue (e.g., Asp to Glu maintaining negative charge or Tyr to Trp retaining aromaticity) (Figure 6C). Aromatic residues Y100F and F100I recognized R169_C_ via hydrogen bonding and cation-π interactions, while W100A and Y100J engaged R169_A_ and R169_B_ from other protomers. Further down into the trimer hole, the anionic CDRH3 tip—comprising sulfated Y100B, D100C, E100D, and D100E—formed salt bridges with R166 from all three protomers. Antibody CH70-Apex1.01 also bound Env with a 1:1 Fab:trimer stoichiometry but approached the three-fold axis at a more asymmetric angle (Figure S6E–G). Similar to CH70-Apex2.01, it engaged three apical glycans but contributed a greater fraction (36%) of the total binding surface (Figure S6H). CH70-Apex1.01 utilized a combined-mode CDRH3 topology, inserting two germline-encoded sulfated tyrosines into the trimer hole while simultaneously forming three main-chain hydrogen bonds with the C-strand (Figure 6G, S6J). Similar to human bnAb VRC26.25, these sulfated tyrosines formed electrostatic contacts with R166 from all three Env protomers. Additional interactions included D100C forming a salt bridge with R169_C_ and Y100D stabilizing the extended aliphatic chain of R169_A_ (Figure S6J, left).

The cryo-EM structures of CH42 macaque mAbs CH42-Apex1.01 and CH42-Apex2.01, resolved at 2.9 Å and 3.1 Å respectively, showed both antibodies binding with 1:1 Fab:trimer stoichiometry through an axe-like CDRH3, which formed parallel strand bonding with the C-strand (Figure 6A, F, S6E–H). This recognition mode mirrors that of bnAbs PG9 (human) and 41328-a.01 (macaque) ^53,58^. CH42-Apex1.01 inserted between the N156 and N160 glycans on a single protomer while also engaging the N160 glycan from an adjacent protomer, resulting in 54% of the Fab binding surface being mediated by apical glycans (Figure 6B, S6H). While three parallel main-chain hydrogen bonds were formed with the C-strand of protomer B, the CDRH3 of CH42-Apex1.01 extended across the trimer hole, allowing IGHD3-15 germline-encoded residues to interact with core V2-apex residues from all three protomers (Figure 6C). Specifically, D100C and D100D were positioned near R169_C_ and R169_B_, respectively, suggesting potential electrostatic interactions. The sulfated Y100E formed a salt bridge with R169_C_ while simultaneously stabilizing the extended aliphatic chain of R169_A_. Additionally, Y100G engaged K168B via cation-π interactions, and Y100H interacted with R169_B_ through both aliphatic chain stabilization and hydrogen bonding with the N-terminal amine. Antibody CH42-Apex2.01 similarly bound asymmetrically to the trimer 3-fold axis, engaging three apical glycans that accounted for 46% of the Fab interactive surface (Figure S10, S6E–H). Its interactions included four main-chain hydrogen bonds with the C-strand, three through parallel strand bonding and one via N100I carboxyamide sidechain with K171 backbone amide. Notably, cryo-EM density revealed three posttranslational sulfation modifications on IGHD3-15 germline-encoded residues Y100D, Y100F, and Y100H. These modifications collectively facilitated electrostatic interactions with R169 from all three protomers and R166 from protomers A and C (Figure S6G, right). Additional contacts included salt bridges with R169_A_ (via D100B) and R166_A_ (via D100E), plus multiple light chain interactions with K171_B_ mediated by LCDR1 and LCDR3 (Figure S6K).

The cryo-EM structure of CI91 macaque mAb CI91-Apex1.01 complex was resolved at 3.0 Å, showing a 1:1 Fab:trimer stoichiometry with binding near the three-fold trimer axis (Figure 6A). Similar to other Q23-APEX-GT2-elicited antibodies, CI91-Apex1.01 recognized three apical glycans—N160 and N156 from one protomer and N160 from an adjacent protomer—accounting for 30% of the total interactive surface (Figure 6B, S6H). CI91-Apex1.01 primarily engaged Env through its CDRH3, which adopted an extended needle-like conformation with two sulfated tyrosines flanking the loop tip that inserted directly into the trimer apex hole. This binding mode closely resembled that of mature human and rhesus bnAbs in the PGT145-extended class (Figure 6E). CI91-Apex1.01 recognized R166 and R169 from all three protomers, with interactions largely mediated by IGHD3-18 D-gene germline-encoded residues forming the descending CDRH3 β-strand and part of the loop tip. Specifically, Y100 was positioned between R166_B_ and R169_A_, stabilizing their extended aliphatic chains. Sulfated Y100A engaged R166_B_ and R166_C_ through electrostatic interactions and also formed a hydrogen bond with the backbone amide of R166_B_. Additionally, D100B was inserted between R166_A_ and R166_B_ (Figure 6E). The second sulfated tyrosine, Y100F, played a critical role in the paratope by recognizing both R166_C_ and R169_B_ (Figure S6L). Structural alignment of CI91-Apex1.01 with CH70-Apex2.01, another needle-like V2-apex bnAb precursor, showed that both Q23-APEX-GT2-induced antibodies aligned their CDRH3s with those of mature bnAbs VRC26.25 and RHA1—prototypical human and rhesus bnAbs described in this extended class. However, unlike the mature bnAbs, CI91-Apex1.01 and CH70-Apex2.01 did not penetrate as deeply into the trimer hole (Figure 6E). Instead, the sulfated HCDR3 loop tips overlapped with the mature rhesus V2-apex bnAb 44715-a (Figure S6M) ^58^.

CI91-Apex1.01 was notable for its utilization of IGHD3-18 germline D-gene, in contrast to IGHD3-15, which has been consistently used by all other structurally characterized rhesus V2-apex bnAbs and their precursors, both in this study and previous reports ^57,58,76^. While IGHD3-15 encodes a rhesus-specific EDDY motif, IGHD3-18 encodes a three-residue YYD motif, which is also found in human IGHD3-3. This motif is shared by three human V2-apex bnAb lineages—PG9, PCT64, and VRC26.25—all of which originate from IGHD3-3 ^8,28,46,50^. The mature bnAbs PG9 and VRC26.25 retain the YYD motif to mediate electrostatic interactions with core V2-apex epitope residues, although through distinct structural mechanisms (Figure 6D). CI91-Apex1.01 represents a third, structurally divergent conformation of this conserved motif while maintaining its functional role in interacting with cationic V2-apex residues. Additionally, it retains the characteristic posttranslational sulfation modification on the IGHD3-3/IGHD3-18 germline-encoded tyrosines.

Notably, all vaccine-elicited rhesus antibodies recognized the engineered germline-bindingenhancing T132R modification via salt bridges mediated by one or two anionic residues, most commonly (5/6 lineages) through the light chain (Figure S6N). The result suggests that the T132R modification likely improved the vaccine priming efficacy of Q23-APEX-GT2 by selectively engaging V2-apex precursors through direct epitope-paratope interactions.

Overall, this structural analysis provides explicit molecular evidence for the ability of Q23-APEX-GT2 to induce bona fide V2-apex bnAb precursors in multiple outbred macaques and reveals how germline-encoded D-gene residues compose the paratope recognizing critical V2-apex contact residues, most commonly at positions 166 and 169. These precursors fell into previously defined bnAb extended-classes which recapitulated all three modes of mature V2-apex bnAb recognition, with two modes of recognition being observed even within a single macaque, demonstrating that Q23-APEX-GT2 can prime a diverse pool of precursors with distinct structural features. We did not observe clear differences between neutralizing and cross-neutralizing long CDRH3 antibodies elicited in our study. However, structural analysis revealed that the two broad neutralizers, CH35-Apex1.08 and CH42-Apex2.01, use a combined recognition mode involving C-strand mainchain hydrogen bonding along with CDRH3 loop insertion into the trimer apex “hole.” This feature, rather than affinity alone, appears to enable early heterologous neutralization and may serve as a precise structural template for the initial antibody response we aim to elicit through vaccination. These findings align with prior studies by Roark et al. ^58^ and Habib et al. ^64^, which describe two major maturation pathways for V2-apex antibody lineages: one dependent on the N160 glycan and another that initially bypasses it. Both pathways are productive, but antibodies that accommodate the N160 glycan ultimately achieve greater breadth and potency. Therefore, an ideal immunogen should elicit precursors capable of this combined recognition and guide their maturation toward N160-glycan accommodation. Encouragingly, our current immunogen elicits precursors with the potential to follow either pathway.

### Lineage tracing of long CDRH3 V2-apex bnAb B cell responses by bulk NGS

**Figure 7.**
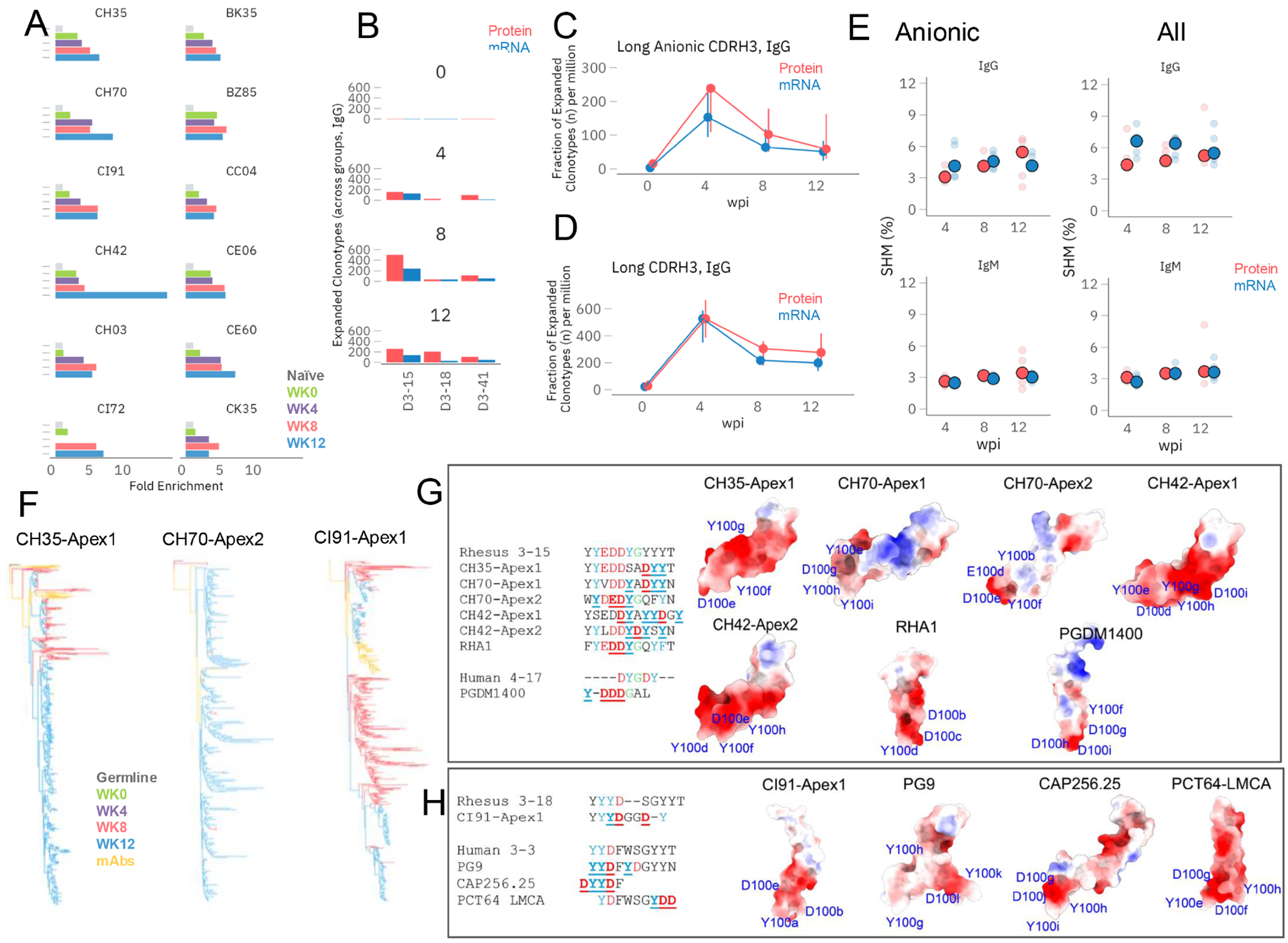
B cell lineage tracing of long CDRH3 V2-apex mAbs in Q23-APEX-GT2 trimer protein and mRNA immunized macaques by bulk Next-Generation Sequencing (NGS). See also Figures S12, S13 and S14. A. Longitudinal enrichment of long CDRH3 B-cells (≥22 AA) for individual RMs in Q23-APEX-GT2 protein and mRNA vaccinated animals. LN samples from time points, weeks 0, 4, 8 and 12 were analysed a and compared with baseline naïve macaque repertoires. B. Longitudinal enrichment of anionic D genes within the expanded clonotype IgG compartment. Protein is colored red, mRNA is colored blue. Certain D-genes show enrichment as the immunization progresses indicating antigen driven selection. C. Longitudinal enrichment of all long CDRH3s (≥22 AA) within expanded clonotypes in Q23-APEX-GT2 immunized animals. D. Longitudinal enrichment of anionic long CDRH3s (≥22 AA) within expanded clonotypes in Q23-APEX-GT2 immunized animals. E. Longitudinal increment in SHM percentages within the IgG compartment of Q23-APEX-GT2 trimer protein-immunized animals (top half) within B cells bearing anionic CDRH3s. F. Phylogenetic trees show longitudinal development V2-apex mAb lineage in animals CH35, CH70, and CI91. The week 14 isolated mAbs are clustered with B cell sequences from weeks 0, 4, 8, and 12 derived from bulk NGS. G-H. Two rhesus germline D-gene solutions (IGHD3-15 and IGHD3-18) share commonalities with human germline D-gene solutions (IGHD4-17 and IGHD3-3). Both incorporate anionic CDRH3 residues and sulfated tyrosine motifs, which are critical for V2-apex bnAb site recognition. While the rhesus IGHD3-15-encoded EDDY motif resembles the human IGHD4-17-encoded DY motif (G), the rhesus IGHD3-18-encoded YYD motif is identical to the human IGHD3-3-encoded YYD motif (H). Q23-APEX-GT2 successfully induced V2-apex bnAb precursors in rhesus macaques carrying either of these motifs. Alignments of CDRH3 amino acid sequences from Q23-APEX-GT2 vaccine-elicited V2-apex mAbs are compared with corresponding rhesus and human germline D-gene sequences, highlighting the anionic motifs involved in V2-apex epitope recognition. Structural analysis of the CDRH3 loops of these mAbs reveals that the apical positioning of this germline-encoded motif is essential for recognizing the V2-apex bnAb site.

To track the population dynamics of long CDRH3 (22 amino acids or longer, though 22AA long CDRH3 B cells showed limited heterologous binding) B cell responses following Q23-APEX-GT2 immunizations, as well as the expansion and longitudinal development of isolated V2-apex epitope-specific B cells, we performed next-generation sequencing (NGS) of lymph node B cells at weeks 0, 4, 8, and 12 using a bulk IgG and IgM immunoglobulin amplification approach ^64^. Prior to immunization, animals in both the protein and mRNA vaccine groups exhibited a typical Gaussian distribution of B cell CDRH3 loop lengths with a peak at 14AA (Figure S7A). Consistent with the rhesus baseline repertoire, a small fraction of long CDRH3 B cells (range: 0.18 - 0.68 %) was observed across all animals, predominantly of the IgM isotype (Figure 7A, S7B). Post-immunization, B cell repertoires were highly enriched for longer CDRH3s, with median lengths increasing from 14 amino acids (baseline in monkeys) to 16–18 amino acids. This shift was largely driven by B cells with CDRH3s of 22 amino acids or longer (Figure S7C–D). At week 4, a more rapid enrichment of long CDRH3 B cells was observed in the protein-vaccinated group compared to the mRNA group (Figure S13). However, by week 12 (two weeks post-second immunization), the two groups were comparable. The shift toward longer CDRH3s was primarily contributed by class-switched IgG B cells, a trend that was consistent across both vaccine groups (Figure S7C–D).

One limitation of bulk NGS is that each B cell can contribute multiple copies of the same Ig transcript, potentially skewing the analysis. This issue is particularly pronounced for plasmablasts or plasma B cells, which express very high numbers of Ig mRNA transcripts and could misrepresent the magnitude of B cell responses. To address this bias, we analyzed long CDRH3 loop IgG B cells focusing only on expanded clones (>3 clonal members). This approach revealed a similar trend of long CDRH3 B cell expansions, with both vaccination groups appearing comparable (Figure 7A). However, compared to the mRNA group, protein-vaccinated animals exhibited a substantially higher number of long CDRH3 B cell clonotypes with appropriate anionic paratope motifs characteristic of V2-apex bnAbs (Figure 7B–D), consistent with earlier findings indicating superior responses in the protein group. Long CDRH3s were highly enriched in IGHD3-15, but several other rhesus germline D3 genes, such as IGHD3-18 and IGHD3-41, also contributed to the expanded long CDRH3 lineages (Figure 7B, S7E–F).

We investigated the percentage of somatic hypermutation (SHM) in the VH region of IgM and IgG sequences at weeks 4, 8, and 12 post-immunizations (Figure 7E), which ranged from ~3-7% (Figure 7E). While SHM percentage in IgM B cells remained largely unchanged over time across both immunization groups, SHMs in class-switched IgG B cells, particularly in long CDRH3 B cells, increased over time in the protein-vaccinated group. We also observed the elevated prevalence of long CDRH3 B cells with sulfated tyrosine CDRH3-motifs (Figure S7G). These findings suggest that long CDRH3 B cell lineages were under strong antigen-driven selection, consistent with efficient priming of long CDRH3 bnAb B cell lineages.

To track the lineage development of the prototype V2-apex site-specific mAbs isolated at week 14 (shown in Figure 4F), we surveyed their corresponding lineages in the longitudinal NGS data. Most of these site-specific lineages were expanded, with a few exceptions (Figure 7F, S7H). NGS lineage tracing revealed that most prototype long CDRH3-expanded lineages were likely activated by the immunization prime (Figure S7H). These lineages became detectable by week 8 but were mostly not observed at the week 4 time point. This observation may have been influenced by B cell expansion size and trafficking, as the analysis was based on LN biopsies, with different LNs sampled at various time points. Overall, the data demonstrate that the Q23-APEX-GT2 trimer efficiently primed rare prototype V2-apex long CDRH3 bnAb B cells after the initial vaccination and subsequent homologous boost, in most cases, facilitated robust expansion and further evolution of these bnAb lineages.

NGS analysis revealed that, compared to the mRNA group, the protein group animal exhibited a greater number of prototype V2-apex-like expanded long CDRH3 lineages. The magnitude of these expansions substantially increased following the boost immunization, and we identified more than 10 long CDRH3 B cell lineages exhibiting V2-apex bnAb-like features in some animals. Overall, NGS identified a relatively larger number of expanded prototype V2-apex bnAb-like long CDRH3 B cell precursor clones compared to those found through antigen epitope-specific mAb isolation. To determine whether pre-existing IgM or IgG V2-apex-like long CDRH3 B cell precursor frequencies influenced the elicitation and expansion of these lineages, we analyzed pre-bleed lymph node LN NGS data. The analysis revealed comparable frequency of IgM- and IgG-encoded long CDRH3 V2-apex bnAb-like B cell precursors across all animals (Figure S7G), suggesting that pre-existing precursors had minimal influence on the observed differences across animals.

### Rhesus V2-apex long CDRH3 B cell repertoires: lessons for human vaccination

To gain deeper molecular insight into the Q23-APEX-GT2-elicited rhesus V2-apex bnAbs and their relevance to targeted human vaccine strategies, we closely examined the germline D-gene–encoded paratope motifs. Our analysis revealed that, while Q23-APEX-GT2 efficiently engages rhesus IGHD3-15–encoded bnAb lineages bearing the CDRH3 EDDY motif, it also successfully engages a IGHD3-18–encoded bnAb in one rhesus macaque (CI91), which incorporates the YYD motif (Figure 7G, H). This finding is distinctive to our study, as all previously reported rhesus V2-apex bnAbs are encoded exclusively by the IGHD3-15 gene ^57,58^. This underscores the potential of Q23-APEX-GT2 to engage diverse but convergent epitoperecognizing paratope solutions. The rhesus IGHD3-15–encoded EDDY paratope closely mirrors the human IGHD4-17–encoded DY motif found in one of the most potent and broadly neutralizing human bnAb prototypes, PGDM1400 ^77^. In contrast, the rhesus IGHD3-18–encoded YYD paratope resembles the human IGHD3-3-encoded YYD motif observed in three human V2-apex bnAb prototypes: PG9, CAP256, and PCT64 ^8,28,46,50^. While a variety of D-gene–encoded CDRH3 anionic motifs are present in both infection- and vaccine-elicited human and rhesus V2-apex bnAb prototypes, the apical positioning of the CDRH3 motif—allowing interaction with the buried, positively charged strand C - is critical for binding, as supported by structural studies (Figure 6E – G, Figure 7H).

To assess the relevance of V2-apex antibody germline features observed in rhesus macaques for human vaccine strategies, we analyzed the frequency of key bnAb-encoding CDRH3 motifs in the human naïve B cell repertoire ^62^. The median occurrences (per million B cells) of these motifs at apical positions in long CDRH3s (≥22 amino acids) were: YYD (1174, range 750–2714), DY (981, range 607–1456), DDY (21, range 13–35), (E|D)(E|D)Y (47, range 26–96), and (E|D)DDY (11, range 4–19) (E|D – E or D) (Figure S7I–J, Supplementary Table S5). These data reveal a clear trend: as anionic motif complexity increases, its frequency in the human repertoire declines. Simple motifs like DY and YYD are common, whereas DDY is an order of magnitude rarer, and (E|D)DDY is the least frequent. This suggests that while the immunogenetic features engaged by Q23-APEX-GT2 immunization in macaques exist in humans, they are less prevalent. Their rarity implies that effective precursor priming strategies may be needed to expand and engage these bnAb B cell precursors. Overall, our study demonstrates that rhesus macaques are a useful model for inducing V2-apex bnAbs with features in common with humans, although there are potentially important differences.

## DISCUSSION

Molecular vaccine design offers significant promise in overcoming the challenges of inducing protective bnAbs against HIV ^18,21,24^. Our approach leverages molecular insights of HIV bnAbs to guide the development of immunogens that target specific bnAb precursors and direct their maturation. In this study, we applied antibody-guided structure-based design to develop a germline-targeted trimer immunogen targeting the V2-apex bnAb site, a key broadly neutralizing epitope on HIV Env. Immunization of outbred macaques with our engineered Q23-APEX-GT2 immunogen successfully expanded and partially matured HIV V2-apex bnAb precursors, demonstrating that cross-neutralizing antibodies can be induced by a germline-targeting immunogen in an outbred macaque model. These findings highlight the V2-apex as a particularly promising target for vaccination and suggest that bnAb induction at this site may be achievable including in humans through a relatively straightforward pathway.

Priming rare HIV bnAb precursors is a critical step in bnAb induction ^78–80^. The V2-apex bnAb site is a highly promising vaccine target recognized by some of the most potent and broad mAbs; however, priming its precursors remains challenging due to the rarity of long CDRH3 precursors with necessary structural and biochemical features ^13,28,61^. To address this, we developed a priming immunogen for the V2-apex bnAb site based on the Q23.17 HIV Env, which has consistently induced V2-apex bnAbs in rhesus macaques in a SHIV infection model ^58,64^. The Q23 GT-immunogen was engineered to engage diverse V2-apex bnAb precursors, to increase the likelihood of priming diverse rare bnAb B cells *in vivo*. Analyses of Q23 GT-trimer elicited polyclonal immune sera demonstrated strong antibody immunofocusing to the V2-apex site, with responses dependent on the N160 glycan and strand C region residues, key components of the V2-apex bnAb site. This strong antibody immunofocusing is noteworthy, given the complex antigenic landscape of the HIV glycoprotein surface, making the Q23-APEX-GT2 trimer an excellent candidate for priming bnAb precursors.

Antigen-specific B cell analysis revealed the induction of V2-apex-specific long CDRH3 antibodies that bound a genetically and antigenically diverse panel of HIV Envs. Despite using only a single homologous prime and boost immunization, some of these antibodies affinity matured to exhibit heterologous tier-2 HIV neutralization. The vaccine-induced neutralizing antibodies displayed structural and genetic features resembling human V2-apex bnAbs, encompassing all three known CDRH3 configurations ^58,81^. This study shows that GT-trimer immunization alone can successfully prime authentic V2-apex bnAb B cell precursors and drive their partial maturation into cross-neutralizing antibodies in outbred macaques. In contrast, germline-targeting efforts for other HIV Env sites have never achieved neutralization or even cross-reactive binding to native-like HIV trimers in outbred monkeys ^31,32^. One potential reason is that Q23-APEX-GT2 in our study was minimally modified at the V2-apex bnAb site, preserving a near-native trimer configuration while enhancing affinity for diverse prototype rhesus and human bnAb UCAs. This may enable affinity maturation to be driven by a native-like trimer, selectively shaping primed BCR configurations for bnAb development. This may define V2-apex bnAb germline-targeting strategies, where preserving the native-like trimer architecture at the bnAb epitope—despite engineering modifications—is essential for engaging long bnAb B cell precursors that rely on trimer-dependent recognition. Overall, the findings show that V2-apex bnAbs, which heavily rely on long CDRH3-encoded residues for epitope recognition, can mature into early bnAbs with minimal diversification in the boost regimen, a result that aligns with observations of vaccine-induced ultra-long CDRH3 bovine HIV bnAbs ^82,83^.

An important consideration in translating rhesus macaque-based V2-apex bnAb vaccine strategies to humans is the potential difference in germline D-gene features that affect the initial engagement of germline-targeting immunogens with bnAb B cell precursors. While rhesus and human V2-apex bnAbs share immunogenetic and structural similarities, a key distinction lies in their D-gene-encoded anionic motifs, which influence bnAb precursor recruitment and maturation. In rhesus, the IGHD3-15 germline D-gene encodes a highly anionic EDDY motif, facilitating electrostatic interactions with the positively charged V2-apex region of the HIV Env trimer. This feature is critical for early B cell engagement and subsequent affinity maturation toward bnAbs. The most common human V2-apex bnAb germline D-gene motif, found in prototypic bnAbs PG9, CAP256, and PCT64, is the IGHD3-3-encoded YYD motif, which can also originate from human germline D-genes, IGHD3-9, IGHD3-16, and IGHD3-22 ^28,62,84^. Additionally, human V2-apex bnAb precursors can encode rhesus-equivalent DY (encoded by IGHD4-17) or DDY (likely generated through secondary diversification) motifs. Compared to rhesus, all three human CDRH3-paratope solutions contain fewer anionic residues in their native configuration, which may limit their recruitment and evolution. However, the rhesus IGHD3-18 germline D-gene incorporates a YYD motif, and Q23-APEX-GT2 successfully engaged a bnAb precursor bearing this motif in our monkey trial. This observation suggests that immunogens designed to engage YYD-bearing rhesus bnAb precursors could expand the pool of potential bnAb-precursor lineages in humans, improving the translation of vaccine strategies to a broader class of human V2-apex bnAbs. Irrespective of the differences, the rhesus macaque offers the opportunity to test a full sequential immunization strategy – from precursor priming to bnAb maturation and polishing – and establish proof-of-principle for the strategy in an outbred animal model.

While our GT-trimer effectively activated V2-apex bnAb precursors, variability in priming efficiency across animals underscores the need for further optimization to consistently and robustly engage diverse precursors. We observed that GT-priming was sufficient to induce early cross-neutralizing antibodies with some neutralization breadth, necessitating boost strategies to enhance the breadth and potency of these antibodies. Q23 Env lineage-based boosts, with minimally engineered V2 C- strand, may effectively broaden V2-apex bnAb responses, as strand C diversity alone has been shown to be sufficient for V2-apex bnAb maturation in macaques and humans ^8,46,52,57,58,64^. This Env lineage-based approach is likely to reduce *de novo* B cell responses seen with heterologous boosts at secondary immunization ^85,86^, though it may face challenges from circulating antibody feedback effects ^87–89^. In our priming experiments, we did not observe extremely high antibody titers that may interfere with the boosting immunogens. Furthermore, it is important to note that heterologous boosts will reduce effects of antibody feedback ^87^, as the affinity of the existing antibodies will be reduced as a result of the epitope structural changes introduced in the immunogen. The optimal affinity distance between successive immunogens is also crucial for efficient bnAb memory B cell (MBC) recall and maturation ^26,34,52,90,91^. A key challenge for MBC recall is limited MBC participation in secondary GCs, often due to differentiation into plasma cells or recruitment of *de novo* primed naïve B cell responses during boosts ^92–94^. Therefore, in multi-stage HIV vaccination strategies, preserving the bnAb MBC pool at each immunization step is critical for their maturation and eventually differentiation into plasma cells at the final boost to induce durable antibody titers.

The overall goal of HIV vaccination is to elicit broad serum neutralization targeting multiple bnAb specificities. Our study demonstrates that bnAb induction through vaccination is feasible and provides a framework for targeting CDRH3 germline-encoded features—an approach that could be extended to other Env bnAb sites on the surface glycoproteins of highly evasive viruses. The V2-apex bnAb site is particularly suited for HIV vaccine targeting, offering a potentially simpler immunization solution compared to other Env bnAb sites. By overcoming challenges like V2-apex rare precursor activation and showing the potential of GT vaccines to coax the antibody response a considerable distance along a desirable maturation pathway enhances optimism on the delivery of an effective HIV vaccine.

### Limitations of the study

While our GT-immunogen successfully primed authentic V2-apex bnAb precursor B cells and elicited cross-neutralizing antibody responses, a key limitation remains: it is still unclear whether all these elicited antibody lineages can mature into fully functional bnAbs. Although the antibodies were isolated at an early time point and demonstrated specific engagement with the intended antigens, they have yet to undergo the extensive affinity maturation likely required to close the gap between their current high-affinity binding and their limited neutralization breadth. This represents a critical barrier and an area of active investigation, as the ultimate goal is to elicit broad, potent, and durable serum antibody responses capable of protecting against diverse HIV strains. In our study, the Q23-APEX-GT2 immunogen elicited a robust serum response that was able to neutralize a mutant virus lacking the N160 glycan—an essential component of the V2-apex bnAb epitope. This finding suggests that a substantial fraction of the elicited V2-apex-directed antibodies may be unable, or only partially able, to tolerate the presence of the N160 glycan. Overcoming this limitation—by promoting further antibody maturation that enables accommodation of this key glycan—may represent a key challenge for future immunogen design strategies aimed at guiding this immunodominant B cell response toward breadth. An additional limitation is the potential differences between the B cell repertoires of humans and rhesus macaques. These interspecies differences could influence both B cell activation and antibody maturation, possibly affecting the translatability of the vaccine responses observed in macaques to humans. Addressing these biological differences remains an important focus for future preclinical and clinical studies aimed at optimizing vaccine efficacy across species.

## Resource Availability

### Lead contact

Further information and requests for resources and reagents should be directed to and will be fulfilled by the lead contact, Raiees Andrabi (raiees.andrabi@pennmedicine.upenn.edu)

### Materials availability

Upon specific request and execution of a material transfer agreement (MTA) from The University of Pennsylvania to the Lead Contact. Antibody plasmids will be made available.

## STAR+METHODS

### EXPERIMENTAL MODEL AND STUDY PARTICIPANT DETAILS

#### Cell Lines

Expi293F cells (Gibco, Cat# A14527) were acquired from Gibco and cultured in Expi293 Expression Medium (Gibco, Cat# A1435101) at 37°C with 8% CO_2_ in a 125 rpm shaker. HEK293F cells (Gibco, Cat# A14527) were acquired from Gibco and cultured in Freestyle medium (Gibco, Cat# 12338-018) at 37°C with 8% CO_2_ in a 125 rpm shaker. HEK293T cells (ATCC, Cat# CRL-3216) were acquired from ATCC and maintained in Dulbecco’s Modified Eagle Medium (DMEM) (Corning, Cat# 10-017-CV) supplemented with 10% heat-inactivated fetal bovine serum (FBS) (Thermo Fisher, Cat# MT35016CV), 4 mM L-glutamine (Corning, Cat# 25-005-CI), and 1% penicillin-streptomycin (P/S) (Corning, Cat# 30-002-CI) at 37°C in a 5% CO_2_ incubator. TZM-bl 931 cells (NIH AIDS Reagents Program) were acquired from NIH AIDS Reagent Program and used for the pseudovirus neutralization assay as previously described. The cells are authenticated using STR profiling and are routinely tested and are free of any mycoplasma contamination.

#### Animal models

##### Mice

Previously described HIV-1 Envelope V2-apex PCT64^LMCA^ knock-in (KI) mouse^42^ derived B cell was used for immunization experiments to investigate the B cell priming efficiency of Q23-APEX-GT2 trimer as a protein and mRNA lipid nanoparticle. 8-12 weeks old male B6.SJL-Ptprca Pepcb/BoyJ (CD45.1 mice: purchased from the Jackson Laboratories) mice were used for B cell adoptive transfer/immunization experiments. All experiments were performed under the approval by the Institutional Animal Care and Use Committee (IACUC) of Harvard University and the Massachusetts General Hospital (MGH) and conducted in accordance with the regulations of the American Association for the Accreditation of Laboratory Animal Care (AAALAC). All animals were cared for in accordance with AAALAC standards in accredited facilities. All animal procedures were performed according to protocols approved by IACUC, specifically: Animal Study Protocols 2016N000022 and 2016N000286 (MGH).

##### Indian rhesus macaques

All 12 Indian Rhesus macaques (aged 3–5 years; evenly distributed between males and females) used in this study were housed at Bioqual, Inc. (Rockville, MD) in compliance with the guidelines set by the Association for Assessment and Accreditation of Laboratory Animal Care (AAALAC). All experimental procedures were approved by the Institutional Animal Care and Use Committees (IACUC) of the University of Pennsylvania (protocol 807492) and Bioqual (protocol 24-072). Macaques were sedated for blood collection and received care in accordance with AAALAC guidelines and best practice standards.

### METHOD DETAILS

#### Immunogen Design

To evaluate the interaction of V2-apex broadly neutralizing antibody (bnAb) precursors with key V1V2 sites, a series of site-directed mutagenesis (SDM) experiments were performed on the base germline-targeting trimer construct (Q23-APEX-GT1). Mutagenesis targeted residues within the V1V2 region (positions 130, 132, 158, 167–174) that were identified from structures of rhesus and human V2-apex bnAbs in complex with HIV trimers as potential hotspots for enhanced bnAb precursor engagement. Mutagenesis was carried out using the NEB Q5 site-directed mutagenesis kit (New England Biolabs, cat #M0494S) following the manufacturer’s protocol, and successful incorporation of mutations was verified through sequencing analysis (Eton Bioscience, San Diego, CA).

#### Stabilized Env Expression and Purification

Plasmids encoding the Env trimers were transfected into HEK293F cells using PEI-MAX 40K transfection reagent (Kyfora, cat# 24765-1). The cells were incubated, and four days posttransfection, the supernatants containing the expressed trimers were collected. Purification was carried out using affinity chromatography with either agarose-bound Galanthus nivalis lectin (GNL) (elution with 1M MMP, α-methylmannoside) (Vector Labs, cat #AL-1243-5) or TOYOPEARL AF-Tresyl-650M beads (TOSOH, cat #0014472) conjugated to the PGT145 broadly neutralizing antibody (bnAb) per bead manufacturer’s instructions. To ensure further purification, the eluates were subjected to size-exclusion chromatography (SEC) using a Superdex 200 increase 10/300 GL column (GE Healthcare, cat #GE28-9909-44) in PBS. SEC fractions corresponding to the trimer peak were pooled together and utilized for ELISA and BLI binding studies, immunizations and as baits for antigen or epitope specific single B cell flow cytometry sorting experiments. Monomeric Q23 gp120 was purified by GNL affinity followed by SEC segregation and selection of the protein fractions corresponding to the gp120 monomer peak.

#### Site-specific glycan analysis

To determine the glycosylation of the Q23_SCT27_GT2.V1 trimer, 100μg of protein was denatured for 1-hour in 50mM Tris/HCL, pH 8.0, containing 6M of urea and 5mM dithiothreitol (DTT). The sample was then incubated in the dark for 1-hour with 20mM iodoacetamide (IAA) to alkylate the protein. To remove the residual IAA, 20mM DTT was added, and the sample was incubated for an additional 1-hour period. Following buffer-exchange into 50mM Tris/HCL, pH 8.0 using Vivaspin columns (10kDa), the proteins were split into three aliquots, each containing approximately 33 μg of protein to allow for three different protease digests. The aliquots were then digested separately overnight at 37°C with either Trypsin (Promega), Chymotrypsin (Promega), or Alpha lytic protease (New England Biolabs)) in a 1:30 (w/w) ratio. Desalting and peptide enrichment was performed using an Oasis HLB desalting 96-well μElution plate (Waters) attached to a vacuum manifold. The peptides were combined and analyzed by nanoLC-ESI MS with an Easy nLC 1200 (Thermo Fisher Scientific) system coupled to an Orbitrap Fusion mass spectrometer (Thermo Fisher Scientific) using stepped higher energy collision-induced dissociation (HCD). An EasySpray PepMap RSLC C18 column (75μm x 75 cm) was used to separate the peptides. A trapping column (PepMap 100 C18 3μM 75μM x 2cm) was used in line with the LC prior to separation with the analytical column. For LC separation, buffer A consisted of 0.1% formic acid and 80% acetonitrile in 0.1% formic acid. The LC conditions were as follows: 280-minute linear gradient consisting of 5-40% B (80% acetonitrile) in 0.1% formic acid over 240 minutes. The %B was then increased to 95% over 15 minutes and held for another 15 minutes before reducing the %B to 5%. The flow rate was set to 300 nL/min. The spray voltage was set to 2.5 kV and the temperature of the heated capillary was set to 55 °C. The ion transfer tube temperature was set to 275 °C. The scan range was 350–1800 m/z. Stepped HCD collision energy was set to 15, 25 and 45% and the MS2 for each energy was combined. Precursor and fragment detection were performed using an Orbitrap at a resolution MS1= 120,000. MS2= 30,000. The AGC target for MS1 was set to standard and injection time set to auto. Glycopeptide fragmentation data were extracted from the raw MS files using Byos (Version 5.5; Protein Metrics Inc). The glycopeptide fragmentation data were evaluated manually for each glycopeptide. The peptide was scored as true-positive when both the oxonium ions corresponding to the identified glycan and the correct b and γ fragment ions were observed. The Protein Metrics 305 N-glycan library with sulphated glycans added manually, was used to search the MS data. The relative amounts of each glycan at each site in addition to the unoccupied proportion was determined by comparing the extracted chromatographic areas for different glycoforms with an identical peptide sequence. A 1% False discovery rate (FDR) was applied, and the precursor mass tolerance was set at 4 ppm, and 10 ppm for fragments. All charge states for a single glycopeptide were summed. Glycans were categorized according to the composition detected. HexNAc(2)Hex(9-3) was classified as M9-M3, HexNAc(2)Hex(10+) was defined as M9GLc and any of these structures which contained fucose were characterised as FM (fucosylated mannose). HexNAc(3)Hex(5–6)X was classified as Hybrid with HexNAc(3)Hex(5-6)Fuc(1)X classified as Fhybrid. The complex-type glycans were categorised according to the number of HexNAc subunits and the presence or absence of fucosylation. Core glycans refer to truncated structures smaller than M3. M9glc- M4 were classified as oligomannose-type glycans. The oligomannose- and hybrid-type glycans were combined into a high mannose glycan category.

#### Enzyme-Linked Immunosorbent Assay (ELISA)

ELISA assays were conducted using biotinylated proteins on streptavidin-coated plates, following previously established protocols. In brief, 96-well half-area clear plates (Corning, Thermo Fisher Scientific) were coated overnight at 4°C with 2 μg/mL streptavidin (Jackson ImmunoResearch, cat #016-000-113). The plates were then washed three times with 1X PBS/0.05% Tween (Sigma-Aldrich, cat #1003620819) and blocked with 100ul 3% BSA (Sigma-Aldrich, cat #A9418-500G) in PBS for 1 hour at room temperature (RT). BSA was dumped from the wells and plates were patted dry. Biotinylated proteins were added at a concentration of 2 μg/mL in 1% BSA/1X PBS/0.05% Tween and incubated for 1.5 hours at RT. Following incubation, plates were washed three times before adding diluted monoclonal antibodies (mAbs) or serum samples, which were incubated for an additional 1.5 hours. After washing, alkaline phosphatase-conjugated secondary antibodies (Jackson ImmunoResearch Laboratories, cat #109-055-170) were applied 50ul/well at 1:1000 dilution and incubated for 1 hour. For the phosphatase substrate (Thermo Fisher Scientific, cat #S0942-200TAB) 1 tablet was dissolved per 5 ml of Alkaline staining buffer 2.03g MgCl_2_-6H_2_O (Fisher Bioreagents, cat #BP214-500), 8.4g Na_2_CO_3_ (Sigma-Aldrich, cat #S7795-500G) and 1 g NaN_3_ was added in MilliQ water, pH was adjusted to 9.8, the final volume was adjusted to 1L (Sigma-Aldrich, cat #S2002-100G), and filtered through 0.22μm filter. Absorbance was measured at 405 nm using a Synergy HTX multi-mode reader after 20 minutes of substrate development.

#### Cell Surface Binding Assay

HEK293T cells were seeded in 6-well plates and transfected with plasmids encoding the antigen of interest using Lipofectamine 2000 (Thermo Fisher Scientific, cat #11668500), following the manufacturer’s protocol. After 48 hours of incubation at 37°C with 5% CO_2_, cells were harvested using FACS buffer (PBS + 2% FBS+5mM EDTA (Invitrogen, cat #15575-038), followed by washing twice with FACS buffer and resuspended at a density of 1 × 10^6^ cells/mL. Cells were incubated with primary antibodies of interest at a final concentration of 10 μg/mL for 1 hour on ice. After incubation, cells were washed three times with FACS buffer and subsequently stained with Mouse Anti-Human IgG FC-PE (SouthernBiotech, cat #9040-09) for 1 hour on ice in the dark. Following a final set of washes, cells were resuspended in FACS buffer and acquired on a Bio-Rad ZE5 flow cytometer. Data were analyzed using FlowJo software.

#### Biolayer Interferometry

For high throughput antigenicity screening of Q23-APEX immunogen designs and of isolated mAbs from immunized animals, BLI was performed with 10 ug/ml IgG antibody in running buffer (1X PBS, 0.02% Tween20, pH 7.4). IgGs were immobilized on ProA sensors (Sartorius, cat #18-5012) to a signal of at least 1.0 nm using an Octet Red96 instrument (ForteBio). The immobilized IgGs were then dipped in the running buffer followed by 500 nM of trimers. Following a 120 s association period (with IgGs dissolved in running buffer), the tips were dipped into the running buffer (without IgGs) and dissociation was measured for 240 s. For assessing the polyclonal immune serum IgG responses in vaccinated animals, serum was used with a 1:10 dilution (in running buffer). Biotinylated trimers were first captured on SA biosensors (Sartorius, cat #18-5020) to a signal of at least 1.0 nm using an Octet Red96 instrument (ForteBio). The immobilized trimers were then dipped in the running buffer followed by polyclonal serum IgG. Following a 120 s association period, the tips were dipped into the running buffer and dissociation was measured for 240 s. For determining BLI kinetics with Fab versions of mAbs, monoclonal IgG Fab heavy chain plasmids were engineered by inserting a His-Avi tag, followed by a stop codon, upstream of the disulfide bond in the Fc region. Paired heavy and light chain plasmids were co-transfected along with BirA for biotinylation into Expi293 cells (Thermo Fisher Scientific, cat #A14527) at a 2:2:1 ratio (HC:LC:BirA) using FectoPRO transfection reagent (Polyplus, cat #116-001). After 24 hours, the cells were supplemented with 0.3 M valproic acid (Sigma, cat #P4543-100G) and 40% glucose (Gibco, cat #A2494001). MAb IgG Fabs were harvested from the culture supernatant five days post-transfection by affinity chromatography using Ni Sepharose 6 Fast Flow (Cytiva, cat #17531802), according to the manufacturer’s instructions. The eluted antibodies were buffer exchanged into PBS and concentrated using a 10 kDa ultracentrifugal filter (Millipore, cat #UFC905024). Concentrated Fabs were subjected to size exclusion chromatography on a Superdex 200 Increase 10/300 GL column (Sigma-Aldrich, cat #GE28-9909-44). Specific fractions were pooled, further concentrated. BLI was performed as before with 10ug/ml Fab. Fabs were immobilized on SA sensors (Sartorius, cat #18-5020) to a signal of at least 1.0 nm using an Octet Red96 instrument (ForteBio). The immobilized Fabs were then dipped in the running buffer followed by 2-fold dilution of trimers starting at 500nM. Following a 120 s association period, the tips were dipped into the running buffer and dissociation was measured for 240 s.

#### Neutralization Assay

Sera (1:10 starting dilution) or monoclonal antibodies (300 μg/ml starting concentration) were three-fold serially diluted in 25 μl of complete DMEM and incubated with HIV-1 Env-pseudotype virus (25 μl) for 60 minutes at 37°C in duplicate 96-well Culture Plates. TZM-bl cells (20,000 cells per well) with 20 μg/ml DEAE-Dextran were then added (50 μl) and incubated overnight. Control wells included cells only (background) and virus only (maximal entry). Serial dilutions were performed with tip changes to prevent carryover. After 72 hours, luciferase activity was measured using the Bright-Glo Luciferase Reporter Assay (Promega, cat #E2650) and a Synergy HTX multi-mode luminometer. Percent neutralization was calculated as: ((RLU_Virus_ – RLU_test_) / RLU_Virus_) x 100 Background RLU from uninfected control wells was subtracted before final calculations. Neutralizing serum titers (ID_50_) and antibody titers (IC_50_) were determined via a four-parameter nonlinear dose-response inhibition curve.

#### PCT64^LMCA^ knock-in mouse adoptive transfer immunization study

Previously described PCT64^LMCA^ knock-in (KI) mice ^42^ donor derived CD45.2 B cells were isolated from spleens using Pan B Cell isolation kit (Milteny Biotec) following manufacturer’s protocol. Cells were counted using Luna FL cell counter. B cells were resuspended in PBS and 1 x 10^5^ B cells were injected intravenously in tail vein of recipient 8-12 weeks old B6.SJL-Ptprca Pepcb/BoyJ (CD45.1 mice). 1 day post adoptive transfer groups of recipient animals (n = 5 animals per group) were immunized either with SMNP adjuvant only (control 5 μg) or 20 μg of Q23-APEX-GT2 trimer protein adjuvanted with 5 μg of SMNP subcutaneously (SC) at the base of the tail or 1μg of Q23-APEX-GT2 mRNA lipid nanoparticle (LNPs) (provided by Moderna) intramuscularly (IM) in each hindleg. For animals receiving SC immunization draining inguinal lymph nodes and for animals receiving IM immunization iliac and popliteal lymph nodes were harvested at week 2, 4 post immunization. Immune sera at pre-immunization, weeks 2 and 4 were also collected.

#### Analysis of PCT64^LMCA^ knock-in mouse B cell responses

Single cell suspension was prepared by gently crushing lymph nodes and passing them through a 70 μM strainer. Incubation with PBS containing Live/Dead Blue (Thermo Scientific) diluted 500-fold and FcR Blocking reagent (Purified Rat anti-mouse CD16/CD32, BD Biosciences) diluted 200-fold was done for 20 mins at 4°C. After washing, BCR antigen staining was done using biotinylated Q23-APEX-GT2 trimer conjugated to either streptavidin-BV510 (BioLegend) or strepatvidin-Alexa647 (BioLegend) for 30 mins at 4°C. Excess antigen was washed off and, cells surface staining was done with an antibody cocktail containing CD4, CD8, F4/80, GR-1, NK1.1 (APC-eFluor 780, eBioscience, clone RM4-5, 53-6.7, BM8, RB6-8C5, PK136 respectively), B220 (BUV395, BD Bioscience, clone RA3-6B2), CD38 (BUV563, BD Biosciences, clone 90), CD95 (PE-Cy7, BioLegend, clone L138D7), CD45.1 (BV605, BioLegend, clone A20), CD45.2 (PE, BD Biosciences, clone 104), CD138 (BV650, BD Biosciences, clone 281-2), IgD (Alexa 594, Biolegend, 11-26c.2a clone) and IgM (BV750, II/41 clone) for 30 mins at 4°C. Flow cytometry data was acquired using BD FACS Symphony A5 cell analyzer. For cell sorting, Live/Dead stain was replaced with SYTOX Green (Thermo Fisher Scientific). The antibodies used for sorting were CD4, CD8, F4/80, GR-1 & NK1.1 (APC-eFluor 780, eBioscience, clone RM4-5, 53-6.7, BM8, RB6-8C5, PK136 respectively), B220 (Alex Fluor594 BioLegend, clone RA3-6B2), CD38 (BB700, BD Biosciences, clone 90), CD95 (PE-Cy7, BioLegend, clone L138D7), CD45.1 (APC R700, BD Biosciences, clone A20), CD45.2 (PE, BD Biosciences, clone 104), IgD (BV605, BioLegend, clone 11-26c.2a), CD138 (BV650, BD Biosciences, clone 281-2). Cells from each individual mouse were barcoded with TotalSeq^™^-C anti-mouse Hashtag Antibodies. A total of 10 hashtags were used. Cells were washed 3 times to remove any excess antibodies.

#### Cell sorting and paired BCR sequencing of PCT64^LMCA^ B cell responses

Cells were sorted using BD FACS symphony S6 using 85μM nozzle. Samples were sorted onto PCR tubes with PBS buffer containing 10% FBS. Encapsulation of sorted cells and NGS library preparation was performed following the 10x Genomics Chromium Next GEM Single Cell 5’ Reagent Kits v2 protocol (10x Genomics). TapeStation Systems D5000 high sensitivity Screen Tape assay (Agilent, Santa 5 Clara, CA) was used to measure library size. After quantifying the libraries through Qubit dsDNA High Sensitivity (Invitrogen), they were pooled and were run on NextSeq 550 System (Illumina, San Diego, CA). Analysis was performed using Cell Ranger v.6 software pipeline (10x Genomics) with a customized reference database. Sequencing data was analyzed using Geneious Prime software (Geneious) and IMGT/V-Quest.

#### Immunization in rhesus macaques, blood and lymph node processing

2 groups of 6 rhesus macaques each, evenly distributed by gender between the ages 3-5 years, were immunized with germline-targeting trimer, Q23-APEX-GT2 trimer protein + SMNP adjuvant^68^ (Arm 1) and membrane-anchored mRNA LNPs (Arm 2). Rhesus macaques were primed at week 0 with 100 μg Q23-APEX-GT2 protein + SMNP adjuvant (Arm 1) or 100 μg Q23-APEX-GT2 mRNA LNPs (Arm 2) administered subcutaneously and distributed into four injection sites (split equally between bilat inner mid-upper arm and bilat inner mid-thighs, 25 μg each). The Arm 1 Q23-APEX-GT2 protein priming immunization was given as escalating dose (DE) over 2 weeks ^69,70^ and a single bolus dose for Q23-APEX-GT2 mRNA LNPs in Arm 2. For escalating dose priming, animals received seven injections of the Q23-APEX-GT2 protein along with the SMNP adjuvant across four sites over 12 days (on days 0, 2, 4, 6, 8, 10, and 12). The total Q23-APEX-GT2 trimer immunogen doses at each injection were: 0.2, 0.43, 1.16, 3.15, 8.56, 23.3, and 63.2 μg, evenly distributed across the four immunization sites. Protein prime was co-administered with 375 μg of SMNP adjuvant, which was delivered in a proportional dose-escalation manner alongside the seven protein immunogen injections. Both groups were bolus boosted with 100 μg of Q23-APEX-GT2 protein + SMNP adjuvant (Arm 1) or Q23-APEX-GT2 mRNA LNPs (Arm 2).

Peripheral blood was collected in sterile vacutainers containing acid citrate dextrose formula A (ACD-A) as an anticoagulant (DB Vacutainer cat #364606). A total of 40 mL of ACD-A blood was centrifuged at 1000g for 10 minutes at 20°C in sterile 50 mL conical tubes. Plasma was collected without disturbing the buffy coat or red blood cell pellet, then subjected to a second centrifugation at 1500g for 15 minutes at 20°C to remove all cellular material. The cell-depleted plasma was aliquoted into 1 mL cryovials (Sarstedt cat # 72.694.396) and stored at −80°C. The cell fraction was resuspended in an equal volume of Hanks’ Balanced Salt Solution (HBSS) without calcium or magnesium (HBSS−/−) (Gibco cat # 14175-079) containing 2 mM EDTA (Invitrogen cat #15575–020) and divided into four 50 mL conical tubes. Additional HBSS−/− with EDTA was added to each tube to bring the total volume to 35 mL. The cell suspension was under layered with 14 mL of 96% Ficoll-Paque Plus (Cytiva cat # 17144003) and centrifuged at 725g for 20 minutes at 20°C with slow acceleration and braking. Mononuclear cells at the Ficoll interface were collected, transferred to a fresh 50 mL conical tube containing HBSS−/− with EDTA, and washed by centrifugation at 200g for 15 minutes at 20°C. Following removal of the supernatant, the cell pellet was resuspended in 40 mL of HBSS containing calcium and magnesium (HBSS+/+) (Gibco cat # 24020-117) supplemented with 1% fetal bovine serum (FBS) (Cytiva cat # SH300.71.03). The suspension was centrifuged at 200g for 15 minutes at 20°C, after which the supernatant was discarded. This centrifugation step effectively pelleted white blood cells (WBCs) while leaving most platelets in suspension. The mononuclear cell pellet was gently resuspended in the residual media, followed by the addition of HBSS+/+ with 1% FBS to a final volume of 10 mL. Cells were counted, and viability was assessed using ViaStain AOPI solution (Revvity cat #CS2-0106-25ml) and a Cellometer Auto 2000 instrument (Revvity, Waltham MA). The cells were then centrifuged at 300g for 10 minutes at 20°C, the supernatant was discarded, and the pellet was resuspended in CryoStor CS5 cryopreservation medium (Stemcell technologies cat # 07930) at a final concentration of 5–10 × 10^6^ cells/mL. The suspension was aliquoted into 1 mL cryovials (Thermoscientific cat # 374503), stored in a Corning CoolCell LX (Corning cat #432002) or FTS30 (Corning cat #432006) freezing container at −80°C overnight, and subsequently transferred to vapor-phase liquid nitrogen for long-term storage. Mononuclear cells from lymph nodes (LNs) were processed in a similar protocol to blood mononuclear cells. Axillary and/or inguinal LNs were excised, placed immediately into RPMI1640 medium (Corning cat # MT15040CV) on wet ice and processed within 6 hours. LNs were diced with a sterile scalpel and passed through a sterile cell strainer (Falcon cat # 352360). Cells were collected from the pass-through and subjected to Ficoll density gradient purification as described above, with RPMI1640 supplemented with 10% FBS used instead of HBSS.

#### Flow cytometry antigen-specific B cell sorting of macaque lymph node samples

Avi-tagged, biotinylated Q23-APEX-GT2 trimer and its double knockout variant (169E-171E) were conjugated to streptavidin-labeled fluorophores at room temperature for 30 minutes. Cryopreserved LN samples were thawed and resuspended in RPMI medium (Thermo Fisher, cat # MT15040CV) supplemented with 50% FBS. Cells were washed with FACS buffer (PBS + 2% FBS + 2mM EDTA) (Invitrogen, cat #15575-038) and stained with fluorescently labeled anti-CD3 (BD Pharmingen 557757), CD4 (Biolegend 317418), CD8 (Biolegend 557760), CD14(BD Pharmingen 561384), CD19 (Biolegend 302230), CD20 (Biolegend 302326), IgG(BD Horizon 564230, and IgM(Biolegend 314508) antibodies for 30 minutes on ice in the dark. The conjugated antigens were then added and incubated for another 30 minutes. A 1:250 dilution of FVS510 LIVE/DEAD stain (Thermo Fisher Scientific, cat #L34966) was applied, followed by a 15-minute incubation. Before sorting, cells were washed with FACS buffer and passed through a cell strainer into a 5 mL round-bottom tube (Corning 352058). Sorting was conducted on a BD FACSMelody, and cells were collected into 96-well PCR plates for single-cell sequencing. RNA extraction, cDNA synthesis, and VDJ gene amplification were performed as previously described. Briefly, RT-PCR was performed using Superscript IV reaction and IgH, IgK, and IgL primers. Paired HC and LC sequences were amplified using nested PCR reactions and analyzed by 2% 96 E-gels with SYBR Safe (Thermo Fisher Scientific cat #G720802). Wells with successful DNA PCR amplification were Sanger sequenced (Azenta).

#### Antibody Cloning

IgG heavy and light chain sequences for sorted B cell derived antibodies with features characteristic of rhesus V2-apex bnAbs from all immunized animals were cloned in IgG1, IgK, IgL AbVec Vector upstream of respective constant region using AgeI/NheI, AgeI/BsiWI and AgeI/SaII restriction sites respectively.

#### Monoclonal Antibody Expression and Purification

Paired heavy and light chain plasmids for Rhesus and human V2-apex UCAs or monoclonal antibodies cloned from immunized animals were co-transfected in Expi293 cells (Thermo Fisher Scientific, cat #A14527) in a 1:1 ratio using FectoPRO (Polyplus, cat #116-001) transfection reagent and were fed with 0.3M valproic acid (Sigma, cat #P4543-100G) and 40% Glucose (Gibco, #A2494001) 24 hours after the transfection. Monoclonal IgGs were purified from the culture supernatant five days post-transfection with 1:1 ratio Protein-A (Cytiva, cat #17127903) and Protein-G Sepharose beads (Cytiva, cat #17061805) per manufacturer’s instructions. After elution with IgG elution buffer (Thermo Fisher Scientific, cat #PI21009), antibodies were buffer exchanged into PBS using 50kDa Ultra centrifugal filter unit. (Millipore, cat #UFC905024)

#### Bulk Repertoire Sequencing and Immunogenetic Analysis

Cryopreserved lymph node samples were rapidly thawed in a 37°C water bath for 1–2 minutes until a small ice pellet remained. The thawed cells were immediately transferred to pre-warmed RPMI medium (Thermo Fisher, cat #MT15040CV)) supplemented with 50% FBS in a dropwise manner while gently swirling to minimize osmotic shock. Cells were centrifuged at 400 × g for 5 minutes at room temperature, and the supernatant was carefully aspirated to remove residual cryoprotectants. The cell pellet was resuspended in fresh RPMI medium, and viability was assessed using trypan blue exclusion (Sigma T8154). For RNA extraction, cells were lysed in RLT Plus buffer (Qiagen, cat #1053393) supplemented with β-mercaptoethanol, and total RNA was extracted using the RNeasy Plus Mini Kit (Qiagen, cat #74134) according to the manufacturer’s instructions, including on-column DNase I treatment (Qiagen cat #79254) to eliminate genomic DNA contamination. RNA quality and concentration were assessed using a NanoDrop spectrophotometer (Thermo Fisher Scientific) and the Agilent 2100 Bioanalyzer RNA Integrity Number (RIN) score. Only samples with RIN ≥ 7 were used for downstream applications. Reverse transcription was performed using gene-specific primers targeting IgM and IgG constant regions. cDNA synthesis was carried out with SuperScript IV reverse transcriptase (Thermo Fisher Scientific, cat #1750150) following the manufacturer’s protocol, with an initial primer annealing step at 65°C for 5 minutes, followed by reverse transcription at 55°C for 60 minutes and enzyme inactivation at 70°C for 15 minutes. The resulting cDNA was purified using ExoSAP-IT (Thermo Fisher Scientific, cat #78205) to remove excess primers and dNTPs. Immunoglobulin heavy-chain variable region (VDJ) amplification was performed in two sequential PCR steps using HotStarTaq Plus DNA Polymerase (Qiagen, cat #203603). The first PCR utilized a set of framework region primers spanning VDJ segments, followed by a nested PCR with primers incorporating Illumina-compatible overhang sequences. PCR products were enzymatically cleaned using ExoSAP-IT, and Illumina sequencing adapters with unique dual indexes were introduced via a second round of PCR. Final DNA libraries were purified using SPRIselect beads (Beckman Coulter Genomics, cat #B23318) with a 0.8x bead-to-sample ratio to remove adapter dimers and short fragments. Library concentration was quantified using a Qubit fluorometer (Thermo Fisher Scientific), and fragment size distribution was assessed using a Bioanalyzer (Agilent 2100) High Sensitivity DNA chip. Sequencing libraries were pooled in equimolar concentrations and loaded onto an Illumina NovaSeq 6000/NextSeq 1000/2000 system using a 2 × 300 bp paired end read configuration.

#### B-cell Annotation / VDJ Repertoire Analysis

Filtered and quality-controlled IgM and IgL sequences were analyzed using IgDiscover v0.15.1 to curate a personalized immunoglobulin repertoire library for each rhesus macaque (Table S3). Merged reads served as input, with the KIMDB 1.1 database (http://kimdb.gkhlab.se/) as the reference for heavy chains and the Ramesh et al. allele database for light chains. To enhance accuracy, the IgDiscover J output for both heavy and light chains was further processed using the “discoverjd” feature, applying a J coverage threshold of 100 and an allele ratio cutoff of 0.33 to exclude low-confidence novel alleles. This approach enabled the identification of both known and novel V and J gene alleles for each rhesus macaque, confirmed the presence of known D gene alleles, and facilitated the precise assignment of V, D, and J alleles for each broadly neutralizing antibody lineage.

#### Antibody Clonotypes

Antibody clonotypes were defined as sequences sharing the same V and J germline segments and identical CDRH3 amino acid sequences to minimize sequencing and amplification errors. While collapsing V and J regions to germline assignments prevents double-counting errors in these regions, it does not address errors in CDRH3. To assess their impact on clonotype diversity, we grouped sequences into clonotypes with no mismatches in the CDRH3 sequence.

#### Ployclonal Fab preparation

To generate polyclonal serum Fabs for EMPEM studies, poly-IgG was first isolated from immune sera by rotating 1ml of serum with 0.25ml of Protein A Affinity Chromatography Resin (Cytiva, cat #17127903) and 0.25 ml Protein G Sepharose (Cytiva, cat #17061805), and 9ml PBS. The next day, beads were transferred to an Econo-Pac column (Bio-Rad Laboratories), washed with 2 column volumes of PBS, and then eluted with 10ml IgG elution buffer (Thermo Fisher Scientific, cat #PI21009). After elution, using 50kDa Ultra Centrifugal Filter Units (Merck Millipore cat #UFC9030) to buffer exchange poly-IgG Abs into PBS and then concentrated. Polyclonal antibodies and mAbs were then digested using Pierce^™^ Fab Preparation Kit (Thermo Fisher cat #44985) following manufacturer instructions. After digestion, 10 kDa Ultra Centrifugal Filter Units (Millipore, cat# UFC9010) were used to buffer exchange proteins into PBS and concentrate to 0.5ml. Concentrated Fabs underwent size exclusion chromatography using Superdex 200 Increase 10/300 GL column (Sigma-Aldrich, cat #GE28-9909-44). Specific fractions from the chromatography run were mixed and concentrated before being used.

#### EMPEM data collection and processing

15 μg of Q23-APEX-GT2 trimer was incubated with 500 μg of polyclonal Fab overnight at room temperature before purifying through size exclusion chromatography with a Superdex 200 Increase 10/300 column (GE Healthcare). The complex was diluted to 0.02 mg/ml in 1x Tris-buffered saline pH 7.4, and 3 μl were applied to a 400 mesh Cu grid, blotted with filter paper and stained with 2% uranyl formate or NanoW (Nanoprobes). Samples were imaged on either a 200 kV Thermo Fisher Scientific Glacios with a Falcon IV direct electron detector (1.89 Å/pixel; 73,000x magnification) or a 200 kV Thermo Scientific Talos F200C with a Ceta 16M camera (2 Å/pixel; 73,000x magnification) using EPU software. Particle picking, 2D classification and 3D reconstructions were done using Relion 4.0^95^. Following three rounds of 2D classification, particles from classes corresponding to immune complexes were subjected to 3D refinement with C3 symmetry and a 40 Å low-pass filtered map of HIV Env ectodomain as the initial model. The initial model is based on PDB coordinates 6V0R, converted in a map using the molmap feature in UCSF ChimeraX^96^. Following 3D refinement, C3 symmetry expansion was applied to the particles and 7 separate focused 3D classification “skip align” jobs were run (K=10), each with a 40 Å diameter spherical mask over key HIV Env epitopes. The names of the epitopes and reference structures used for orienting the masks are: 1) gp41-base (PDB 6X9R), 2) gp41-GH (PDB 7L8U), 3) gp41-FP (PDB 7L8T), 4) gp120-GH (PDB 7L8B), 5) C3V5 (PDB 7L86), 6) CD4bs and gp120 interface (PDB 7L8X), and 7) V1V2V3 (PDB 7L8E). For each epitope 3D classification, classes with visible Fab density were selected and subjected to 3D refinement, 2D classification, and a second round of 3D classification. If the 3D refinement resulted in partial Fab density relative to the Env trimer, classes were selected from the subsequent round of 3D classification, and this was repeated until the reconstruction improved, or no change was noted. Final reconstructions were visually inspected and assigned the correct epitope label. The number of final particles belonging to each epitope was divided by the total number of particles in the initial (C3) 3D refinement. This value, which can range (per epitope) from 0 to 3 due to C3 symmetry expanded particles used in the 3D classification steps, is described as the *EMPEM magnitude*. The sum of each individual *EMPEM magnitude* is the *Total EMPEM magnitude*. 3D maps were segmented and figures generated using UCSF Chimera^97^. Fab densities that could not be resolved in 3D are presented as false-colored 2D classes. Representative EM maps have been deposited to the Electron Microscopy Data Bank (EMDB).

#### Monoclonal Fab preparation for Cryo-EM structural studies

Monoclonal IgG Fab heavy chain plasmid was designed by inserting a His-Avi tag followed by a stop codon before the disulfide bond in the Fc region. Paired truncated heavy and light chain plasmids were co-transfected in Expi293 cells (Thermo Fisher Scientific, cat #A14527) in a 1:1 ratio using FectoPRO (Polyplus, cat #116-001) transfection reagent and were fed with 0.3M valproic acid (Sigma, cat #P4543-100G) and 40% Glucose (Gibco, #A2494001) 24 hours after the transfection. Fabs were purified from the culture supernatant five days post-transfection with Ni Sepharose 6 Fast Flow (cytiva, cat#17531802) per manufacturer’s instructions. After elution, Fab proteins were buffer exchanged into PBS and concentrated using 10kDa Ultra centrifugal filter unit. (Millipore, cat #UFC905024) Concentrated Fabs underwent size exclusion chromatography using Superdex 200 Increase 10/300 GL column (Sigma-Aldrich, cat #GE28-9909-44). Specific fractions from the chromatography run were pooled and concentrated before being used.

#### Cryo-EM data collection and processing

The high-resolution unbound (apo) and Fab-bound structures of Q23-APEX-GT2 envelope trimer were determined using single-particle cryo-EM as previously described^98^. Samples were prepared by diluting purified Q23-APEX-GT2 to 2.5 mg/mL with PBS or 3-fold molar excess of Fab:trimer and incubated on ice for 30 minutes, and then supplemented with a final concentration of 0.005% (w/v) n-Dodecyl β-D-maltoside (DDM) to prevent preferred orientation. Copper C-flat Holey carbon-coated grids (CF-1.2/1.3 300 mesh; EMS) were first glow discharged and then applied with 3 uL of sample for 30 seconds in a Vitrobot Mark IV at room temperature with 100% humidity. Sample grids were then vitrified using liquid ethane. Cryo-EM data were collected on a FEI Titan Krios 300 kV cryo-transmission electron microscope equipped with a Gatan K3 detector operating in counting mode using Leginon^99^. Defocus values were set to cycle between −0.80 and −2.0 μm, and a total dose of 58 e^−^/Å^2^ was fractionated over 50 raw frames. All processing was done in cryoSPARC v3.4^100^, including motion correction, CTF estimation, non-templated blob particle picking, 2D classification, *ab initio* modeling, and iterative 3D refinements. All homogenous and non-uniform 3D refinements of Fab-bound complexes were performed using C1 symmetry, while all homogenous 3D refinements and non-uniform 3D refinements for apo Q23-APEX-GT2 were performed with C1 and C3 symmetry, respectively. Data acquisition and processing details for all structures are provided in Dataset S1.

#### Atomic model building and refinement

Each atomic model was solved by iterative manual rebuilding in Coot^101^ and real-space refinement in Phenix ^102^, with the overall structure quality for all models periodically assessed using MolProbity ^103^ and EMRinger ^104^ during refinement until satisfactory validation of each model was achieved. The initial model used for apo Q23-APEX-GT2 was another Q23.17-based prefusion-stabilized envelope trimer (PDB-7LLK) ^105^, which was fit into our 2.9 Å cryo-EM reconstruction density using UCSF ChimeraX ^106^. For atomic building of Fab complexes, the initial model of each Fab variable region (Fv) was first obtained using both the AlphaFold3 server ^107^ and the AbodyBuilder2 application of the SAbPred Antibody Prediction Toolbox ^108^; each Fv model for a specific Fab was fit into the respective cryo-EM 3D reconstruction using ChimeraX and the initial model bearing the HCDR3 with the best fit HCDR3 was selected for model building along with our apo Q23-APEX-GT2 structure. Protein interface calculations were performed using PDBePISA ^109^. Final model statistics and validations are provided in Dataset S1.

### QUANTIFICATION AND STATISTICAL ANALYSIS

Statistical analyses were performed in GraphPad Prism and R. Statistical significance was assessed using a two-tailed Student’s t-test. P < 0.05 was considered statistically significant. *P < 0.05; **P < 0.01; ***P < 0.001; ****P < 0.0001; ns, no significant difference.

## Supplementary Material

1 Dataset S1 - CryoEM data collection and processing, related to Figure 1 and Figure 6.

2 Table S1 - Q23-APEX-GT1 and Q23-APEX-GT2 neutralization, related to Figure 1

3 Table S2 - Rhesus Macaque specific oligo pool used for BCR amplification, related to figure 4

4 Table S3 - Epitope-specific antibody sequences from immunized Rhesus Macaques, related to Figure 4

5 Table S4 - Binding and neutralization profile of multi-tier HIV panel from vaccine induced mAbs, related to Figure 5

6 Table S5 - Potential V2-Apex precursors frequency in naive human repertoire, related to supplementary Figure 7

## Figures and Tables

**Table:** Key Resources Table

REAGENT or RESOURCE	SOURCE	IDENTIFIER
**Chemicals, Peptides, and Recombinant Proteins**		
40 K polyethylenimine (PEI) MAX	Kyfora	Cat# 24765-1
FectoPRO	Polyplus	Cat# 116-001
Lipofectamine 2000	Fisher Scientific	Cat# 11668500
Valproic acid sodium salt	Sigma	Cat# P4543-100G
D-(+)-Glucose Solution	Gibco	Cat# A2494001
L-glutamine	Corning	Cat# 25-005-CI
Ig Elution Buffer	Thermo Fisher Scientific	Cat# PI21009
Penicillin-streptomycin	Corning	Cat# 30-002-CI
DEAE-dextran	Sigma-Aldrich	Cat# 93556-1G
Phosphatase substrate	Sigma-Aldrich	Cat# S0942-200TAB
Expi293 Expression Medium	Thermo Fisher Scientific	Cat# A14351-01
Freestyle Media	Thermo Fisher Scientific	Cat# 12338-018
OptiMEM	Thermo Fisher Scientific	Cat# 31985070
DMEM	Corning	Cat# 10-017-CV
EDTA	Invitrogen	Cat# 15575-038
RPMI	Corning	Cat# MT15040CV
FBS	Thermo Fisher Scientific	Cat# MT35016CV
FBS	Cytiva	Cat# SH300.71.03
Trypan Blue	Sigma	Cat# T8154
Dnase 1	Qiagen	Cat# 79254
HBSS−/−	Gibco	Cat# 14175-079
HBSS+/+	Gibco	Cat# 24020-11
EDTA	Invitrogen	Cat# 15575-020
Cryostor	Stemcell	Cat# 07930
Ficoll Plaque Plus	Cytiva	Cat# 17144003
Bovine Serum Albumin	Sigma-Aldrich	Cat# A9418-500G
Tween20	Sigma-Aldrich	Cat# 1003620819
SuperScript^™^ IV Reverse Transcriptase	ThermoFisher Scientific	Cat# 1750150
ExoSAP-IT^™^ PCR Product Cleanup Reagent	ThermoFisher Scientific	Cat# 78205
HotStarTaq Plus DNA Polymerase	ThermoFisher Scientific	Cat# 203603
Q5 Hot Start High-Fidelity 2X Master Mix	New England Biolabs	Cat# M0494S
AgeI-HF	New England Biolabs	Cat# R3552S
NheI-HF	New England Biolabs	Cat# R3131S
BsiWI-HF	New England Biolabs	Cat# R3553S
SalI-HF	New England Biolabs	Cat# R3138S
SPRIselect	Beckman Coulter Genomics	Cat# B23318
Magnesium Chloride Hexahydrate	Fisher bioreagents	Cat# BP214-500
Sodium carbonate	Sigma-Aldrich	Cat# S7795-500G
Sodium azide	Sigma-Aldrich	Cat# S2002-100G
Acetonitrile, 80%, 20% Water with 0.1% Formic Acid, Optima LC/MS	Fisher Scientific	Cat# 15431423
Water with 0.1% Formic Acid (v/v), Optima^™^ LC/MS Grade	Fisher Scientific	Cat# LS118-212
Acetonitrile	Fisher Scientific	Cat# 10489553
Trifluoroacetic acid	Fisher Scientific	Cat# 10155347
Dithiothreitol	Sigma-Aldrich	Cat# 43819
Iodoacetamide	Sigma-Aldrich	Cat# I1149
Mass spectrometry grade trypsin	Promega	Cat# V5280
Sequencing grade chymotrypsin	Promega	Cat# V1061
α-Lytic protease	New England Biolabs	Cat# P8113S
Urea	Sigma-Aldrich	Cat# U5378-1KG
Anti-human IgM (PE)	Biolegend	Clone ID: MHM-88, Cat# 314508
Anti-human CD14 (APC-Cy7)	BD Pharmingen	Clone ID: M5E2, Cat# 561384
Anti-human IgG (BV786)	BD Horizon	Clone ID: G18-145, Cat# 564230
Anti-monkey IgG (H/L)	Bio Rad	Cat# AAI42
Mouse Anti-Human IgG FC-PE	SouthernBiotech	Cat# 9040-09
Alkaline Phosphatase-conjugated Goat Anti-Human IgG	Jackson ImmunoResearch	Cat# 109-055-170
NanoW	Nanoprobes	Cat# 2018
Uranyl formate	Electron Microscopy Sciences	Cat# 22450
Tris Proteomics Grade	VWR Life Science	Cat# M151
**Bacterial and Virus Strains**		
pSG3Δenv plasmid	NIH AIDS Reagent Program	Cat# 11051
MLV	NIH AIDS Reagent Program	N/A
Tier 2 Global Neutralization Panel	NIH AIDS Reagent Program	N/A
Q23-GT2 Virus mutants	This study	N/A
**Deposited Data**		
Nucleotide sequences of monoclonal antibodies	This study	Genbank Accession numbers OR517381- OR517472
Nucleotide sequences of antibodies from PCT64 LMCA mice	This study	Genbank Accession numbers PV237254-PV239402
CH35 V1V2V3 and gp41-base polyclonal Fabs in complex with Q23-APEX-GT2 trimer (negative stain EMPEM	This study	EMDB: 49511
CH35 gp41-FP polyclonal Fab in complex with Q23-APEX-GT2 trimer (negative stain EMPEM)	This study	EMDB: 49512
CH70 gp41-GH polyclonal Fab in complex with Q23-APEX-GT2 trimer (negative stain EMPEM)	This study	EMDB: 49513
Cryo-EM reconstruction and structure of apo Q23-APEX-GT2 envelope trimer	This study	PDB: 9NVV EMDB: 49865
Cryo-EM reconstruction and structure of CH35-Apex1.08 Fab in complex with Q23-APEX-GT2	This study	PDB: 9NVW EMDB: 49866
Cryo-EM reconstruction and structure of CI91-Apex1.0 Fab in complex with Q23-APEX-GT2	This study	PDB: 9NVX EMDB: 49867
Cryo-EM reconstruction and structure of CH70-Apex2.01 Fab in complex with Q23-APEX-GT2	This study	PDB: 9NVY EMDB: 49868
Cryo-EM reconstruction and structure of CH70-Apex1.01 Fab in complex with Q23-APEX-GT2	This study	PDB: 9NVZ EMDB: 49869
Cryo-EM reconstruction and structure of CH42-Apex1.01 Fab in complex with Q23-APEX-GT2	This study	PDB: 9NW0 EMDB: 49870
Cryo-EM reconstruction and structure of CH42-Apex2.01 Fab in complex with Q23-APEX-GT2	This study	PDB: 9NW1 EMDB: 49871
NGS Sequencing Runs	This study	NCBI SRA: SRX27822716-SRX27822762
**Experimental Models: Cell Lines**		
Expi293F cells	Fisher	Cat# A14527
HEK293F cells	Fisher	Cat# R79007
HEK293T cells	ATCC	Cat# CRL-3216
TZM-b1 cells	NIH AIDS Reagents Program	N/A
**Software and Algorithms**		
IMGT/V-Quest	International ImMunoGeneTics Information System; Marie-Paule Lefranc (marie-paule.lefranc@igh.cnrs.fr), University of Montpellier, France	www.imgt.org; RRID: SCR_012780
AbStar	Bryan Briney (briney@scripps.edu), The Scripps Research Institute	https://github.com/briney/abstar
Cellranger	10X Genomics	https://support.10xgenomics.com/single-cell-gene-expression/software/downloads/latest
Prism 8	GraphPad	https://www.graphpad.com/scientific-software/prism/
ForteBio Data Analysis software	Sartorius	https://www.sartorius.com/en
PyMOL V2.4.2	PyMOL by Schrödinger	https://pymol.org
UCSF Chimera	Pettersen et al., 2004	http://plato.cgl.ucsf.edu/chimera/; RRID: SCR_004097
FlowJo v.10	BD Life Sciences	https://www.flowjo.com/solutions/flowjo
NumPy	https://github.com/numpy/numpy	v1.20.3
SciPy	https://github.com/scipy/scipy	v1.6.0
scikit-learn	https://github.com/scikit-learn/scikit-learn	V0.24.2
Statsmodels	https://github.com/statsmodels/statsmodels	v0.12.2
Clustal Omega	http://www.clustal.org/omega/	v1.2.2
DiversityAnalyzer	https://immunotools.github.io/immunotools/diversity_analyzer.html	N/A
Sulfinator	https://web.expasy.org/sulfinator/	N/A
Iroki	https://www.iroki.net/viewer	N/A
IgDiscover	https://gkhlab.gitlab.io/igdiscover22/	V1.0.2
Byos^™^ (Version 5.5)	Protein Metrics Inc.	https://www.proteinmetrics.com/products/byonic/
XCalibur Version v4.2	Thermo Fisher	N/A
Orbitrap Fusion Tune application v3.1	Thermo Fisher	N/A
UCSF ChimeraX	Meng et al, 2023	https://www.cgl.ucsf.edu/chimerax/ ; RRID:SCR_015872
Relion 4.0	Kimanius et al., 2021	RRID:SCR_016274
EPU Multigrid	Thermo Fisher	N/A
CryoSPARC	Punjani et al 10.1038/nmeth.4169	https://cryosparc.com
UCSF Chimera	Pettersen et al 10.1002/jcc.20084	https://www.cgl.ucsf.edu/chimera/
Phenix	Liebschner et al 10.1107/S2059798319011471	https://phenix-online.org
PDBePISA	Krissinel et al 10.1016/j.jmb.2007.05.022	http://www.ebi.ac.uk/pdbe/pisa/
Coot	Emsley and Cowtan 10.1107/S0907444904019158	https://www2.mrc-lmb.cam.ac.uk/personal/pemsley/coot/
MolProbity	Chen et al 10.1107/S0907444909042073	http://molprobity.biochem.duke.edu
EMRinger	Barad et al 10.1038/nmeth.3541	https://github.com/fraser-lab/EMRinger
ABodyBuilder2	Abanades et al 10.1038/s42003-023-04927-7	https://opig.stats.ox.ac.uk/webapps/sabdab-sabpred/sabpred/abodybuilder2/
**Other**		
0.2 um membrane filters	Fisher Scientific	Cat# 564-0020
Steriflip^™^ Vacuum Filter Units	MilliporeSigma	Cat# SCGP00525
Superdex 200 Increase10/300 GL column	GE Healthcare	Cat# GE28-9909-44
Superose 6 Increase 10/300 GL	GE Healthcare	Cat# GE29-0915-96
Protein A Sepharose	Cytiva	Cat# 17127903
Protein G Sepharose	Cytiva	Cat# 17061805
TOYOPEARL AF-Tresyl-650M	TOSOH	Cat# 0014472
Galanthus nivalis lectin (snow drop), agarose bound	Vector Labs	Cat# AL-1243-5
ProA Sensor	Sartorius	Cat# 18-5012
SA Sensor	Sartorius	Cat# 18-5020
RNeasy Plus Mini Kit	Qiagen	Cat# 74134
Dnase 1	Qiagen	Cat# 79254
Streptavidin-AF488	Thermo Fisher	Cat# S32354
Streptavidin-AF647	Thermo Fisher	Cat# S21374
Streptavidin-BV421	BD Biosciences	Cat# 563259
FVS510 Live/Dead stain	Thermo Fisher Scientific	Cat# L34966
Chromium Next GEM Single Cell 5’ Kit v2	10X Genomics	Cat# 1000253
Chromium Next GEM Chip K Single Cell Kit	10X Genomics	Cat# 1000286
NexttSeq 1000/2000 P1 Reagent Cartridge	Illumina	Cat# 20072323
E-Gel 96 2% with SYVR Safe	Fisher Scientificd	Cat# G720802
Econo-pak column	Biorad	Cat# 7321010
100kDA Ultra centrifugal filter unit	Millipore	Cat# UFC910024
50kDA Ultra centrifugal filter unit	Millipore	Cat# UFC905024
10kDA Ultra centrifugal filter unit	Millipore	Cat# UFC901024
Pierce Fab Preparation Kit	Fisher Scientific	Cat# 44985
cryovials	Globe Scientific	Cat# 3010
Sterile vacutainers	DB vacutainer	Cat# 364606
cryovial	Sarstedt	Cat# 72.694.396
ViaStain AOPI solution	Revvity	Cat# CS2-0106-25ml
Cell strainer	Falcon	Cat# 352360
5 ml round bottom tube	Corning	Cat# 352058
C18 ZipTip	Merck Milipore	Cat# ZTC18S008
Oasis HLB 96-well μElution Plate	Waters	Cat# 186001828BA
Vivaspin 500, 3 kDa MWCO, Polyethersulfone	Sigma-Aldrich	Cat# GE28-9322-18
Orbitrap Fusion mass spectrometer	Thermo Fisher Scientific	N/A
Easy nLC 1200	Thermo Fisher Scientific	N/A
EasySpray PepMap RSLC C18 column (75 μm x 75 cm)	Thermo Fisher Scientific	Cat# ES805
Acclaim^™^ PepMap^™^ 100 C18 HPLC Trap column	Thermo Fisher Scientific	Cat# 164946
Electron microscopy copper mesh grids	Electron Microscopy Sciences	Cat# EMS400-Cu
Whatman #1 filter paper	Cytiva	Cat# 1001-320
1.2/1.3 C-Flat holey carbon grids	EMS	Cat# CF313-100
n-Dodecyl-B-D-maltoside (DDM)	GoldBio	Cat# DDM5

## Data Availability

The cryo-EM structures of the HIV trimer alone or in complex with mAbs have been submitted to the Electron Microscopy Data Bank (EMDB) and Protein Data Bank. PDB and EMDB accession numbers are provided in the key resource table. The EMPEM data has been submitted to EMDB, and accession numbers are available in the Key Resource Table. All sequencing data generated in this study have been deposited in the NCBI and SRA accession numbers are provided in the Key Resources Table. All the data are publicly available from the date of publication, and relevant accession numbers are listed in the Key Resources Table. No new code was generated in this study. All data supporting the findings are included in the published article and its supplementary materials. Additional raw data can be provided by the lead contact upon request.

## HIGHLIGHTS

- Engineered Q23-APEX-GT2 trimer to stimulate diverse V2-apex bnAb B cell precursors
- Q23-APEX-GT2 prime diverse rare V2-apex bnAb B cell lineages in outbred macaques
- Q23-APEX-GT2 alone induces V2-apex antibodies with modest HIV neutralization breadth
- Structures confirm bnAb site targeting and binding modes mirroring human V2-apex bnAbs
