# Supplementary material for: Germline-targeting HIV immunogen induces cross-neutralizing antibodies in outbred macaques
Source: Immunity. Author manuscript; Available in PMC 2026 Jun 17. (PMC13274938; doi:10.1016/j.immuni.2026.03.012)
Supplement: 1 — Dataset S1 - CryoEM data collection and processing, related to Figure 1 and Figure 6. [file NIHMS2174337-supplement-1.pdf]

SUPPLEMENTARY FIGURES AND LEGENDS

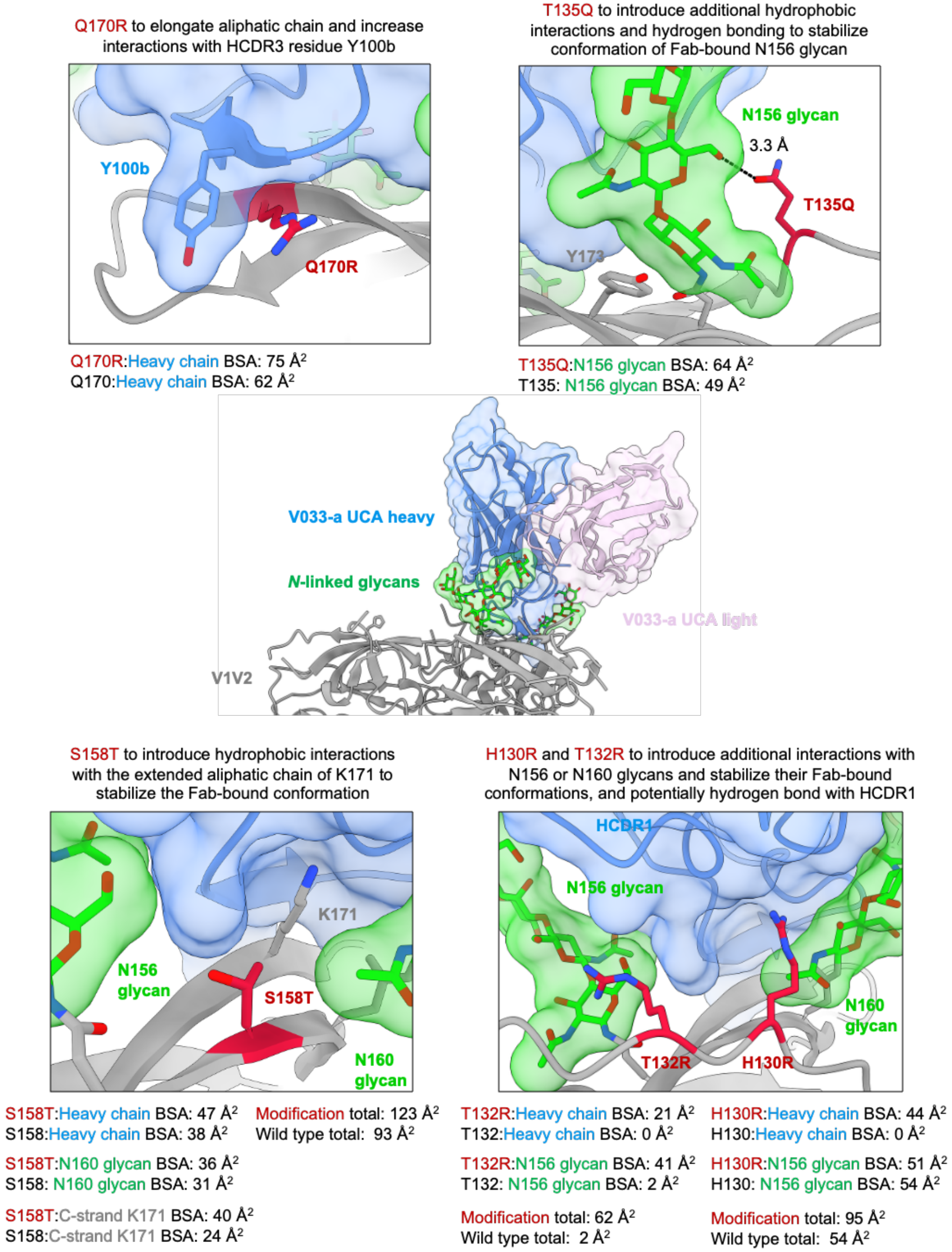

Figure S1. Antibody-guided design of V2-apex germline-targeting modifications. Related to Figure 1.
The cryo-EM structure of the rhesus bnAb lineage V033-a unmutated common ancestor (UCA) in complex with Q23.17 MD39 envelope trimer was used to rationally design V2-apex germline-targeting modifications (PDB-9OMG) (Habib et al, 2025 *submitted*). Expanded view in middle

diagram highlights V2-apex binding orientation of the V033-a UCA Fab and contacted apical glycans, both of which are shown with transparent surface representation and colored according to labels (heavy chain, blue; light chain, pink; glycans, green). Insets depict the local environments of select mutations, which are shown in stick representation and colored red, to highlight examples of germline-targeting modifications engineered to increase the affinity of envelope for the V033-a UCA. The surface areas buried (BSA; in $Å^2$) by modeled modifications (red) are compared to the template wild type residues (not shown) below each panel.

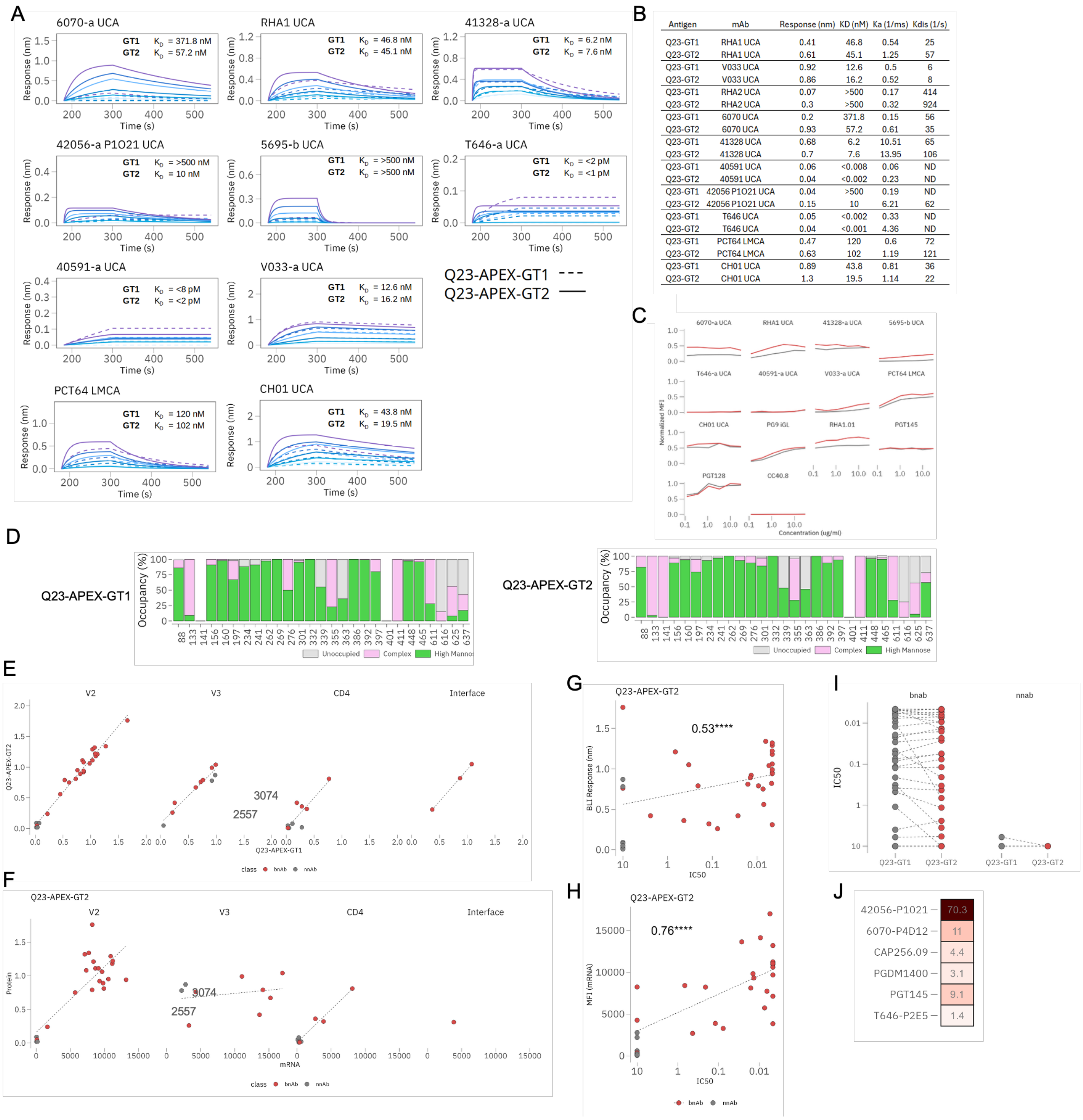

Antigen	mAb	Response (nm)	KD (nM)	Ka (1/ms)	Kdis (1/s)
Q23-GT1	RHA1 UCA	0.41	46.8	0.54	25
Q23-GT2	RHA1 UCA	0.61	45.1	1.25	57
Q23-GT1	V033 UCA	0.92	12.6	0.5	6
Q23-GT2	V033 UCA	0.86	16.2	0.52	8
Q23-GT1	RHA2 UCA	0.07	>500	0.17	414
Q23-GT2	RHA2 UCA	0.3	>500	0.32	924
Q23-GT1	6070 UCA	0.2	371.8	0.15	56
Q23-GT2	6070 UCA	0.93	57.2	0.61	35
Q23-GT1	41328 UCA	0.68	6.2	10.51	65
Q23-GT2	41328 UCA	0.7	7.6	13.95	106
Q23-GT1	40591 UCA	0.06	<0.008	0.06	ND
Q23-GT2	40591 UCA	0.04	<0.002	0.23	ND
Q23-GT1	42056 P1O21 UCA	0.04	>500	0.19	ND
Q23-GT2	42056 P1O21 UCA	0.15	10	6.21	62
Q23-GT1	T646 UCA	0.05	<0.002	0.33	ND
Q23-GT2	T646 UCA	0.04	<0.001	4.36	ND
Q23-GT1	PCT64 LMCA	0.47	120	0.6	72
Q23-GT2	PCT64 LMCA	0.63	102	1.19	121
Q23-GT1	CH01 UCA	0.89	43.8	0.81	36
Q23-GT2	CH01 UCA	1.3	19.5	1.14	22

Figure S2. Engineered Q23-APEX-GT2 shows broad and enhanced binding to unmutated common ancestor versions of rhesus and human V2-apex bnAb. Related to Figure 1.

A. Bio-Layer Interferometry (BLI) binding curves for Q23-APEX-GT1 (dashed lines) and Q23-APEX-GT2 (solid lines) trimers against selected V2-apex unmutated common ancestors (UCAs) as Fabs. Fabs were tested at five two-fold serial dilutions, starting from a maximum concentration of 10 µg/ml. The highest concentration (10 µg/ml) is depicted in violet, while the lowest concentration (0.625 µg/ml) is shown in light blue. Binding kinetics were assessed to determine affinity and potential differences in epitope engagement between the engineered Q23-APEX-GT2 and the base construct, Q23-APEX-GT1 germline-targeting (GT) immunogens. BLI binding sensograms traces depict association and dissociation phases, with relative binding strength inferred from response units over time.

B. K_D, K_{on}, and K_{off} values with the maximum binding response (nm) values are shown as a table for all 10 UCAs with Q23-APEX-GT1 and Q23-APEX-GT2 trimers (from panel A).

C. Antigenic assessment of membrane-bound Q23-APEX-GT1 and Q23-APEX-GT2 was conducted using a cell surface binding assay. The trimer immunogens were expressed on the cell surface via the wild-type (WT) Q23 transmembrane domain, mimicking their native presentation on viral membranes. Antibody binding was measured across a dilution series, starting at 10 μg/ml, to compare binding efficiencies and potential epitope accessibility in the transmembrane bound trimer context.
D. Glycan profile characterization of Q23-APEX-GT1 and Q23-APEX-GT2, assessed through proteomics-based site-specific glycan analysis (SSGA). The composition of glycans at key glycosylation sites was analyzed, highlighting variations in glycan processing at the V2-apex glycans between the two constructs. High-mannose glycans are represented in faded green, complex-type glycans in light pink, and unoccupied glycosylation sites in gray.
E. Antigenic characterization of Q23-APEX-GT2 and Q23-APEX-GT1 trimers in their soluble protein forms was evaluated using biolayer interferometry (BLI) against a comprehensive panel of broadly neutralizing antibodies (bnAbs, n = 36) and non-neutralizing antibodies (nnAbs, n = 11) targeting the four canonical bnAb site on the trimer. The binding profiles indicate that both immunogens exhibit overall similar antigenic properties (R^2 = 0.98, P <0.0001), suggesting retention of key epitope structures. V3 nnAb targeting the linear V3 peptide (3074 and 2557) are highlighted.
F. Antigenic characterization of Q23-APEX-GT2 in its soluble protein form versus its membrane-bound presentation via mRNA expression. The soluble protein was analyzed by BLI, while the mRNA-expressed antigen was evaluated using a cell surface binding assay. Antibody binding readouts at 10 μg/ml were used for direct comparison between the two formats, highlighting comparable antigen exposure and accessibility when presented as a recombinant protein versus a cell-surface expressed antigen (R^2 = 0.72, P <0.0001). For V3 bnAb, mRNA expressed trimer showed higher binding. V3 nnAb 3074 and 2557 are highlighted (similar to panel A).
G. Correlation plots for neutralization (IC_{50}) versus BLI binding to soluble antigen, and (H). IC_{50} versus binding to cell surface expressed antigen shows a significant positive correlation (Pearson's coefficient > 0.5). Four asterisks denote P value less than 0.0001. UCAs showed no neutralization at 10 μg/ml and are not shown.
I. Q23-APEX-GT2 showed a reduction in IC_{50} (suggesting a slightly more resistant neutralization profile) for a few bnAbs targeting the V2-apex and CD4bs. (J). Fold reduction in IC_{50} values for several V2-apex bnAbs (from both human and rhesus) was observed for Q23-APEX-GT2 pseudoviruses compared to Q23-APEX-GT1, suggesting the acquisition of a difficult-to-neutralize phenotype. Neutralization assays were performed using a large panel of 70 monoclonals spanning several bnAbs and non-nAbs (starting at 10 μg/ml) targeting all major epitopes against both Q23-APEX-GT1 and Q23-APEX-GT2 pseudoviruses.

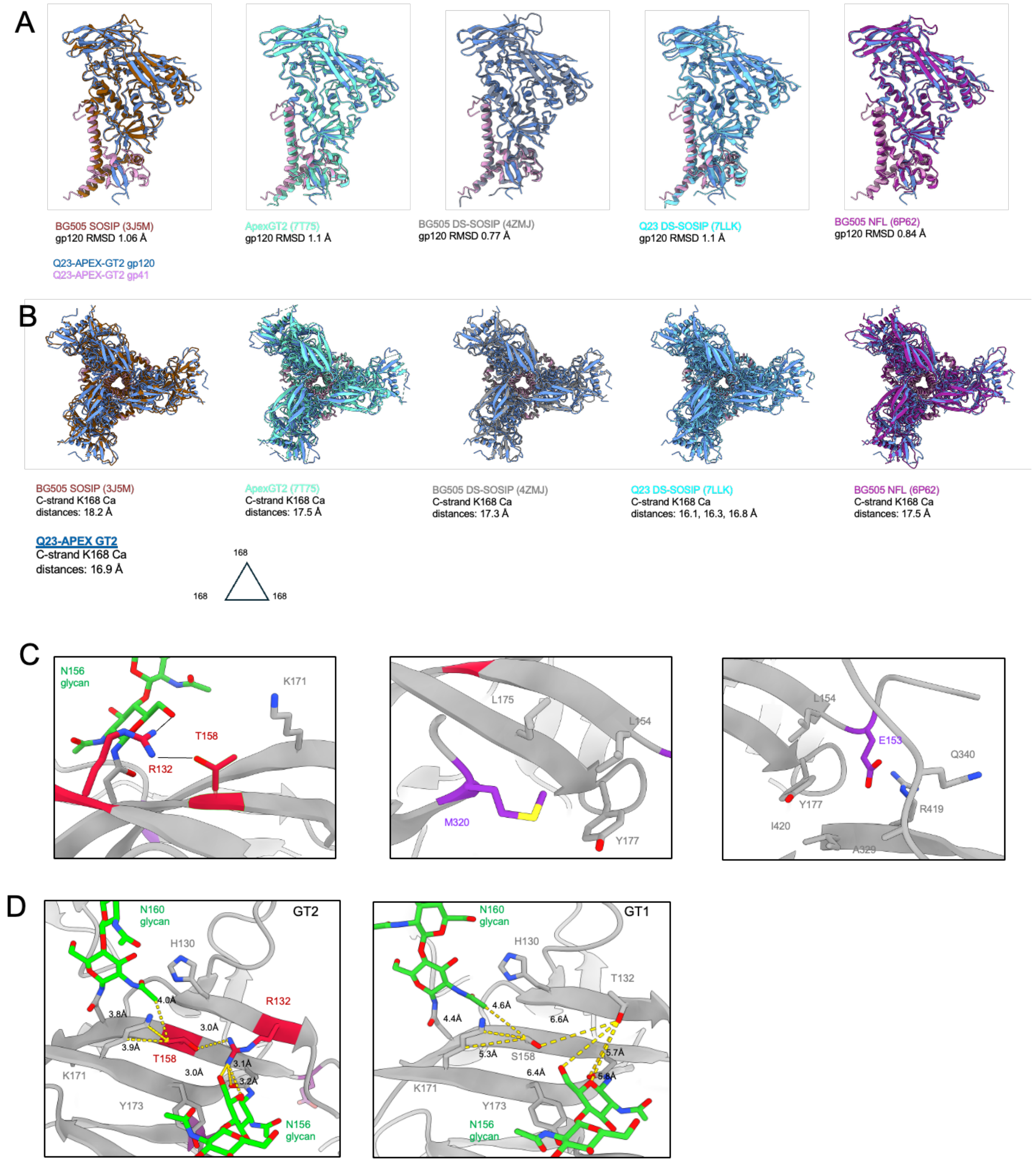

Figure S3. Structural alignments and details for Q23-APEX-GT2's V2-apex bnAb site. Related to Figure 1.

A. Superimpositions of a single protomer from Q23-APEX-GT2 with a protomer from the indicated envelope trimer structures by gp120 alignment.

B. Superimpositions of Q23-APEX-GT2 with the indicated envelope trimers achieved by gp120 alignment. Prefusion-closed trimer distances are measured from the alpha carbon (Ca) of K168 residues between adjacent C-strands.

C. Local environments of select GT1 (purple) and both GT2 (red) modifications in Q23-APEX-GT2. T132R forms hydrogen bonds with N156 glycan and S158T. The extended aliphatic chain

of T132R makes hydrophobic interactions with N156, while the methyl group of S158T makes hydrophobic interactions with K171. T320M fills a hydrophobic pocket lined by V2 residues L154, L175, and Y177. G153E inserts into a large pocket formed by L154, Y177, A329, Q340, R419, and I420.

D. Comparison of distance measurements (yellow dashes) between GT2 modifications (red) with “network residues” (left panel) and the same residue positions in GT1 (right panel).

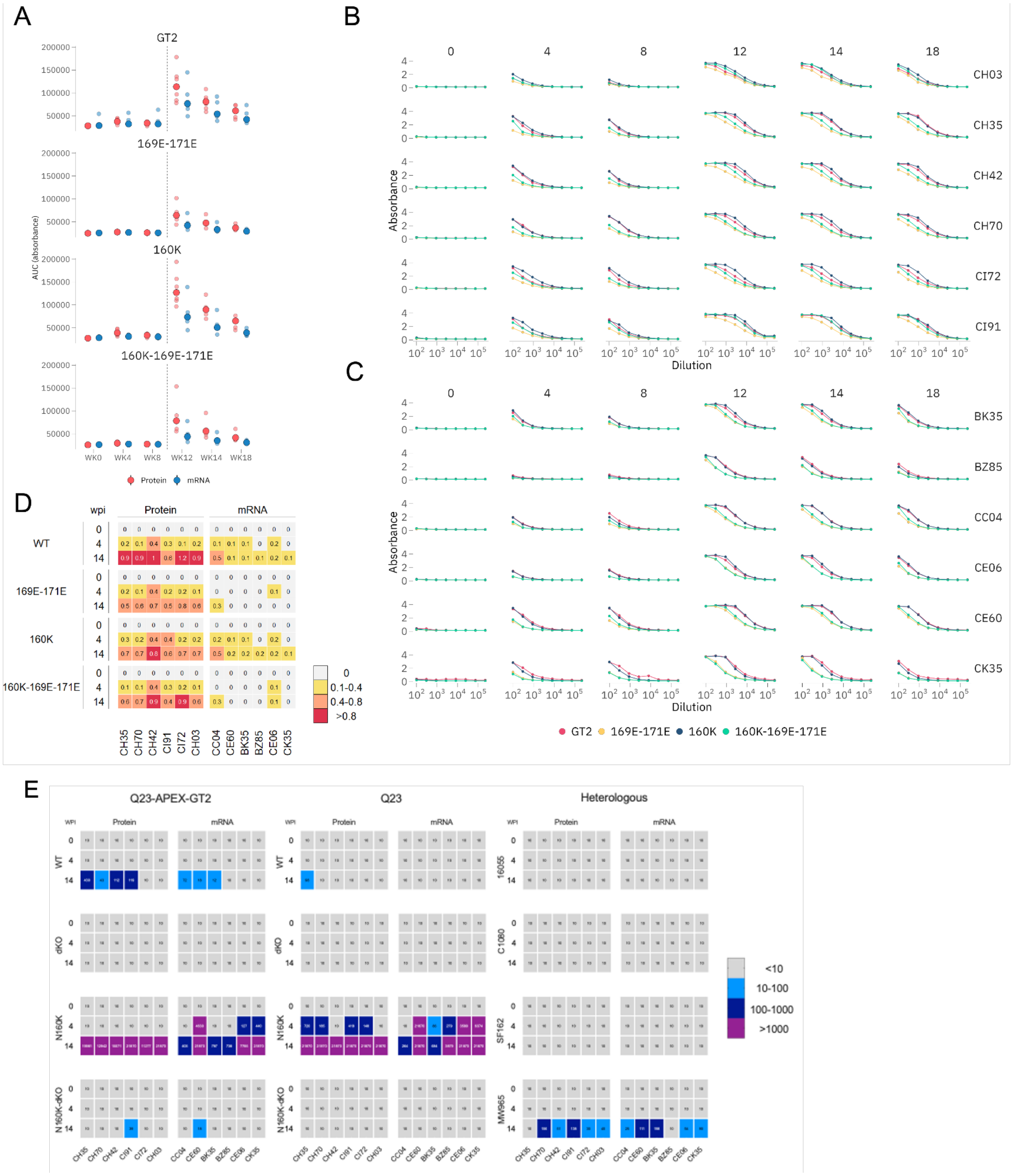

Figure S4. Longitudinal analysis of antigen and V2-apex epitope specific serum antibody responses in Q23-APEX-GT2 protein and mRNA vaccinated rhesus monkeys. Related to Figure 3.

A. Binding of sera from immunized rhesus macaques to Q23-APEX-GT2 was evaluated using Enzyme-Linked Immunosorbent Assay (ELISA) starting at a serum dilution of 1:100. The data shows the serum titers over time, with a substantial increase in binding titers observed after the boost at week 10, indicated by the dotted line (panel A) with associated curves shown in panels B and C. Both the protein and mRNA immunized groups demonstrated increased titers post-

boost. However, the protein-immunized group exhibited superior binding titers compared to the mRNA group, and both groups showed a decrease in titers as the study progressed, suggesting a waning immune response over time.

B. Binding against Q23-APEX-GT2 and its epitope knockout variants (169E-171E, 160K, 160K-169E, 169E) from protein immunized rhesus macaques were tested with ELISA starting at serum dilution of 1:100 at week 0 (pre-immunization), 4, 8, 12, 14, 18. Absorbance at OD450 is shown.

C. Binding against Q23-APEX-GT2 and its epitope knockout variants (169E-171E, 160K, 160K-169E, 169E) from mRNA immunized rhesus macaques were tested with ELISA starting at a serum dilution of 1:100 at week 0 (pre-immunization), 4, 8, 12, 14, 18. Absorbance at OD450 is shown.

D. Biolayer Interferometry (BLI) was used to assess serum polyclonal IgG binding to Q23-APEX-GT2 and its epitope knockout variants (169E-171E, 160K, 160K-169E-171E) at weeks 0, 4, and 14. Both immunization groups developed Q23-APEX-GT2-specific antibody responses, with titers significantly higher in the protein-immunized group compared to the mRNA-immunized group. Epitope-dependent IgG responses were observed in both groups, with binding kinetics showing greater sensitivity to epitope knockouts, particularly in the mRNA group. Notably, the mRNA-immunized group exhibited a stronger reliance on specific epitope recognition, likely due to its lower overall polyclonal antibody titers compared to the protein-immunized group.

E. Heatmap depicting serum neutralization of pseudoviruses carrying Q23-APEX-GT2 mutations (132R-158T), the Q23.17 WT virus, and various V2-apex bnAb epitope variants (169E-171E [dKO], 160K, and 160K-169E-171E [tKO]) using immune sera collected at weeks 0, 4, and 14 from Q23-APEX-GT2 protein- or mRNA-LNP-vaccinated monkeys. Neutralization was dependent on strand C residues, as evidenced by the loss of neutralization with the 169E-171E variant. Notably, immune sera exhibited increased neutralization upon 160K glycan knockout, which was completely abrogated when strand C epitopes were disrupted in the N160K backbone (160K-169E-171E), reinforcing the role of strand C in V2-apex-targeted neutralization.

Neutralization was also assessed against heterologous tier 2 HIV isolates (16055, C1080, and chimpanzee CAM13RRK, which shares the V2-apex site with HIV). Except for the protein-vaccinated monkey CH35, which exhibited some neutralization against CAM13KKR virus, no neutralization was detected. Additionally, two tier 1 viruses, SF162 and MW965, were tested. While most immune sera from both groups neutralized MW965, no neutralization was observed against SF162.

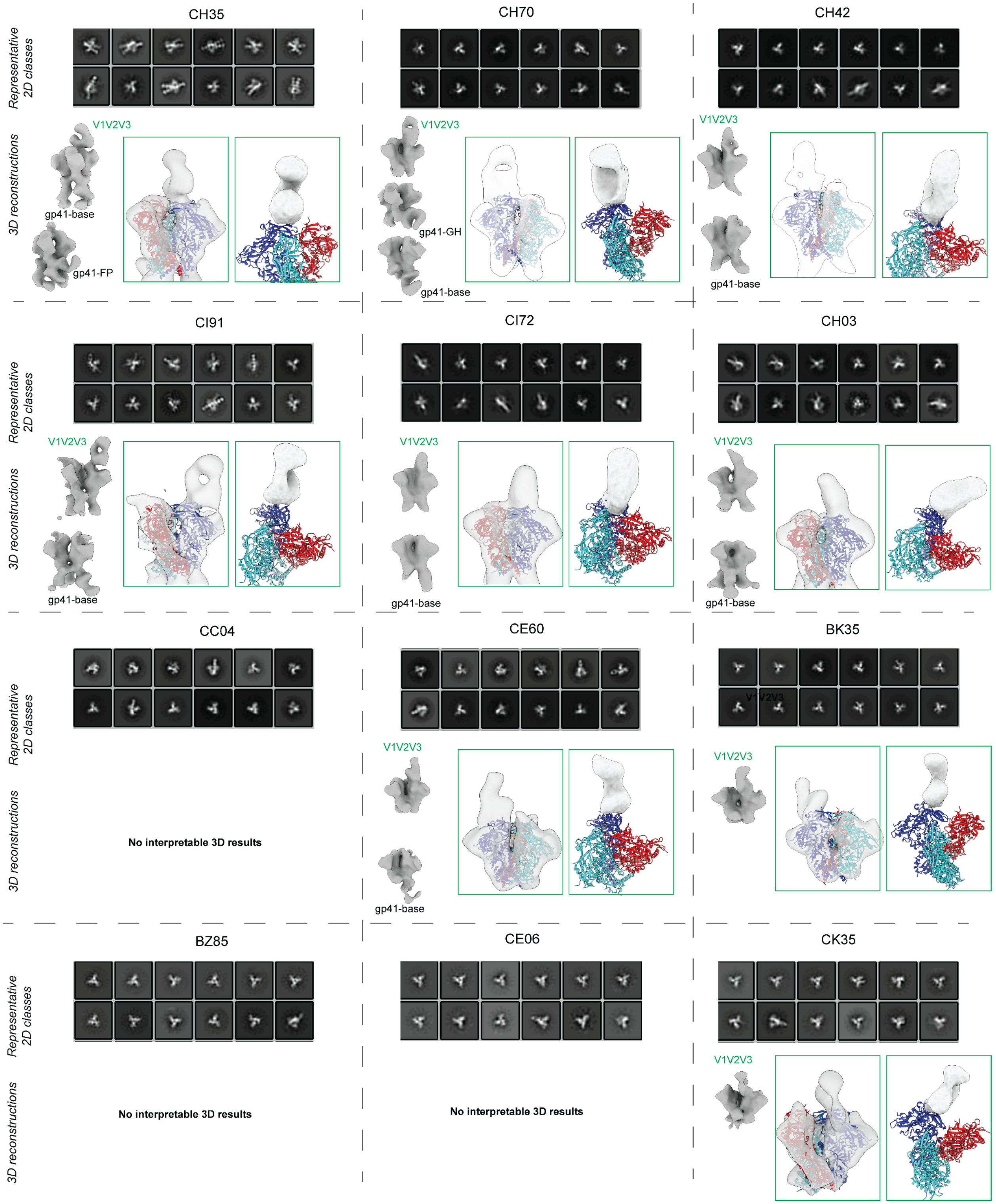

Figure S5. EMPEM 2D classes and 3D reconstructions. Related to Figure 3.
Representative 2D classes of all immune complexes and final 3D reconstructions used to generate the segmented composite maps in Figure 3E are displayed for all 12 EMPEM samples. For datasets where too few Fab-bound particles were present for accurate 3D reconstruction, the label “no interpretable 3D results” is used. For EMPEM samples with visible apex (V1V2V3

regions) Fab densities, additional views with fitted SOSIP trimer coordinates (each protomer colored differently) and segmented Fab map are shown in the green boxes.

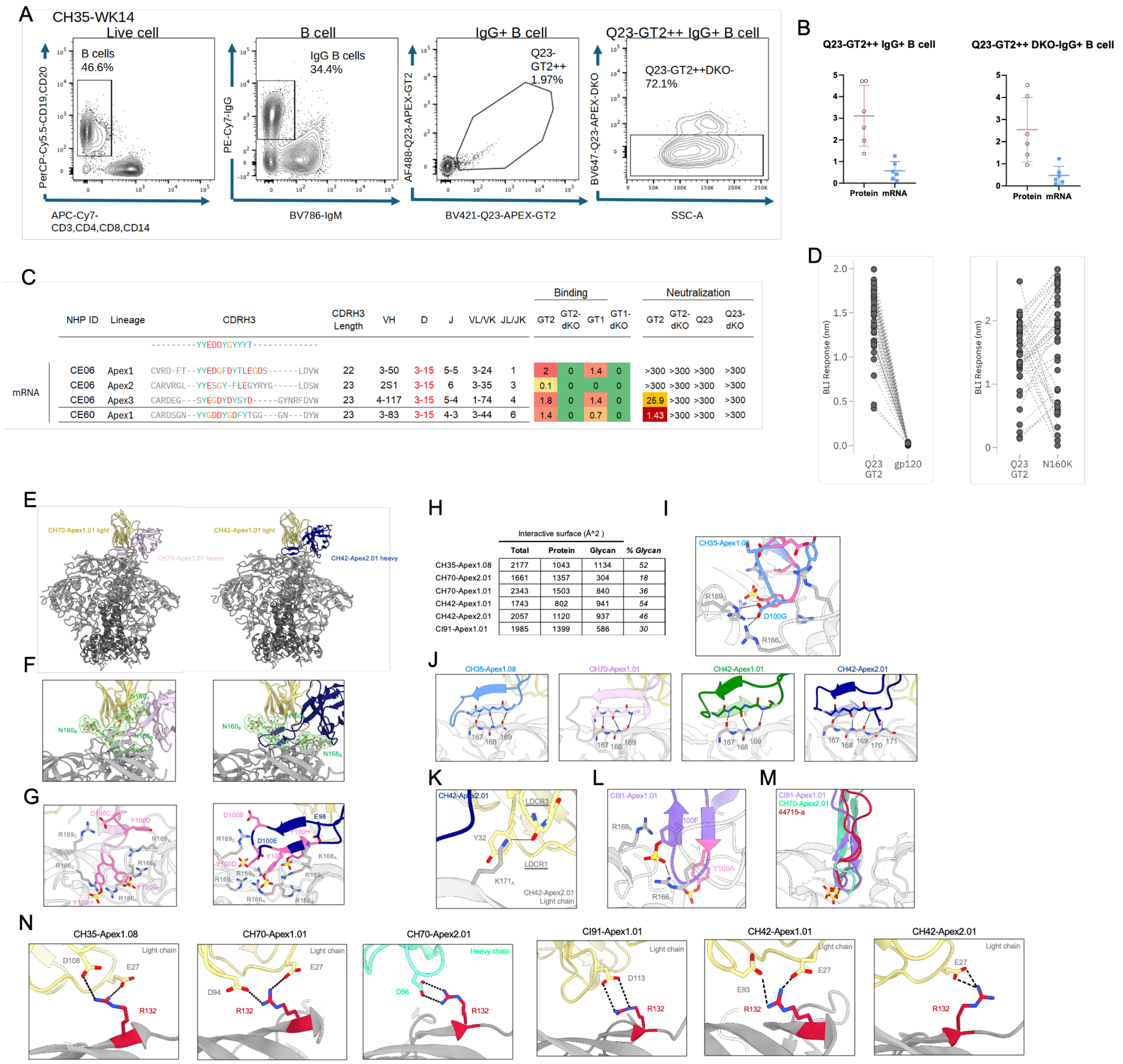

	NHP ID	Lineage	CDRH3	CDRH3 Length	VH	D	J	VL/VK	JL/JK	Binding GT2	Binding GT2-dKO	Binding GT1	Binding GT1-dKO	Neutralization GT2	Neutralization GT2-dKO	Neutralization Q23	Neutralization Q23-dKO
			---------YYEDDYGYYYT-------------														
mRNA	CE06	Apex1	CVRD-FT--YYEDGFDYTLEGDS------LDVW	22	3-50	3-15	5-5	3-24	1	2	0	1.4	0	>300	>300	>300	>300
	CE06	Apex2	CARVRGL--YYESGY-FLEGYRYG-----LDSW	23	2S1	3-15	6	3-35	3	0.1	0	0	0	>300	>300	>300	>300
	CE06	Apex3	CARDEG---SYEGDYDYSYD-----GYNRFDVW	23	4-117	3-15	5-4	1-74	4	1.8	0	1.4	0	25.9	>300	>300	>300
	CE60	Apex1	CARDSGN--YYGDDYGDFYTGG---GN---DYW	23	3-83	3-15	4-3	3-44	6	1.4	0	0.7	0	1.43	>300	>300	>300

	Interactive surface (Å^2) Total	Protein	Glycan	*% Glycan*
CH35-Apex1.08	2177	1043	1134	*52*
CH70-Apex2.01	1661	1357	304	*18*
CH70-Apex1.01	2343	1503	840	*36*
CH42-Apex1.01	1743	802	941	*54*
CH42-Apex2.01	2057	1120	937	*46*
CI91-Apex1.01	1985	1399	586	*30*

Figure S6. Antigen and epitope-specific IgG B cell profiling and mAb characterization in Q23-APEX-GT2 protein and mRNA-vaccinated monkeys. Related to Figures 4, 5 and 6.

A. Representative flow cytometry plots depicting Q23-APEX-GT2 antigen specific or V2-apex epitope specific (Q23-APEX-GT2 positive and Q23-APEX-GT2-dKO, R169E-K171E double knockout mutant) IgG B cells in week 14 lymph nodes of Q23-APEX-GT2 trimer protein-immunized CH35 rhesus macaque (RM).

B. Scatter plots show the percentage of antigen-specific B cells (Q23-APEX-GT2^{++}) (upper panel) and epitope-specific B cells (Q23-APEX-GT2^{++}DKO^{-}) (lower panel) among IgG^{+} B cells. The protein-immunized group is shown in red; and the mRNA-immunized group is shown in blue.

C. CDRH3 sequence, length and gene assignments for V, D, J for heavy chain and V, J for light chains from representative long CDRH3 mAbs lineages from Q23-APEX-GT2 mRNA immunized rhesus macaques. Germline IGHD3-15 D-gene is shown in red and the germline encoded anionic CDRH3 residues are highlighted in color. From each animal, representative sequences from each expanded lineage are shown. For each mAb, maximum BLI binding responses and IC_{50} neutralizations are shown with Q23-APEX-GT1 and Q23-APEX-GT2 trimer proteins and their

corresponding virus and the strand C dKO variants and the WT Q23.17 (for Q23-APEX-GT1). All functional mAbs are dependent on V2-apex stand C core epitope.
D. BLI binding of isolated monoclonals from protein-immunized animals against the immunogen-matched protein trimer, Q23-APEX-GT2 (marked as GT2), its monomeric gp120 protein and its N160K glycan knockout variant. Complete loss of binding with gp120 monomers show trimer dependence of isolated antibodies. Varying dependence on N160 glycan was seen with some mAbs showing reduced binding while several mAbs showed enhanced binding in the absence of N160 glycan. Several mAbs had little impact on binding in the absence of N160 glycan.
E. Overall structure of each respective lineage Fab in complex with the Q23-APEX-GT2 envelope trimer.
F. Expanded interface view from panel (a) to highlight binding position and interactions with apical envelope glycans. Glycans bound by each respective Fab are shown in stick representation with transparent surfaces.
G. Germline-encoded D3-15 residue interactions (dark pink) with core V2-apex epitope residues.
H. Quantification of the interactive surfaces for Fab:envelope cryo-EM structures determined in this study.
I. Hydrogen bonding with the mainchain of V2 apex C-strand residues by combined-mode and axe-like lineages.
J. CH35-Apex1.08 somatic mutant residue D100G (Y to D mutation) forms salt bridges with R166 and R169 from protomers A and C, respectively, completing recognition of these residue positions on all three protomers.
K. CH42-Apex2.01 engages C-strand residue K171 through mainchain LCDR3 hydrogen-bonding and LCDR1 hydrophobic interactions mediated by Y32.
L. CI91-Apex1.01 engages R166 and R169 from protomers C and A, respectively, via a second non-germline encoded sulfated tyrosine residue. The germline-encoded sulfated tyrosine residue, Y100A, is shown in pink for reference.
M. Structural superimposition of needle-like vaccine-elicited lineages with SHIV-elicited mature bnAb lineage 44715-a (PDB-9bnm) by gp120 alignment reveals sulfated tyrosines at the HCDR3 tips each align within the middle of the trimer.
N. Salt bridges formed between GT2 modification T132R and anionic residues from each vaccine-elicited lineage. Except for CH70-Apex2.01, all other lineages recognize T132R via the light chain.

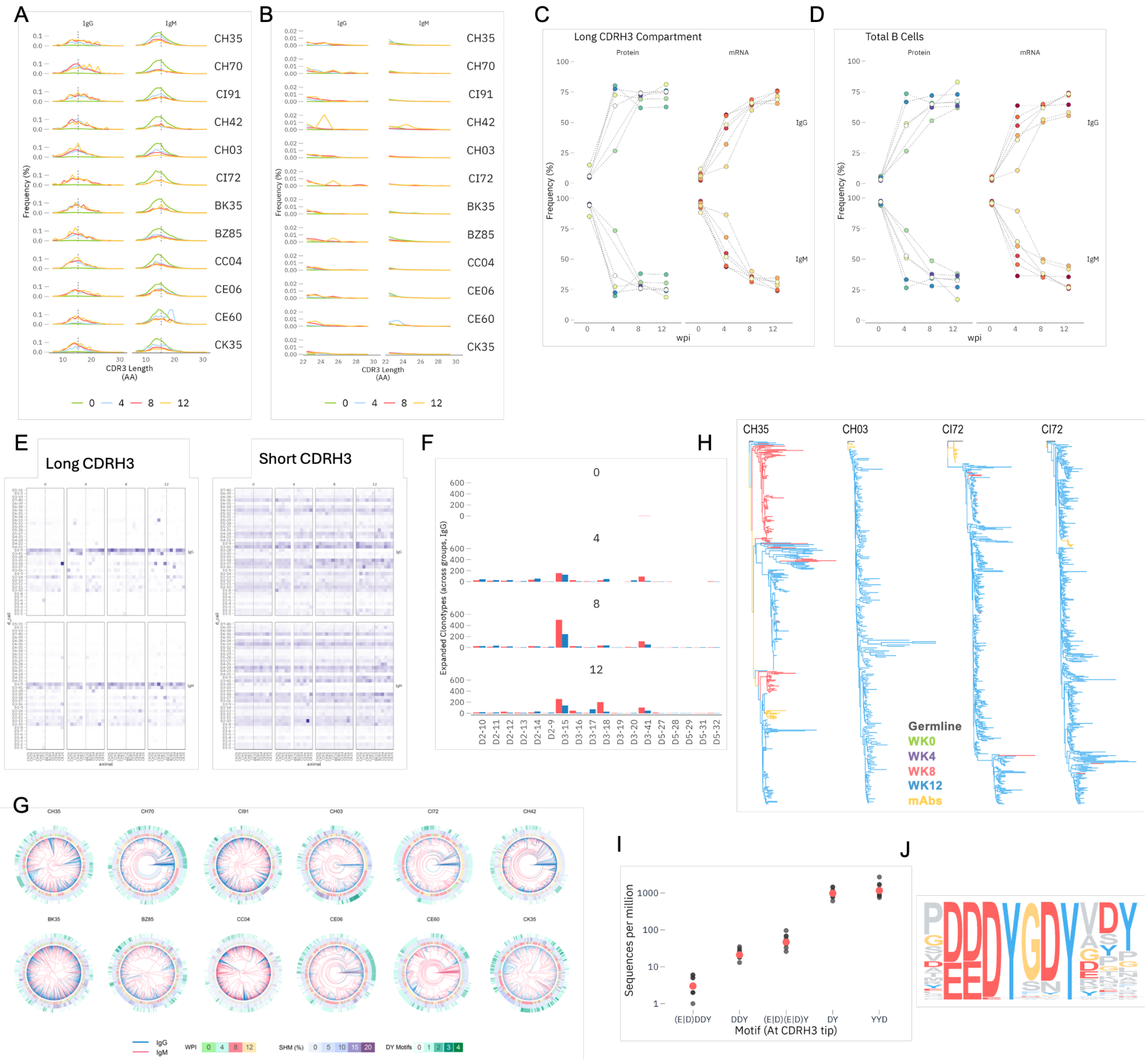

Figure S7. Longitudinal bulk BCR repertoire analysis of Q23-APEX-GT2 trimer immunized macaques show substantial expansion of long CDRH3 (≥22 amino acids) IgG B cell responses. Related to Figure 7.

A. Frequency distribution of CDRH3 lengths in the bulk BCR repertoire of each rhesus macaque at pre-immunization (week 0) and post-immunization timepoints (weeks 4, 8, and 12). Data are shown separately for the IgG and IgM compartments. The distribution at week 0 represents the baseline repertoire before antigen exposure, while post-immunization timepoints reflect the evolution of B cell responses in responses to immunization.

B. A magnified view of the subset of long CDRH3 sequences ≥22 amino acids (AA) from panel A, highlighting their enrichment within the IgG compartment across all animals. Compared to the pre-immunization repertoire, longer CDRH3 sequences show an increased frequency in the IgG compartment over time, suggesting preferential selection and expansion of class-switched IgG B cell clones with long CDRH3 loops following immunization.

C. The frequency of class-switched B-cell receptors (BCRs) within the long CDRH3 compartment (≥22 AA) was analyzed in all immunized rhesus macaques, comparing protein and mRNA immunization regimens. Protein-immunized animals exhibited a substantial early shift towards

IgG+ BCRs within the long CDRH3 subset by week 4, with levels stabilizing through weeks 8 and 12. In contrast, mRNA-immunized animals displayed a more gradual increase in IgG+ BCRs within the long CDRH3 compartment, continuing to rise steadily until week 12, suggesting a delayed but progressive class-switching response in this group.

D. The frequency of class-switched BCRs in the total B-cell compartment demonstrated more heterogeneous profiles of IgG and IgM across individual animals, irrespective of immunization type. This variability highlights differential class-switching kinetics at the total B-cell level compared to the long CDRH3-specific compartment.

E. Frequency distribution of D-gene usage within B cell receptors (BCRs) categorized by long (≥22 AA, Long CDRH3) or short (≤21 AA, Short CDRH3) complementarity-determining region 3 of the heavy chain (CDRH3). A substantial enrichment of the IGHD3 gene family is observed across all animals, suggesting a preferential selection or expansion of this gene family in response to immunization. The distribution pattern indicates a bias toward specific D-gene segments contributing to the formation of longer or shorter CDRH3 loops, potentially influencing antigen binding characteristics.

F. D-gene usage within expanded clonotypes in Q23-APEX-GT2 protein (red) and mRNA (blue) group immunized animals highlights a notable increase in the frequency of IGHD3-15, IGHD3-18, and IGHD3-41 across both immunization groups. The differential usage of additional D-genes suggests a level of variability in BCR repertoire formation, potentially driven by antigen engagement and selection pressures unique to each immunization strategy. These findings imply that while certain D-genes are commonly enriched, the breadth of D-gene utilization varies between protein- and mRNA-based immunization.

G. Circular phylogenetic trees displaying expanded clonotypes with associated genotypic data for all immunized rhesus macaques. Each tree represents the evolutionary relationships among B cell receptor (BCR) sequences within individual animals over the immunization period. Branch lengths correspond to sequence divergence, with nodes colored according to immunoglobulin isotype (IgM, IgG). In protein-immunized animals, we observe a clear longitudinal shift towards IgG-dominated clonotypes, indicative of class-switch recombination and affinity maturation. This transition is accompanied by increasing somatic hypermutation (SHM) levels and a progressive enrichment of the anionic sulfation motif (DY) within the complementarity-determining regions (CDRs), suggesting selective pressure favoring V2-apex epitope engagement and maturation. In contrast, mRNA-immunized animals exhibit a distinct pattern, with expanded clonotypes remaining predominantly IgM-expressing, and fewer signs of advanced maturation. The limited increase in SHM and minimal enrichment of sulfation motifs imply a reduced capacity for V2-apex directed clonotype evolution.

H. Phylogenetic trees depicting additional monoclonal antibodies (mAbs) isolated from immunized animals (in yellow) from Figure 4F illustrate the presence of lineage members as early as week 8, indicating that these mAbs were seeded by the Q23-APEX-GT2 prime. The branching patterns and sequence relationships confirm the early establishment and diversification of the lineage following priming.

I. The frequency of charged motifs at the apical position in long CDRH3 loops (≥22 amino acids) within naïve human BCR repertoires is shown as sequences per million. The data demonstrates an increasing occurrence of these motifs as the stringency of the charged residues decreases. Specifically, the DY and YYD motifs appear more frequently than motifs containing an additional charged residue, such as EDDY or DDDY, suggesting selective constraints on excessive charge at the CDRH3 apex.

J. A sequence logo representation of human naïve BCR sequences containing motifs similar to the rhesus macaque IGHD3-15 germline-derived sequence (YEDDYGYYT). The sequence logo highlights the frequent presence of the EDDYG motif, while the downstream YYT residues appear less commonly, indicating that the YYT extension may not be as strongly conserved within the

human repertoire. This suggests commonalities and potential differences that may influence function and antigen driven selection between human and rhesus BCR repertoires.

Dataset S1

Dataset S1 - CryoEM data collection and processing, related to Figure 1 and Figure 6.

Table 1-CryoEM data collection, refinement and validation statistics, related to Figures 1 and 6.

Fig. 1-CryoEM validation of apo Q23-APEX-GT2, related to Figure 1.

Fig. 2-CryoEM validation of vaccine-elicited V2-apex lineages in complex with Q23-APEX-GT2. related to Figure 5.

Fig. 3-CryoEM validation of vaccine-elicited V2-apex lineages in complex with Q23-APEX-GT2 continued, related to Figure 5.

Dataset S1 Table 1. Cryo-EM data collection, processing, and refinement validation statistics.

	Apo Q23-APEX-GT2 envelope trimer	CH35-Apex1.08 Fab in complex with Q23-APEX-GT2	CI91-Apex1.01 Fab in complex with Q23-APEX-GT2	CH70-Apex2.01 Fab in complex with Q23-APEX-GT2	CH70-Apex1.01 Fab in complex with Q23-APEX-GT2	CH42-Apex1.01 Fab in complex with Q23-APEX-GT2	CH42-Apex2.01 Fab in complex with Q23-APEX-GT2
**PDB**	**9NVV**	**9NVW**	**9NVX**	**9NVY**	**9NVZ**	**9NW0**	**9NW1**
**EMDB**	**49865**	**49866**	**49867**	**49868**	**49869**	**49870**	**49871**
**Data collection & processing**							
Microscope	FEI Titan Krios	FEI Titan Krios	FEI Titan Krios	FEI Titan Krios	FEI Titan Krios	FEI Titan Krios	FEI Titan Krios
Camera	Gatan K3	Gatan K3	Gatan K3	Gatan K3	Gatan K3	Gatan K3	Gatan K3
Magnification	105,000x	105,000x	64,000x	105,000x	105,000x	105,000x	105,000x
Voltage (kV)	300	300	300	300	300	300	300
Electron dose ($e^-/Å^2$)	58	58	56	58	58	58	58
Defocus range (μm)	0.8 - 2.0	0.8 - 2.0	0.8 - 2.0	0.8 - 2.0	0.8 - 2.0	0.8 - 2.0	0.8 - 2.0
Pixel size (Å)	0.83	0.83	0.83	0.83	0.83	0.83	0.83
Micrographs collected	5,530	6,045	7,072	3,407	6,439	7,225	12,261
Software	cryoSPARC v3.2	cryoSPARC v3.2	cryoSPARC v3.2	cryoSPARC v3.2	cryoSPARC v3.2	cryoSPARC v3.2	cryoSPARC v3.2
Micrographs used	5,001	5,230	5,347	2,936	5,565	6,355	9,787
Total extracted particles	1,216,751	1,163,114	1,468,214	800,901	1,429,951	1,931,551	2,696,911
Refined particles	304,719	93,887	88,248	60,972	201,576	362,484	122,593
Symmetry imposed	C1	C1	C1	C1	C1	C1	C1
Map Resolution (Å)	2.9	3.2	3.0	3.8	3.0	2.9	3.1
FSC threshold	0.143	0.143	0.143	0.143	0.143	0.143	0.143
**Refinement & validation**							
Initial model used	7LLK	7LLK	7LLK	7LLK	7LLK	7LLK	7LLK
Software	Phenix 1.21	Phenix 1.21	Phenix 1.21	Phenix 1.21	Phenix 1.21	Phenix 1.21	Phenix 1.21
Number of residues							
Protein	1,716	1,955	1,957	1,956	1,959	1,953	1,955
Ligand	118	127	118	121	128	128	130
Map CC	0.87	0.83	0.81	0.79	0.85	0.85	0.81
R.m.s. deviations							
Bond lengths (Å)	0.008	0.007	0.008	0.004	0.005	0.008	0.005
Bond angles (°)	1.06	0.90	0.987	0.82	0.95	1.2	1.2
EMRinger score							
MolProbity score	1.48	1.43	1.64	1.48	1.32	1.29	1.39
Clashscore	4.1	3.4	5.7	4.3	2.9	3.1	3.5
Rotamer outliers (%)	0.26	0.35	0.75	0.29	0.17	0.41	0.75
Ramachandran plot							
Favored (%)	96.4	95.8	95.2	96.0	96.5	96.8	96.3
Allowed (%)	3.6	4.2	4.8	4.0	3.5	3.2	3.7
Outliers (%)	0	0	0.5	0	0	0	0

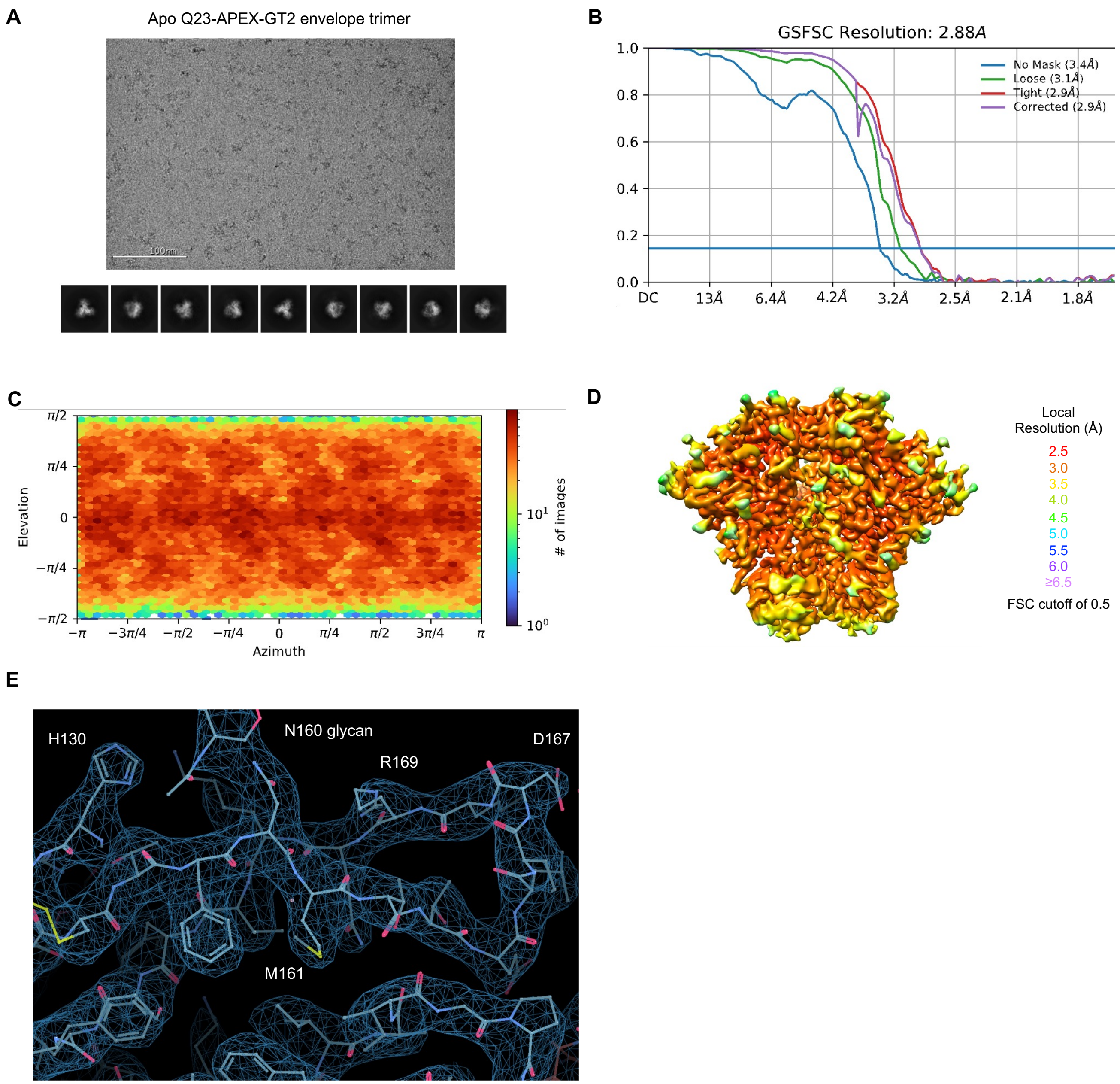

Dataset S1 Figure 1 | Single-particle cryo-EM validation for unbound Q23-APEX-GT2 envelope trimer, related to Figure 1.

a, Representative raw micrograph with representative 2D class averages of picked particles shown below. **b**, Orientations of all particles used in the final refinement are shown as a heatmap. **c**, Gold-standard fourier shell correlation (FSC) curves with auto-tightening using a non-uniform refinement with C1 symmetry. **d**, Local resolution estimation of the full map is shown as generated through cryoSPARC using an FSC cutoff of 0.5. **e**, Cryo-EM 3D reconstruction density to highlight residues and glycans at the trimer apex.

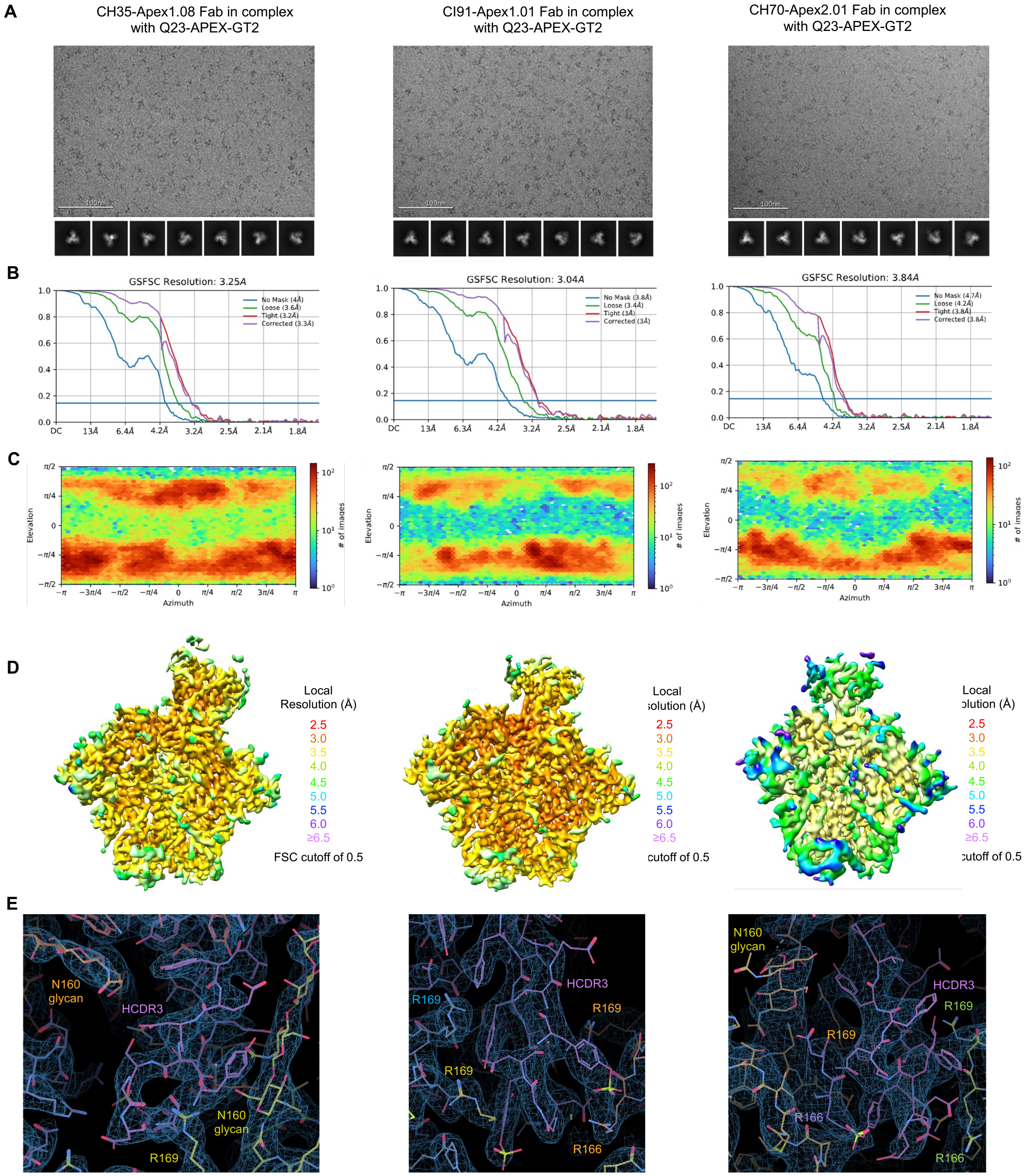

Dataset S1 Figure 3 | Single-particle cryo-EM validation for vaccine-elicited V2-apex lineages in complex with Q23-APEX-GT2 envelope trimer, related to Figure 5.

a, Representative raw micrograph with representative 2D class averages of picked particles shown below. **b**, Orientations of all particles used in the final refinement are shown as a heatmap. **c**, Gold-standard fourier shell correlation (FSC) curves with auto-tightening using a non-uniform refinement with C1 symmetry. **d**, Local resolution estimation of the full map is shown as generated through cryoSPARC using an FSC cutoff of 0.5. **e**, Example of high-resolution cryo-EM 3D reconstruction density to highlight the Fab HCDR3:trimer interface.

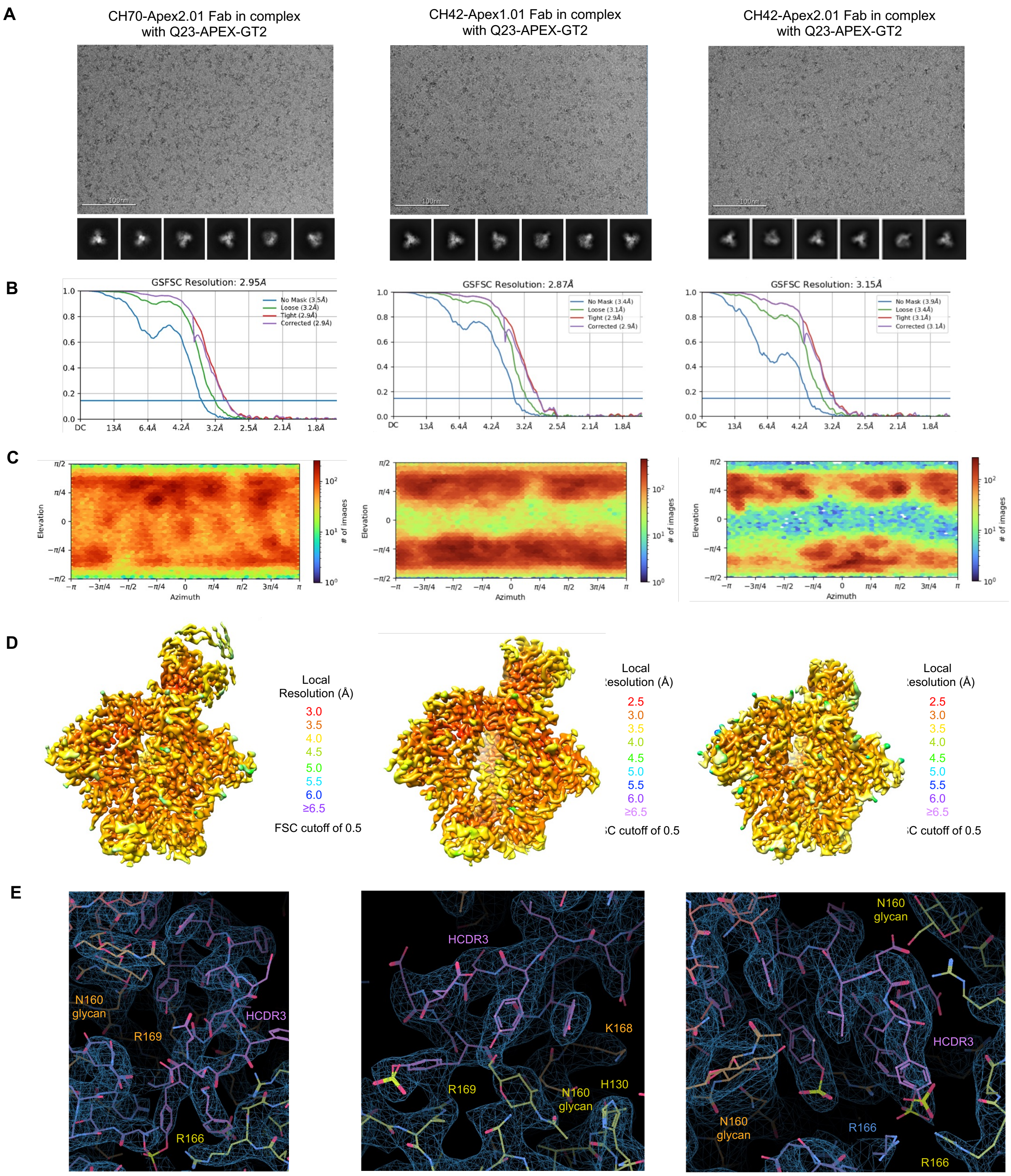

Dataset S1 Figure 4 | Single-particle cryo-EM validation for vaccine-elicited V2-apex lineages in complex with Q23-APEX-GT2 envelope trimer continued, related to Figure 5.

a, Representative raw micrograph with representative 2D class averages of picked particles shown below. **b**, Orientations of all particles used in the final refinement are shown as a heatmap. **c**, Gold-standard fourier shell correlation (FSC) curves with auto-tightening using a non-uniform refinement with C1 symmetry. **d**, Local resolution estimation of the full map is shown as generated through cryoSPARC using an FSC cutoff of 0.5. **e**, Example of high-resolution cryo-EM 3D reconstruction density to highlight the Fab HCDR3:trimer interface.
